# Estimate on the Dimension of the Singular Set of the Supercritical Surface Quasigeostrophic Equation

**DOI:** 10.1007/s40818-021-00093-3

**Published:** 2021-03-26

**Authors:** Maria Colombo, Silja Haffter

**Affiliations:** EPFL SB, Station 8, 1015 Lausanne, Switzerland

**Keywords:** Surface quasigeostrophic equation, Partial regularity, Singular set

## Abstract

We consider the SQG equation with dissipation given by a fractional Laplacian of order $\alpha <\frac{1}{2}$. We introduce a notion of suitable weak solution, which exists for every $L^2$ initial datum, and we prove that for such solution the singular set is contained in a compact set in spacetime of Hausdorff dimension at most $\frac{1}{2\alpha } \left( \frac{1+\alpha }{\alpha } (1-2\alpha ) + 2\right)$.

## Introduction

For $\alpha \in \big (0, \frac{1}{2}\big ]$ we consider the following fractional drift-diffusion equation

1 $$\begin{aligned} {\left\{ \begin{array}{ll} \partial _t \theta + u \cdot \nabla \theta = - (-\Delta )^\alpha \theta \\ {{\,\mathrm{div}\,}}u = 0, \end{array}\right. } \end{aligned}$$

where $\theta : {\mathbb {R}}^2 \times [0, \infty ) \rightarrow {\mathbb {R}}$ is an active scalar, $u: {\mathbb {R}}^2 \times [0, \infty ) \rightarrow {\mathbb {R}}^2$ is the velocity field and $(-\Delta )^\alpha$ corresponds to the Fourier multiplier with symbol $|\xi |^{2\alpha }$. The system is usually complemented with the initial condition

2 $$\begin{aligned} \theta (\ \cdot \ , 0) = \theta _0. \end{aligned}$$

We will be particularly interested in the surface quasigeostrophic (SQG) equation where the velocity field *u* is determined from $\theta$ by the Riesz-transform ${\mathcal {R}}$ on ${\mathbb {R}}^2$. More precisely, we require

3 $$\begin{aligned} u = \nabla ^\perp (-\Delta )^{-\frac{1}{2}} \theta = {\mathcal {R}}^\perp \theta . \end{aligned}$$

There is a natural scaling invariance associated to the system: whenever $(\theta , u)$ solves (1), then so does the pair

4 $$\begin{aligned} \theta _r(x,t) := r^{2\alpha -1} \theta (rx, r^{2\alpha }t) \qquad u_r(x,t)= r^{2\alpha -1} u(rx, r^{2\alpha }t). \end{aligned}$$

### Main Result

Our main result shows that for every $L^2$ initial datum and every $\alpha \in \big [\frac{9}{20}, \frac{1}{2}\big ),$ there exists an almost everywhere smooth solution of the SQG equation and, more precisely, it provides a bound on the box-counting and Hausdorff dimension of the closed set of its singular points.

#### Theorem 1.1

Let $\alpha _0:= \frac{1+\sqrt{33}}{16}$. For any $\alpha \in \big (\alpha _0, \frac{1}{2}\big )$ and any initial datum $\theta _0\in L^2({\mathbb {R}}^2)$ there is a Leray–Hopf weak solution $(\theta , u)$ of (1)–(3) (see Definition 3.1) and a relatively closed set $\mathrm{Sing}\, \theta \subset {\mathbb {R}}^2 \times (0, \infty )$ such that

- $$\theta \in C^{\infty }\big ([{\mathbb {R}}^2 \times (0, \infty ) ]\setminus \mathrm{Sing} \,\theta \big )$$,
- $\mathrm{Sing} \, \theta \,\cap \, [{\mathbb {R}}^2 \times [t, \infty )]$ is compact with box-counting dimension at most $\frac{1}{2\alpha } \left( \frac{1+\alpha }{\alpha } (1-2\alpha ) + 2\right)$ for any $t>0,$
- the Hausdorff dimension of $\mathrm{Sing}\, \theta$ does not exceed $\frac{1}{2\alpha } \left( \frac{1+\alpha }{\alpha } (1-2\alpha ) + 2\right) .$

#### Remark 1.2

We will in fact prove a slightly stronger statement, namely that all suitable weak solutions $\theta$ of (1)–(3) on ${\mathbb {R}}^2 \times (0, \infty )$ (see Definition 3.4) satisfy the estimate on the dimension of the spacetime singular set $\mathrm{Sing} \, \theta$; in particular, they are smooth almost everywhere in spacetime. Moreover, the set $\mathrm{Sing}\,\theta$ is compact as soon as the initial datum is regular enough to guarantee local smooth existence.

The regularity issue for the equation (1)–(3) is fully understood only in the subcritical and critical regime, namely for $\alpha \ge \frac{1}{2}$. The critical case (without boundaries) is now well-understood thanks to Kiselev, Nazarov and Volberg [21] and Caffarelli and Vasseur [3] (see also [11]) and one even has a description of the long time behaviour of the system [12, 13]. On bounded domains, the critical case has been well-studied in a series of works initiated by [8]. In the supercritical range $\alpha < \frac{1}{2},$ the global regularity of Leray–Hopf weak solutions to the SQG equation is an open problem related to the problem of global existence of classical solutions: in fact, it is well-known that Leray–Hopf weak solutions coincide with classical solutions as long as the latter exist. Constantin and Wu [9, 10] obtained partial results by extending the program of [3] to the supercritical regime. In [3] the technique of De Giorgi for uniformly elliptic equations with measurable coefficients is adapted to prove the smoothness of Leray–Hopf weak solutions in three steps: the local boundedness of $L^2$ solutions, the Hölder continuity of $L^\infty$ solutions, and the smoothness of Hölder solutions. While the $L^\infty$-bound for Leray–Hopf weak solutions still works in the supercritical case [9], only conditional regularity results are known regarding the second and third step of the scheme. For instance, Hölder solutions in $C^\delta$ are smooth for $\delta >1-2\alpha$, while for $\delta <1-2\alpha$ this is left open [10]. On the negative side, [1] established non-uniqueness of a class of (very) weak-solution for the system (1)–(3), even for subcritical dissipations. In this context Theorem 1.1 is, to our knowledge, the first a.e. smoothness / partial regularity result.

### An $\varepsilon$-Regularity Theorem

The estimate on the dimension of the singular set in Theorem 1.1 follows from a simple covering argument and a so-called $\varepsilon$-regularity result: in order to fix the main ideas, we present the latter in a simplified version in Theorem 1.3 below. In what follows we denote by $\theta ^*$ the Caffarelli–Silvestre extension of $\theta$ and by ${\mathcal {M}}$ the maximal function with respect to the space variable (see Sects. 2.4 and 2.6); $K_q$, as defined in (51), is a constant depending on the local-in-time $L^\infty _t L^q_x({\mathbb {R}}^2)$ estimate of $\theta$ (recalled in Sect. 3.1).

#### Theorem 1.3

Let $\alpha \in \big [\alpha _0, \frac{1}{2}\big )$, $q \ge 8$ and $p:=\frac{1+\alpha }{\alpha } + \frac{1}{q}.$ There exists a universal $\varepsilon = \varepsilon (\alpha )>0$ such that the following holds: Let $(\theta , u)$ be a suitable weak solution of (1)–(3) on ${\mathbb {R}}^2 \times (0,T)\,$ (see Definition 3.4) satisfying

5 $$\begin{aligned}&\frac{\Vert \theta \Vert _{L^\infty ({\mathbb {R}}^2 \times [t-r^{2\alpha }, t+r^{2\alpha }])}^{p-2}}{r^{p(1-2\alpha )+2}} \nonumber \\&\quad \Bigg ( \int _{{\mathcal {C}}^*(x,t;r)} y^b|{\overline{\nabla }} \theta ^* |^2 \, dz\, ds \, dy +\int _{{\mathcal {C}}(x,t;r)} {\mathcal {M}}\left( (D_{\alpha ,2}\, \theta )^2\right) \, dz \, ds \Bigg ) \le \varepsilon , \end{aligned}$$

where $${\mathcal {C}}(x,t;r):=B_{K_q r^{2\alpha -2/q}}(x) \times (t-r^{2\alpha }, t+r^{2\alpha }),$$ $${\mathcal {C}}^*(x,t;r):=[0,r)\times {\mathcal {C}}(x,t;r)$$ and

6 $$\begin{aligned} (D_{\alpha , 2} \, \theta ) (z, s):=\left( \int _{{\mathbb {R}}^2} \frac{|\theta (z, s)-\theta (z',s) |^2}{|z-z' |^{2+2\alpha }} \, dz' \right) ^{\frac{1}{2}}. \end{aligned}$$

Then $\theta$ is smooth on $B_{r/8}(x) \times (t-r^{2\alpha }/16,t + r^{2\alpha }/16).$

The integral quantities present in (5) are two non-equivalent localized versions of the dissipative part of the energy, i.e. the $L^2((0,T), W^{\alpha ,2}({\mathbb {R}}^2))$-norm of $\theta$, and are globally controlled through the latter. At this point, the careful reader will object that Theorem 1.3 cannot be used in a covering argument since the maximal function is not bounded on $L^1$. This issue represents a mere technical difficulty though: it is resolved by introducing a suitable variant of the sharp maximal function which leads to the more involved $\varepsilon$-regularity criterion of Corollary 6.6. Theorem 1.3 is a consequence of the $\varepsilon$-regularity Theorem 5.3 (which holds for every $\alpha \in (\frac{1}{4}, \frac{1}{2})$) whose smallness requirement features an $L^p$-based excess quantity and can be met at some small scale by requiring (5). Theorem 5.3 on the other hand is obtained via an excess decay result and a linearization argument, in analogy with [24] for the classical Navier–Stokes equations and with [7] for the hyperdissipative Navier-Stokes equations. Nevertheless there are some novelties in our approach with respect to the corresponding results for Navier-Stokes:

- Our $\varepsilon$-regularity result relies on the crucial observation (previously used in [3, 10]) that the equation (1) is invariant under a change of variables which sets the space average of *u* to zero. Indeed, the scaling (4), in contrast to the analogous situation for the Navier-Stokes equations, does not guarantee any control on the average of *u* on $B_r$ in terms of the average of the rescaled solution $u_r$ on $B_1$ as $r \rightarrow 0$. The lack of control on the averages introduces a challenge to iterate the excess decay, since at each step we need to correct for this change of variable, in a similar spirit to [10].
- As a second ingredient, we introduce a new notion of suitable weak solution which enables us to perform energy estimates of nonlinear type controlling a potentially large power of $\theta$. Such nonlinear energy estimates exploit the boundedness of Leray–Hopf weak solutions in an essential way and are not available for the Navier-Stokes equations. The freedom of choosing a suitable nonlinear power on the other hand is crucial in the context of the SQG equation: Indeed, the classical (local) energy controls naturally $\theta \in L^\infty ((0,T), L^2({\mathbb {R}}^2)) \cap L^2 ((0,T), W^{\alpha ,2}({\mathbb {R}}^2) )$ and hence, by interpolation, $\theta \in L^{2(1+\alpha )}({\mathbb {R}}^2 \times (0, T))$. Yet, since $2(1+\alpha ) <3$ for $\alpha < \frac{1}{2}$, this is not enough to conclude a strong enough Caccioppoli-type inequality which accounts for the cubic nonlinearity in the local energy.
- On one side, Theorem 1.3 may be seen as an analogue of Scheffer’s result [27] for Navier-Stokes, providing $\varepsilon$-regularity criterion at a fixed scale. On the other side, in order to give an estimate on the dimension of the singular set, the smallness (5) must be required in terms of differential quantities of $\theta$, as it happens in the more refined result by Caffarelli, Kohn and Nirenberg for Navier-Stokes [4]. In the context of the SQG equation, the easier Corollary 6.1 below may be seen as the full analogue of Scheffer’s result. Although Corollary 6.1 still establishes the compactness of the singular set, it does, in contrast to Navier-Stokes, not yield any estimate on the dimension of the singular set.

Using the “continuity” of the aforementioned $\varepsilon$-regularity Theorem 5.3 under strong convergence in $L^p$, an immediate consequence is the stability of the singular set in the fractional order $\alpha \in (\frac{1}{4}, \frac{1}{2}]$ which in particular recovers the following theorem of [35] (see also [5, 6] for related results in other contexts).

#### Corollary 1.4

(Global regularity for slightly supercritical SQG) Let $\theta _0 \in H^2({\mathbb {R}}^2)$ with $\Vert \theta _0\Vert _{H^2}\le R.$ Then there exists $\varepsilon = \varepsilon (R)>0$ such that (1)–(3) has a unique smooth solution $\theta \in L^\infty _{loc}([0, \infty ), H^2({\mathbb {R}}^2)) \cap L^2_{loc}((0, \infty ), H^{2+ \alpha }({\mathbb {R}}^2))$ for all fractional orders $\alpha \in \left[ \frac{1}{2}-\varepsilon , \frac{1}{2}\right] .$

#### Remark 1.5

The corollary could be set in any $H^{1+ \delta }({\mathbb {R}}^2)$ for $\delta >0$ which is subcritical for orders $\alpha$ close to $\frac{1}{2}$ and there admits a (quantified) short-time existence of smooth solutions. In [35] the assumption $\Vert \theta _0\Vert _{H^2}\le R$ is replaced by the scaling invariant assumption $\Vert \theta _0\Vert ^\alpha _{L^2}\Vert \theta _0\Vert ^{1-\alpha }_{\dot{H}^2}\le R.$ The latter statement can be reduced to ours by applying a first rescaling which renormalizes the $L^2$-norm of the initial datum to 1.

Moreover, by the decay of the $L^\infty$-norm of solutions (see Theorem 3.2 below), the $\varepsilon$-regularity criterion is verified for large times and we recover the eventual regularization of suitable weak solutions from $L^2$-initial data for $\alpha \in (\frac{1}{4}, \frac{1}{2})$ previously established for Leray–Hopf solutions in [30] for $\alpha$ close to $\frac{1}{2}$ and in [14, 20] for any $\alpha \in (0,\frac{1}{2})$.

### A Conjecture on the Optimal Dimension Estimate

Theorem 1.1 leaves open the question of whether or not the estimate on the dimension of the singular set, as well as the range of $\alpha$ for which it is valid, is optimal. We believe that a natural conjecture for an optimal estimate of the dimension of the spacetime singular set is

7 $$\begin{aligned} \dim _{\mathcal {P}}(\mathrm{Sing} \,\theta ) \le \frac{1}{2\alpha }(4-4\alpha ), \end{aligned}$$

and

8 $$\begin{aligned} \dim _{\mathcal {H}}(\mathrm{Sing} \,\theta ) \le \frac{1}{2\alpha }\big (4-4\alpha \big ). \end{aligned}$$

In (7), ${\mathcal {P}}$ is the parabolic Hausdorff measure that is, for $\alpha < \frac{1}{2},$ the Hausdorff measure resulting from restricting the class ${\mathcal {F}}$ of admissible covering sets to the spacetime cylinders ${\tilde{Q}}_r(x,t)= B_{r^{1/(2\alpha )}}(x) \times (t-r, t].$ We refer for instance to [7] for its construction for $\alpha > \frac{1}{2}$. The cylinders ${\tilde{Q}}_r(x,t)$ are the natural choice for $\alpha <\frac{1}{2}$ because their diameter is less than 4*r*, at difference from the classical parabolic cylinders $B_r(x) \times (t-r^{2\alpha }, t]$ whose diameter is of the order of $r^{2\alpha }$.

The conjecture (7) is based on a dimensional analysis of the equation : We may assign a “dimension” to any function $f(\theta )$ of $\theta$ via the exponent $\beta$ of the rescaling factor $1/r^\beta$ which makes the spacetime integral of $f(\theta )$ on ${\tilde{Q}}_r$ dimensionless, i.e. scaling-invariant with respect to (4). The number appearing on the right-hand side of (7) corresponds then to dimension of the energy, whose dissipative part is *the* globally controlled quantity in the form of a spacetime integral which scales best. This would correspond to the result of Caffarelli, Kohn and Nirenberg [4] ) for the Navier-Stokes system (see [7, 22, 33] for fractional dissipations of order $\alpha \in [\frac{3}{4}, \frac{5}{4})$) who proved that suitable weak solutions of the latter are smooth outside a closed set of dimension 1. In fact, for the Navier-Stokes system this bound on the dimension of the singular set is what the scaling of the equations and boundedness of the energy suggest.

Notice that the right-hand side of both (7) and (8) does not converge to 0 as $\alpha \rightarrow \frac{1}{2}$: this is due to the fact that the quantity that dictates the scaling-criticality of the equation, namely the $L^\infty$-norm of $\theta$, is not of integral type and hence cannot be used in a covering argument of the type that we do in the proof of Theorem 1.1. In turn, this covering argument finds his pivotal quantity in the dissipative part of the energy, which has a worse scaling than the $L^\infty$-norm of $\theta$.

In the proof of Theorem 1.1, it is natural to consider the classical Hausdorff measure, since the tilting effect of the change of variables, which sets the space average of *u* to zero, forces us to work on balls in spacetime (rather than parabolic cylinders, see Sect. 6.4 and in particular Step 3 of the proof of Corollary 6.6). This effect of the change of variables constitutes a serious obstacle for any parabolic Hausdorff dimension estimate. However, our estimate is nonoptimal: to obtain the optimal estimate, one should replace $\frac{1+\alpha }{\alpha }$ by 2 in the estimate of the dimension of the singular set in Theorem 1.1; however, the integrability exponent $\frac{1+\alpha }{\alpha }$ represents the least possible exponent for which we are able to use a “nonlinear” localized energy inequality in an excess decay argument (cf. Lemma 3.8). An analogous difficulty appears for the ipodissipative Navier-Stokes equations for low fractional orders $\alpha < \frac{3}{4}$ where the Caccioppoli-type inequality as described before fails to be strong enough to control the cubic nonlinearity and indeed no estimate of the dimension of the singular set is known.

### Structure of the Paper

The paper is structured as follows. After recalling some technical preliminaries in Sect. 2, we discuss in Sect 3 the global and local energy inequalities of the SQG equation and we define the notion of suitable weak solutions. The key compactness property of the latter is proven in Sect. 3.5 and leads to an excess decay result established in Sect. 4. The iteration of the excess decay on all scales is performed in Sect. 5 and requires to introduce a change of variables which sets to 0 the average of the velocity *u* on suitable balls. This excess decay yields the basis for several $\varepsilon$-regularity results, in particular Theorem 1.3, which are deduced in Sect. 6. The proof of Theorem 1.1 is given in Sect. 7. In Sect. 8, we discuss the stability of the singular set with respect to variations of the fractional order of dissipation.

## Preliminaries

### Notation

We use the following notation for space(time) averages of functions or vector fields *f* defined on ${\mathbb {R}}^2 \times [0, \infty )$: For bounded sets $E \subseteq {\mathbb {R}}^2 \times [0, \infty )$ and $F \subseteq {\mathbb {R}}^2,$ we define

$$\begin{aligned} (f)_E := \fint _{E} f(x,t) \, dx \,dt \qquad \text { and } \qquad [f(t)]_F :=\fint _{F} f(x,t) \, dx. \end{aligned}$$

We introduce the spacetime cylinder adapted to the parabolic scaling (4) of the equation

$$\begin{aligned} Q_r(x, t):=B_r(x) \times (t-r^{2\alpha }, t]. \end{aligned}$$

In the upper half-space ${\mathbb {R}}^3_+$ we define $B_r^*(x):=B_r(x) \times [0, r)$ and we define the extended cylinder

$$\begin{aligned} Q_r^*(x, t) := B_r^*(x) \times (t-r^{2\alpha }, t]. \end{aligned}$$

We will omit the center of the cylinders whenever $(x, t)=(0,0).$ Moreover, we use the following convention to describe spacetime Hölder spaces: For $\alpha , \beta \in (0,1)$ and $Q \subset {\mathbb {R}}^2 \times {\mathbb {R}}$ we denote by $C^{\alpha , \beta }(Q)$ the functions which are $\alpha$- and $\beta$-Hölder continuous in space and time respectively, namely such that the following semi-norm is finite

$$\begin{aligned} \Vert \theta \Vert _{C^{\alpha , \beta }(Q)} = \sup \bigg \{\frac{|\theta (x,t)-\theta (y,s) |}{|x-y |^\alpha + |s-t |^\beta }:{ (x,t), (y,s) \in Q \text{ with } (x, t) \ne (y, s)} \bigg \}. \end{aligned}$$

Whenever $\alpha =\beta ,$ we denote the above space just by $C^\alpha (Q).$ Furthermore, we will also work with spatial Sobolev spaces of fractional order: For $\Omega \subseteq {\mathbb {R}}^n$, $s \in (0,1)$ and $1 \le p < \infty ,$ we denote by

$$\begin{aligned} W^{s, p}(\Omega ):=\left\{ f \in L^p(\Omega ): \frac{|f(x)-f(y) |}{|x-y |^{\frac{n}{p} +s} } \in L^p(\Omega \times \Omega )\right\} . \end{aligned}$$

Correspondingly, we define for $f \in W^{s,p}(\Omega )$ the Gagliardo semi-norm by

$$\begin{aligned}{}[f]_{W^{s,p}(\Omega )}:= \left( \int _{\Omega } \int _{\Omega } \frac{|f(x)-f(y) |^p}{|x-y |^{n +sp} } \, dx \, dy \right) ^\frac{1}{p}. \end{aligned}$$

In the special $p=2$, we will sometimes denote $W^{\alpha ,2}$ by $H^\alpha$ and we recall that for $\Omega ={\mathbb {R}}^n$ the Gagliardo semi-norm coïncides, up to a universal constant, with the semi-norms (9). Finally, we will consider the Bochner spaces $L^q((0, T), X)$ for $1 \le q \le \infty$ and for some Banach space *X* (here: $X=L^p({\mathbb {R}}^2)$ or $X=W^{\alpha , 2}({\mathbb {R}}^2)$). Whenever we work on a parabolic cube $Q_r(x,t)$, we will use the short-hand notation

$$\begin{aligned} L^2 W^{\alpha , 2}(Q_r):= L^q((t-r^{2\alpha }, t), W^{\alpha ,2}(B_r(x))). \end{aligned}$$

### Singular Points

We call a point $(x,t) \in {\mathbb {R}}^2 \times (0, \infty )$ a regular point of a Leray–Hopf weak solution $\theta$ of (1)–(3) (see Definition 3.1) if there exists a neighbourhood of (*x*, *t*) where $\theta$ is smooth. We denote by $\mathrm{Reg} \, \theta$ the open set of regular points in spacetime. Correspondingly, we define the spacetime singular set $\mathrm{Sing} \, \theta :=[ {\mathbb {R}}^2 \times (0, \infty )] \setminus \mathrm{Reg} \, \theta .$

### Riesz-Transform

We recall that the Riesz-transforms admit a singular integral representation. Indeed, for $f: {\mathbb {R}}^2 \rightarrow [0, \infty )$ and $i=1,2$

$$\begin{aligned} {\mathcal {R}}_if (x)= c \, \mathrm{p.v.} \int _{{\mathbb {R}}^2} \frac{x_i-z_i}{|x-z |^3} f(z) \, dz. \end{aligned}$$

By Calderon–Zygmund they are bounded operators on $L^p$ for $1<p< \infty$ and from $L^\infty$ to *BMO*.

### Caffarelli–Silvestre Extension

We recall the following extension problem. We use the notation ${\overline{\nabla }}$, ${\overline{\Delta }}$ for differential operators defined on the upper half-space ${\mathbb {R}}^{n+1}_+$.

#### Theorem 2.1

(Caffarelli–Silvestre [2]) Let $\theta \in H^{\alpha } ({\mathbb {R}}^n)$ with $\alpha \in (0,1)$ and set $b:=1-2\alpha$. Then there is a unique “extension” $\theta ^*$ of $\theta$ in the weighted space $H^1({\mathbb {R}}^{n+1}_+,y^b)$ which satisfies

$$\begin{aligned} \overline{\Delta }_b \theta ^*(x,y):={\overline{\Delta }} \theta ^*+\frac{b}{y}\partial _y \theta ^* = \frac{1}{y^b} \overline{\mathrm{div}}\, \big (y^b {\overline{\nabla }} \theta ^*\big )=0 \end{aligned}$$

and the boundary condition

$$\begin{aligned} \theta ^*(x,0)=\theta (x). \end{aligned}$$

Moreover, there exists a constant $c_{n, \alpha }$, depending only on *n* and $\alpha$, with the following properties:

- The fractional Laplacian $(-\Delta )^{\alpha } \theta$ is given by the formula
  $$\begin{aligned} (-\Delta )^{\alpha } \theta (x)=- c_{n,\alpha }\lim _{y\rightarrow 0}y^b\partial _y \theta ^* (x,y). \end{aligned}$$
- The following energy identity holds
  9 $$\begin{aligned} \int _{{\mathbb {R}}^n}|(-\Delta )^{\frac{\alpha }{2}} \theta |^2\,dx{=}\int _{{\mathbb {R}}^n}|\xi |^{2\alpha }|{\widehat{\theta }}(\xi )|^2\,d\xi {=} c_{n,\alpha }\int _{{\mathbb {R}}^{n+1}_+}y^b|{\overline{\nabla }} \theta ^*|^2\,dx\,dy.\quad \end{aligned}$$
- The following inequality holds for every extension $\eta \in H^1 ({\mathbb {R}}^{n+1}_+, y^b)$ of $\theta$:
  $$\begin{aligned} \int _{{\mathbb {R}}^{n+1}_+}y^b|{\overline{\nabla }} \theta ^*|^2\,dx\,dy \le \int _{{\mathbb {R}}^{n+1}_+}y^b|{\overline{\nabla }} \eta |^2\,dx\,dy. \end{aligned}$$

### Poincaré Inequalities

Let $\alpha \in (0, 1)$, $1\le p < \frac{n}{\alpha }$ and ${p^*} := \frac{pn}{n-p \alpha }$. There exists a universal constant $C=C(\alpha , n, p)$ such that for every $f \in W^{\alpha , p}({\mathbb {R}}^n)$, $q \in [p, p^*],$ $x \in {\mathbb {R}}^n$ and $r>0$

10 $$\begin{aligned} \left( \int _{B_{r}(x)} |f (z) - [f]_{B_{r}(x)} |^q \, dz \right) ^\frac{1}{q} \le C r^{\alpha - n (\frac{1}{p} - \frac{1}{q})} [f]_{W^{\alpha , p}(B_{r}(x))}. \end{aligned}$$

We will also need a weighted Poincaré inequality in the spirit of the classical work [17] for $\alpha =1$ (where on the other side much more general weights are admissible). Let $\omega \in C^\infty _{c}({\mathbb {R}}^n)$ be a radial, non-increasing weight such that $\omega \equiv 1$ on $\overline{B_{r/2}}(x)$, $\omega \equiv 0$ outside $B_{r}(x)\,$ and $|\nabla \omega |\le \frac{C}{r}$ pointwise. We introduce the weighted average

$$\begin{aligned}{}[f]_{\omega , B_{r}(x)}:= \bigg ( \int _{B_{r}(x)} \omega (z) \, dz \bigg )^{-1} \int _{B_{r}(x)} f(z) \omega (z) \, dz. \end{aligned}$$

The following weighted Poincaré inequality is classical for $\alpha =1$ (see [23, Lemma 6.12]) and it is established for $q=p$ in [16, Proposition 4]: Their proof extends to the other endpoint $q= p^*\,$ and hence to the range $q \in [p, p^*]$ by interpolation.

#### Lemma 2.2

Under the above assumptions, we have the weighted Poincaré inequality

11 $$\begin{aligned} \bigg (\int _{B_{r}(x)} |f(z) - [f]_{\omega , B_{r}(x)} |^q \omega (z) \, dz \bigg )^\frac{1}{q} \le {\tilde{C}} r^{\alpha - n (\frac{1}{p} - \frac{1}{q})} [f]_{W^{\alpha , p}(B_{r}(x))} \end{aligned}$$

where $${\tilde{C}}=2^{2-\alpha +n/p } C.$$

In the case $p=2$, we can rewrite the right-hand side of (10) and (11) in terms of the extension as follows.

#### Lemma 2.3

Let $n \ge 2$, $\alpha \in (0,1)\,$, $0<r<s,$ $f\in W^{\alpha , 2}({\mathbb {R}}^n)$ and $g \in C^1({\mathbb {R}})$. Then there exists $C=C(n, \alpha )$ such that

12 $$\begin{aligned}&[g \circ f]_{W^{\alpha , 2}(B_r)} \le C \bigg ( \int _{B_s^*} y^b |\overline{\nabla } [g(f^*)] |^2 \, dx \, dy\nonumber \\&\quad +(s-r)^{-2} \int _{B_s^*\setminus B_r^*} y^b( g(f^*) )^2 \, dx \, dy \bigg )^\frac{1}{2} \end{aligned}$$

and for any $2 \le q \le \frac{2n}{n-2\alpha }$

13 $$\begin{aligned}&\Vert g \circ f\Vert _{L^q(B_r)} \le C r^{-n \big (\frac{1}{2}-\frac{1}{q}\big )} \bigg (r^{2a}\int _{B_s^*} y^b |\overline{\nabla } [g(f^*)] |^2\, dx \, dy \nonumber \\&\quad +r^{2\alpha }(s-r)^{-2} \int _{B_s^*\setminus B_r^*} y^b( g(f^*) )^2 \, dx \, dy +\displaystyle \int _{B_r} (g(f))^2 \,dx\bigg )^\frac{1}{2}. \end{aligned}$$

In particular, for any $x \in {\mathbb {R}}^n\,$

14 $$\begin{aligned} {[}f]_{W^{\alpha ,2}(B_r(x))} \le C \bigg (\int _0^{\frac{4}{3} r} \int _{B_{\frac{4}{3} r}(x)} y^b |{\overline{\nabla }} f^* |^2 (z,y) \, dz \, dy \bigg )^\frac{1}{2}. \end{aligned}$$

#### Proof

Let $n \ge 2$, $\alpha \in (0,1)$, $0<r<s$ and $g \in C^1({\mathbb {R}})$. By approximation we may assume that *f* is Schwartz. Fix a smooth cut-off function $\varphi \in C^\infty _c({\mathbb {R}}^{n+1}_+)$ such that $0 \le \varphi \le 1$, $\varphi \equiv 1$ on $B_r^*$, ${{\,\mathrm{supp}\,}}\varphi \subseteq B_s^*$ and $|\overline{\nabla } \varphi |\le C (s-r)^{-1}.$ For $\alpha \in (0,1)$ we use the minimizing property of the extension to write

$$\begin{aligned} {[}g \circ f]_{W^{\alpha ,2}(B_r)}^2&= \int _{B_r} \int _{B_r} \frac{|g(f(x))- g(f(z)) |^2}{|x-z|^{n+2\alpha }} \, dx \, dz \\&\le \int _{{\mathbb {R}}^n}\int _{{\mathbb {R}}^n} \frac{|((g\circ f) \varphi |_{y=0}) (x) - ((g\circ f)\varphi |_{y=0})(z) |^2}{|x-z |^{n+2\alpha }} \,dx \, dz \\&= c_{n,\alpha } \int _{{\mathbb {R}}^{n+1}_+} y^b |\overline{\nabla }((g \circ f)\varphi |_{y=0})^*|^2 \, dx \, dy \\&\le c_{n,\alpha } \int _{{\mathbb {R}}^{n+1}_+} y^b |\overline{\nabla }(g(f^*)\varphi ) |^2 \, dx \, dy \\&\le 2c_{n,\alpha } \int _{{\mathbb {R}}^{n+1}_+} y^b |\overline{\nabla } [g(f^*)]|^2 \varphi ^2 \, dx \, dy\\&\quad + 2c_{n,\alpha } \int _{{\mathbb {R}}^{n+1}_+} y^b( g(f^*) )^2 |\overline{\nabla } \varphi |^2 \, dx \, dy \end{aligned}$$

and thus (12) follows. The estimate (13) follows from (12) via Sobolev embedding and interpolation. As for (14), we may assume $x=0$ and denoting by *c* the weighted average of $f^*$ with respect to the weight $y^b$ on $B_{4r/3} \times [0, 4r/3]$, we have by the weighted Poincaré inequality of [17] (with the Muckenhoupt weight $\omega (x,y)= y^b\in A_2$ on ${\mathbb {R}}^3_+$)

15 $$\begin{aligned} r^{-2} \int _0^{\frac{4}{3}r}\int _{B_{\frac{4}{3}r}} y^b |f^* - c |^2 \, dx \, dy \lesssim \int _0^{\frac{4}{3}r}\int _{B_{\frac{4}{3}r}} y^b |\overline{\nabla } f^* |^2 \, dx \, dy. \end{aligned}$$

The estimate (14) follows then from (12) (applied to $f-c$ and $s=4r/3$) and (15). $\square$

### (Sharp) Maximal Function

For a function $f: {\mathbb {R}}^2 \times [0, \infty ) \rightarrow {\mathbb {R}}\,$ we introduce the maximal function (in space)

$$\begin{aligned} {\mathcal {M}}f(x,t) := \sup _{r >0} \, \fint _{B_r(x)} |f(z, t) |\, dz \end{aligned}$$

as well as the sharp fractional maximal function (in space)

$$\begin{aligned} f^\#_{\alpha } (x,t):= \sup _{r>0} r^{-\alpha } \fint _{B_r(x)} |f(z,t) - [f(t)]_{B_r(x)} |\, dz \, \end{aligned}$$

and for $q> \sqrt{2}$ the following variant

16 $$\begin{aligned} f^\#_{\alpha , q} (x,t):= \sup _{r>0} r^{-2\alpha (1- 1/q^2)} \fint _{B_r(x)} |f(z,t) - [f(t)]_{B_r(x)} |^{2(1-1/q^2)} \, dz. \end{aligned}$$

In order to use a spacetime integral of $\theta ^\#_{\alpha , q}$ in a covering argument, we need to know that it is globally controlled; to guarantee the latter, we are forced to choose $q< \infty$.

#### Lemma 2.4

Let $\alpha \in (0,1),$ $f \in W^{\alpha , 2}({\mathbb {R}}^n) \,$ and $q \in (\sqrt{2}, \infty ).$ Then there exists a constant $C=C(n,q) \ge 1$ (which is uniformly bounded for *q* bounded away from $\infty$) such that

$$\begin{aligned} \Vert f^\#_{\alpha }\Vert _{L^2({\mathbb {R}}^n)}^2 + \Vert f^{\#}_{\alpha , q} \Vert _{L^{1+1/(q^2-1)}({\mathbb {R}}^n)}^{1+1/(q^2-1)} \le C [f]_{W^{\alpha ,2}({\mathbb {R}}^n)}^2. \end{aligned}$$

For $\alpha =1,$ the equivalent of Lemma 2.4 is a simple consequence of the Poincaré inequality and the maximal function estimate. Indeed, by Poincaré we have almost everywhere the pointwise estimate

$$\begin{aligned} f^\#_1(x) \lesssim {\mathcal {M}}(|\nabla f |)(x) \qquad f^\#_{1,q}(x) \lesssim {\mathcal {M}}\big (|\nabla f |^{2(1-1/q^2)}\big )(x) \end{aligned}$$

for $f \in W^{1,1}_{loc}$ and $f \in W^{1,2 (1-1/q^2) }_{loc}$ respectively. Integrating in *x* and using the boundedness of the maximal function on $L^2$ and $L^{1+1/(q^2 -1)},$ we obtain the equivalent of Lemma 2.4.

#### Proof

We give the proof for $f^\#_{\alpha ,q}$. We estimate the quantity in the supremum in (16)

17 $$\begin{aligned} \fint _{B_r(x)} \bigg |\, \fint _{B_r(x)} \frac{f(z) - f(y)}{r^{\alpha }} \, dy \bigg |^{2(1-1/q^2)} \, dz&\le \fint _{B_r(x)} \bigg (\, \fint _{B_r(x)} \frac{|f(z) - f(y)|^2}{r^{2\alpha }} \, dy \bigg )^{1-1/q^2} \, dz \nonumber \\&\le C \fint _{B_r(x)} \bigg ( \, \int _{B_r(x)} \frac{|f(z) - f(y) |^2}{|z-y |^{n+2\alpha }}\, dy \bigg )^{1-1/q^2} \, dz \nonumber \\&\le C \, \fint _{B_r(x)} \left( D_{\alpha , 2} \, f(z) \right) ^{2(1-1/q^2)} \, dz, \end{aligned}$$

where $D_{\alpha , 2} \, f$ is the *n*-dimensional version of (6), i.e. for $z \in {\mathbb {R}}^n$

$$\begin{aligned} (D_{\alpha , 2} \, f) (z):=\left( \int _{{\mathbb {R}}^n} \frac{|f(z)-f(z') |^2}{|z-z' |^{n+2\alpha }} \, dz' \right) ^{\frac{1}{2}}. \end{aligned}$$

By taking the supremum over $r>0,$ we deduce from (17) that for almost every *x*

18 $$\begin{aligned} f^\#_{\alpha , q} (x) \le C {\mathcal {M}}\big ((D_{\alpha ,2} \, f)^{2(1-1/q^2)}\big )(x) \end{aligned}$$

and hence by the maximal function estimate on $L^{1 + {1}/{(q^2-1)}}$

$$\begin{aligned} \Vert f^\#_{\alpha , q} \Vert ^{1+1/(q^2-1)}_{L^{1 + {1}/{(q^2-1)}}({\mathbb {R}}^n)}\le & {} C \Vert (D_{\alpha ,2} \, f)^{2(1-1/q^2)}\Vert ^{1+1/(q^2-1)}_{L^{1 + {1}/{(q^2-1)}}({\mathbb {R}}^n)} \\= & {} C \Vert D_{\alpha ,2}\, f\Vert ^2_{L^{2}({\mathbb {R}}^n)} = C[f]^2_{W^{\alpha ,2}({\mathbb {R}}^n)}. \end{aligned}$$

$$\square $$

## The Local Energy Inequalities

### Leray–Hopf Weak Solutions

We recall the notion of Leray–Hopf weak solutions.

#### Definition 3.1

Let $\theta _0\in L^2 ({\mathbb {R}}^2)$. A pair $(\theta ,u)$ is a Leray–Hopf weak solution of (1)–(2) on ${\mathbb {R}}^2\times (0,T)$ if:

- $$\theta \in L^\infty ((0,T), L^2 ({\mathbb {R}}^2)) \cap L^2 ((0,T), W^{\alpha ,2} ({\mathbb {R}}^2))$$;
- $\theta$ solves (1)–(2) in the sense of distributions, namely ${{\,\mathrm{div}\,}}u= 0$ and
  19 $$\begin{aligned} \int \Big (\partial _t \varphi \theta + u \theta \cdot \nabla \phi - (-\Delta )^\alpha \varphi \theta \Big )\, dx\, dt = - \int \theta _0 (x) \cdot \varphi (0,x)\, dx \end{aligned}$$
  for any $$\varphi \in C^\infty _c ({\mathbb {R}}^2\times {\mathbb {R}})$$.
- The following inequalities hold for every $t\in (0,T)$ and for almost every $s\in (0,T)$ and every $t \in (s,T)$ respectively:
  20 $$\begin{aligned} \frac{1}{2} \int \theta ^2 (x,t)\, dx + \int _0^t \int |(-\Delta )^{\frac{\alpha }{2}} \theta |^2 (x,\tau )\, dx\, d\tau&\le \frac{1}{2} \int |\theta _0|^2 (x)\, dx \end{aligned}$$
  21 $$\begin{aligned} \frac{1}{2} \int |\theta |^2 (x,t)\, dx + \int _s^t \int |(-\Delta )^{\frac{\alpha }{2}} \theta |^2 (x,\tau )\, dx\, d\tau&\le \frac{1}{2} \int |\theta |^2 (x,s)\, dx \end{aligned}$$

Correspondingly, we say that $\theta$ is a Leray–Hopf weak solution of (1)–(3) if additionally (3) holds.

Observe that from the weak formulation (19) it follows that for all $\varphi \in C^\infty _c({\mathbb {R}}^3_+ \times [0, T))$

22 $$\begin{aligned}&\int \theta (x,t) \varphi (x,0,t) \, dx - \int \theta (x,s) \varphi (x,0,s) \, dx \nonumber \\&\quad = \int _s^t \int \Big ( \theta \partial _\tau \varphi |_{y=0} + (u\theta ) \cdot \nabla \varphi |_{y=0} - \theta (-\Delta )^\alpha \varphi |_{y=0} \Big ) \, dx \, d\tau \nonumber \\&\quad = \int _s^t \int \Big ( \theta \partial _\tau \varphi |_{y=0} + (u\theta ) \cdot \nabla \varphi |_{y=0} \Big ) \, dx \, d \tau - c_{\alpha } \int _s^t \int _{{\mathbb {R}}^3_+} y^b \overline{\nabla } \theta ^* \cdot \overline{\nabla } \varphi \, dx \, dy \, d\tau \, \end{aligned}$$

for $s=0$ and almost every $t\in (0,T)$ and for almost every $0<s<t<T\,$ (with $c_\alpha$ given by Theorem 2.1). Indeed, the equality between the last term of the second line and the last term of (22) holds for every $\theta \in L^2((0,T),W^{\alpha ,2}({\mathbb {R}}^2))$; in the smooth case this equality is a consequence of Theorem 2.1 which one recovers for general $\theta$ through regularization.

We recall that any Leray–Hopf weak solution is actually in $L^\infty$ for $t>0$.

#### Theorem 3.2

([10] Theorem 2.1) Let $\theta _0 \in L^2({\mathbb {R}}^2)$ and let $(\theta , u)$ be a Leray–Hopf weak solution of (1)–(2). Then there exist a universal constant, independent on *u*, such that for any $t>0\,$

23 $$\begin{aligned} \sup _{x \in {\mathbb {R}}^2} |\theta (x,t) |\le C\Vert \theta _0\Vert _{L^2} t^{-\frac{1}{2\alpha }}. \end{aligned}$$

In the particular case (3), where $u= {\mathcal {R}}^\perp \theta$, we obtain as a consequence that for any $t>0$

24 $$\begin{aligned} \Vert u(\cdot , t)\Vert _{{BMO}({\mathbb {R}}^2)} \le C \Vert \theta _0\Vert _{L^2}t^{-\frac{1}{2\alpha }}. \end{aligned}$$

#### Remark 3.3

In [10] Theorem 3.2 is proven for Leray–Hopf weak solutions of the coupled system (1)–(3). However, in the proof of (23) only the energy inequality on level sets together with the assumption ${{\,\mathrm{div}\,}}u=0$ is used; the structure (3) is only used to deduce (24) from (23).

### Suitable Weak Solutions

We are now ready to give our definition of suitable weak solution. Both this notion and the one of Leray–Hopf solution are given without requiring the coupling (3), since, in the proof of Theorem 1.3, we will need to work on a larger class of equations, where *u* is obtained from $\theta$ by means of the Riesz transform and a temporal translation.

#### Definition 3.4

A Leray–Hopf weak solution $(\theta ,u)$ of (1)–(2) on ${\mathbb {R}}^2 \times (0,T)$ is a suitable weak solution if the following two inequalities hold for almost every $t\in (0,T)$, all nonnegative test functions^1^$\varphi \in C^{\infty }_c ( {\mathbb {R}}^3_+ \times (0,T))$ with $\partial _y \varphi (\cdot ,0,\cdot )= 0$ in ${\mathbb {R}}^2 \times (0,T)$, for all $q \ge 2$ and every linear transformation of the form $\eta := (\theta -M)/L$ with scalar $L>0$ and shift $M \in {\mathbb {R}}$:

25 $$\begin{aligned}&\int _{{\mathbb {R}}^2} \varphi (x,0,t) \eta ^2 (x,t) \, dx +2 c_{\alpha }\int _0^t \int _{{\mathbb {R}}^3_+} y^b|\overline{ \nabla } \eta ^* |^2 \varphi \, dx \, dy \, ds\nonumber \\&\quad \le \; \int _0^t \int _{{\mathbb {R}}^2}( \eta ^2 \partial _t \varphi |_{y=0} + u \eta ^2 \cdot \nabla \varphi |_{y=0})\, dx \, ds + c_{\alpha } \int _0^t\int _{{\mathbb {R}}^3_+} y^b (\eta ^*)^2 {\overline{\Delta }}_b \varphi \, dx \, dy \, ds, \end{aligned}$$

26 $$\begin{aligned}&\int _{{\mathbb {R}}^2} \varphi (x,0,t) |\eta |^q(x,t) \, dx +4 \big (1- \tfrac{1}{q}\big ) c_{\alpha }\int _0^t \int _{{\mathbb {R}}^3_+} y^b|\overline{ \nabla } |\eta ^* |^\frac{q}{2} |^2 \varphi \, dx \, dy \, ds\nonumber \\&\quad \le \; \int _0^t \int _{{\mathbb {R}}^2}( |\eta |^q \partial _t \varphi |_{y=0} + u |\eta |^q \cdot \nabla \varphi |_{y=0})\, dx \, ds + c_{\alpha } \int _0^t\int _{{\mathbb {R}}^3_+} y^b |\eta ^*|^q {\overline{\Delta }}_b \varphi \, dx \, dy \, ds, \end{aligned}$$

where the constant $c_{\alpha }$ depends only on $\alpha$ and comes from Theorem 2.1.

Correspondingly, we say that $\theta$ is a suitable weak solution of (1)–(3) if additionally (3) holds.

#### Remark 3.5

In the classical notion of suitable weak solutions for the (hyperdissipative) Navier-Stokes equations, the local energy inequality (25) is asked to hold only for $\theta$ and not for every linear transformation $\eta :=(\theta -M)/L$. However, it can be proved (see for instance [7]) that the class of suitable weak solutions is stable under this transformation. Here on the other hand, since we use a “nonlinear” energy inequality (26), it is no longer obvious that the class of suitable weak solutions is stable under linear transformations; hence we require it already in the definition. The class of suitable weak solutions contains smooth solutions (see Sect. 3.3) and is non-empty (see Sect. 3.4) for any $L^2$ initial datum.

### Local Energy Equality for Smooth Solutions

It is not difficult to see that (25) and (26) hold with an equality for every *smooth* solution of (1)–(2). Indeed, let $f\in C^2({\mathbb {R}})$. We multiply (1) by $f'(\theta ) \varphi |_{y=0}$ and integrate in space to obtain for $t\in [0,T]$

$$\begin{aligned}&\int _{{\mathbb {R}}^2} f(\theta ) (x,t) \varphi |_{y=0}(x, t) \, dx - \int _0^t \int _{{\mathbb {R}}^2} [f(\theta ) \partial _t \varphi |_{y=0} + u f(\theta ) \cdot \nabla \varphi |_{y=0}] \, dx \, ds\\&\quad = -\int _0^t \int _{{\mathbb {R}}^2} (-\Delta )^\alpha \theta f'(\theta )\varphi |_{y=0} \, dx \, ds. \end{aligned}$$

By means of the divergence theorem, we compute for fixed time *t*

$$\begin{aligned}&\int _{{\mathbb {R}}^2} (-\Delta )^\alpha \theta (x,t) f'(\theta )(x,t) \varphi |_{y=0}(x,t) \, dx\\&\quad =-c_\alpha \lim _{y \rightarrow 0^+} \int _{{\mathbb {R}}^2} y^b \partial _y \theta ^*(x,y,t) \left( f'(\theta ^*) \varphi \right) (x,y,t) \, dx\\&\quad = c_\alpha \int _{{\mathbb {R}}^3_+} \overline{{{\,\mathrm{div}\,}}} ( y^b \overline{\nabla } \theta ^* f'(\theta ^*)\varphi ) \, dx \, dy\\&\quad = c_\alpha \int _{{\mathbb {R}}^3_+} y^b |\overline{\nabla }\theta ^* |^2 f''(\theta ^*) \varphi \, dx \, dy - {c_\alpha }\int _{{\mathbb {R}}^3_+} y^b f(\theta ^*)\overline{\Delta }_b \varphi \, dx \, dy, \end{aligned}$$

where we integrated by parts in the third equality and used that the boundary terms vanish due to the hypothesis $\partial _y \varphi (\cdot , 0, \cdot ) = 0.$ We obtain that for $f\in C^2({\mathbb {R}})$

$$\begin{aligned}&\int _{{\mathbb {R}}^2} f(\theta )(x,t) \varphi |_{y=0} (x,t) \, dx + c_\alpha \int _0^t \int _{{\mathbb {R}}^3_+} y^b |\overline{\nabla }\theta ^* |^2 f''(\theta ^*) \varphi \, dx \, dy \, ds\\&\quad = \int _0^t \int _{{\mathbb {R}}^2} \left[ f(\theta ) \partial _t \varphi |_{y=0} + u f(\theta ) \cdot \nabla \varphi _{y=0} \right] \, dx \, ds + c_\alpha \int _0^t \int _{{\mathbb {R}}^3_+} y^b f(\theta ^*) \overline{\Delta }_b \varphi \, dx \, dy \, ds. \end{aligned}$$

Observe that if *f* is moreover convex and nonnegative, both the left- and the right-hand side of the above equality have a sign. In particular, we obtain (25) with an equality when choosing $f(x)=\big (\frac{x-M}{L}\big )^2$ (since $f''\equiv 2L^{-2}$) and (26) when choosing $f(x)= \big |\frac{x-M}{L} \big |^{q}$ for $q \ge 2.$

### Existence of Suitable Weak Solutions

For any $\alpha \in (0, \frac{1}{2})$ the existence of suitable weak solutions can be established from any initial datum $\theta _0 \in L^2({\mathbb {R}}^2)$ by adding a vanishing viscosity term $\epsilon \Delta \theta$ on the right-hand side and letting $\epsilon \rightarrow 0.$ The key argument is a classical Aubin-Lions type compactness argument that we sketch in Appendix C.

#### Theorem 3.6

For any $\theta _0\in L^2 ({\mathbb {R}}^2)$ there is a suitable weak solution of (1)–(3) on ${\mathbb {R}}^2 \times (0, \infty )$.

### Compactness

We establish the compactness of a sequence of suitable weak solutions with vanishing excess. Let $(\theta , u)$ be a solution of (1)–(2) on ${\mathbb {R}}^2 \times (0, T).$ For $r>0$ and $Q_r(x,t) \subseteq {\mathbb {R}}^2 \times (0, T)$, we define the excess as

$$\begin{aligned} E(\theta , u; x, t, r)&:= E^S(\theta ; x,t,r) + E^V(u; x, t,r)+ E^{NL} (\theta ; x, t,r) \end{aligned}$$

where

$$\begin{aligned} E^S(\theta ; x,t,r)&:= \bigg (\,\fint _{Q_r(x,t)} |\theta (z,s)- (\theta )_{Q_r(x,t)} |^p \, dz \, ds \bigg )^\frac{1}{p}\\ E^V(u; x, t,r)&:= \bigg (\, \fint _{Q_r(x,t)} |u(z,s)- \left[ u(s)\right] _{B_r(x)} |^p \, dz \, ds \bigg )^\frac{1}{p}\\ E^{NL}(\theta ; x, t, r)&:= \bigg (\,\fint _{t-r^{2\alpha }}^t \sup _{R \ge \frac{r}{4}} \left( \frac{r}{R} \right) ^{\sigma p} \bigg (\, \fint _{B_R(x)} |\theta (z,s) - [\theta (s)]_{B_r(x)}|^{ \frac{3}{2}} \, dz \bigg )^\frac{2p}{3} \, ds \bigg )^\frac{1}{p}, \end{aligned}$$

for $p \in (3, \infty )$ and $\sigma \in (0, 2\alpha )$ yet to be chosen. Observe that both parameters serve as (hidden) parameter for now and will be chosen in the very end to close the main $\varepsilon$-regularity Theorem (see also Remark 5.4). Whenever $(x,t)=(0,0)$, we will denote the excess simply by $E(\theta , u;r).$

#### Remark 3.7

(Rescaling of the excess) The excess behaves nicely under the natural rescaling (4). Indeed, for $r>0$ we have

$$\begin{aligned} E(\theta , u;x,t,r)= r^{1-2\alpha } E(\theta _r, u_r ;x, t, 1). \end{aligned}$$

#### Lemma 3.8

(Compactness) Let $\alpha \in (0, \frac{1}{2}]\,,$ $\sigma \in (0, 2\alpha )$ and $p> \frac{1+\alpha }{\alpha }.$ Let $(\theta _k, u_k)$ be a sequence of suitable weak solutions of (1)–(2) on ${\mathbb {R}}^2 \times [-1,0]\,$ with

- $$\lim _{k \rightarrow \infty }E(\theta _k, u_k ;1) =0\,$$
- and $[u_k(s)]_{B_1} =0$ for all $s \in [-1, 0].$

Set $E_k:=E(\theta _k, u_k; 1)$ and define $\eta _k:=(\theta _k - (\theta _k)_{Q_1})/E_k.$

Then there exists $\eta \in L^{3/2}_{loc}({\mathbb {R}}^2 \times [-1, 0])$ such that, up to subsequences, $\eta _k \rightharpoonup \eta$ weakly in $L^{3/2}_{loc}({\mathbb {R}}^2 \times [-1,0]).$ Moreover,

$$\begin{aligned} \eta _k \rightarrow \eta \text { strongly in }L^p(Q_{3/4})\, \end{aligned}$$

and $$\eta $$ solves

$$\begin{aligned} \partial _t \eta + (-\Delta )^\alpha \eta =0 \text { on } Q_{3/4}\, \end{aligned}$$

with $$E^S(\eta ;1) +E^{NL}(\eta ;1) \le 1.$$

We will need the following auxiliary Lemma.

#### Lemma 3.9

(Tail estimate) Let $\alpha \in (0, \frac{1}{2}]\,,$ $\sigma \in (0, 2 \alpha )$ and $1<p<\infty \,$. Then there exists a universal constant $C=C(\alpha , \sigma , p)\ge 1$ such that for every $\theta \in L^p(B_2)$ with

$$\begin{aligned} \sup _{R \ge 1} \frac{1}{R^{\sigma p}} \bigg (\, \fint _{B_R} |\theta (x) |\, dx \bigg )^p < + \infty , \end{aligned}$$

we have the estimate

$$\begin{aligned} \int _{B_1^*} y^b |\theta ^*(x,y) |^p \, dx \, dy \le C \Bigg ( \int _{B_2} |\theta (x) |^p \, dx +\sup _{R \ge 1} \frac{1}{R^{\sigma p}} \bigg ( \,\fint _{B_R} |\theta (x) |\, dx \bigg )^p\Bigg ). \end{aligned}$$

#### Proof

We set $\theta _1:= \theta \mathbb {1}_{B_2}$ and $\theta _{i+1} := \theta (\mathbb {1}_{B_{2^{i+1}}}- \mathbb {1}_{B_{2^i}})$ for $i\ge 1\,$. Recall that for $y>0$ the extension is, up to a normalizing dimensional constant, given by $\theta ^*(x,y)= (P(\cdot , y) *\theta )(x)$ for $P(x,y)= y^{2\alpha }/(|x |^2 + y^2)^{1+\alpha }.$ We estimate

$$\begin{aligned} \int _{B_1^* } y^b |\theta ^* |^p\, dx \, dy \lesssim \int _{B_1^*} y^b |\theta _1^* |^p \, dx \, dy+ \int _0^1 y^b \Big ( \sum _{i>1} \Vert \theta _i^*(\cdot , y)\Vert _{L^\infty (B_1)} \Big )^p \, dy. \end{aligned}$$

The first term is estimated using Young and the fact that $\Vert P(\cdot , y)\Vert _{L^1({\mathbb {R}}^2)}=\Vert P(\cdot , 1)\Vert _{L^1({\mathbb {R}}^2)} = C_{\alpha }$ is a universal constant (see for instance the appendix of [7]). Indeed,

$$\begin{aligned} \int _{B_1^*} y^b |\theta _1^* |^p \, dx \, dy&= \int _0^1 y^b \Vert \theta _1 *P(\cdot , y)\Vert _{L^p(B_1)}^p \, dy \\&\le \int _0^1 y^b \Vert P(\cdot , y)\Vert _{L^1({\mathbb {R}}^2)}^p \Vert \theta _1\Vert _{L^p({\mathbb {R}}^2)}^p \, dy \le C_{\alpha }^p \int _{B_2} |\theta |^p \, dx. \end{aligned}$$

For $i\ge 1$, we estimate, using the fact that for $x \in B_1$ and $z \in B_{2^{i+1}} \setminus B_{2^i}$ we have $|x-z |\ge 2^{i-1}$ and thus $P(x-z,y) \le P(2^{i-1}, y)$ uniformly in *z*,

$$\begin{aligned} \Vert \theta _{i+1}^*(\cdot , y)\Vert _{L^\infty (B_1)} \le P(2^{i-1}, y) \int _{B_{2^{i+1}} \setminus B_{2^i}} |\theta |\, dx \le \int _{B_{2^{i+1}} \setminus B_{2^i}} \frac{y^{2\alpha } |\theta |}{2^{2(1+\alpha )(i-1)}} \, dx, \end{aligned}$$

so that

$$\begin{aligned} \sum _{i>1} \Vert \theta _i^* (\cdot , y)\Vert _{L^\infty (B_1)}&\le \sum _{i\ge 1} \frac{y^{2\alpha }}{2^{(2\alpha -\sigma )(i-1)}} \int _{B_{2^{i+1}} \setminus B_{2^i}} \frac{|\theta |}{2^{(2+\sigma )(i-1)}} \, dx \\&\le C y^{2\alpha } \Big ( \sum _{i\ge 1} \frac{1}{2^{(2\alpha -\sigma )(i-1)}} \Big ) \sup _{R \ge 1} \frac{1}{R^\sigma } \fint _{B_R} |\theta |\,dx. \end{aligned}$$

We obtain the claim by raising the previous inequality to the power *p*. $\square$

#### Proof of Lemma 3.8

Observe that $u_k \rightarrow 0$ in $L^p(Q_1)$ and thus, we may assume that

$$\begin{aligned} \sup _{k \ge 1} \, \Vert u_k\Vert _{L^p(Q_1)} \le 1. \end{aligned}$$

Moreover, by construction the pair $(\eta _k, u_k)$ is a distributional solution to

$$\begin{aligned} \partial _t \eta _k + u_k \cdot \nabla \eta _k + (-\Delta )^\alpha \eta _k =0\, \end{aligned}$$

with $$E(\eta _k, \frac{u_k}{E_k}; 1) = 1.$$

*Step 1: We prove the uniform boundedness of* $\eta _k$ *in* $\big (L^\infty L^2 \cap L^2 W^{\alpha ,2} \cap L^{(p-1)(1+\alpha )}\big )(Q_{3/4}).$

Fix a test function $\varphi \in C^\infty _c({\mathbb {R}}^3_+ \times (0, \infty ))$ such that $0 \le \varphi \le 1$, ${{\,\mathrm{supp}\,}}\varphi \subset Q_{7/8}^*$ and $\varphi \equiv 1$ on $Q_{27/32}^*.$ Moreover, we assume that $\varphi$ is constant in *y* for small *y*, that is $\partial _y \varphi =0$ for $\{y < \frac{1}{2}\}.$ From the local energy inequality (25) we deduce that for $t \in [-(13/16)^{2\alpha }, 0]$

$$\begin{aligned}&\int _{B_{27/32}} \eta _k^2(x,t) \, dx + 2 c_\alpha \int _{-\left( \frac{13}{16}\right) ^{2\alpha }}^t \int _{B_{27/32}^*} y^b |{\overline{\nabla }} \eta _k^*|^2(x,y,s) \, dx \, dy \, ds \\&\quad \le \int _{B_{7/8}} \eta _k^2(x,t) \varphi (x,0,t) \, dx + 2 c_\alpha \int _{-\left( \frac{7}{8}\right) ^{2\alpha }}^t \int _{B_{7/8}^*} y^b |{\overline{\nabla }} \eta _k^*|^2(x,y,s) \varphi (x,y,s) \, dx \, dy \, ds\\&\quad \le \int _{-\left( \frac{7}{8}\right) ^{2\alpha }}^t \int _{B_{7/8}} ( \eta _k^2 \partial _t \varphi |_{y=0}+ u_k \eta _k^2 \cdot \nabla \varphi |_{y=0})\, dx \, ds\\&\qquad + c_\alpha \int _{-\left( \frac{7}{8}\right) ^{2\alpha }}^t \int _{B_{7/8}^*} y^b (\eta _k^*)^2 \overline{\Delta }_b \varphi \,dx \, dy \, ds \\&\quad \lesssim \int _{Q_{7/8}}( \eta _k^2+ |\eta _k |^\frac{2p}{p-1}+ |u_k |^p) \, dx \, ds + \int _{Q_{7/8}^*} y^b ( \eta _k^*)^2 \, dx \, dy \, ds. \end{aligned}$$

Using Lemma 2.3, the previous inequality and Lemma 3.9, we deduce that for $t \in [-(13/16)^{2\alpha }, 0]$

$$\begin{aligned}&\int _{B_{13/16}} \eta _k^2(x,t) \, dx + \int _{-\left( \frac{13}{16}\right) ^{2\alpha }}^t [\eta _k(s)]_{W^{\alpha , 2}(B_{13/16})}^2 \, ds\\&\quad \lesssim \int _{B_{27/32}} \eta _k^2(x,t) \, dx + 2 c_\alpha \int _{-\left( \frac{13}{16}\right) ^{2\alpha }}^t \int _{B_{27/32}^*} y^b\big ( |\overline{\nabla }\eta _k^* |^2 +(\eta _k^*)^2\big ) \, dx \, dy \, ds \\&\quad \lesssim \int _{Q_1}( \eta _k^2+ |\eta _k |^\frac{2p}{p-1}+ |u_k |^p )\, dx \, ds \\&\qquad + \int _{-1}^0 \sup _{R \ge 1} \frac{1}{R^{2\sigma }} \bigg (\, \fint _{B_R} |\eta _k(x,s) - [\eta _k(s)]_{B_1} |\, dx \bigg )^2\, ds\\&\quad \lesssim 1, \end{aligned}$$

where we used in the last inequality that $\frac{2p}{p-1} \le p \,$ together with the fact $E(\eta _k, \frac{u_k}{E_k};1) =1.$ Taking the supremum over $t \in [-(13/16)^{2\alpha }, 0]\,,$ we deduce that uniformly in $k \ge 1$

$$\begin{aligned} \sup _{t \in [-(13/16)^{2\alpha }, 0]} \int _{B_{13/16}} \eta _k^2(x,t) \, dx&+ \int _{-\left( \frac{13}{16}\right) ^{2\alpha }}^0 [\eta _k(s)]_{W^{\alpha ,2}(B_{13/16})}^2 \, ds \le C. \end{aligned}$$

We now consider $\psi _k := |\eta _k |^\frac{p-1}{2} \,$. Using Lemma 2.3 applied with $r=\frac{13}{16}$, $s= \frac{27}{32}$, $g(x)= |x|^{\frac{p-1}{2}}$, the local energy inequality (26) for $\psi _k$ and proceeding as before, using also that $p>3$, we thus have for any $t \in [-(13/16)^{2\alpha }, 0]$ that

$$\begin{aligned}&\int _{B_{13/16}} \psi _k^2(x,t) \, dx + \int _{-\left( \frac{13}{16}\right) ^{2\alpha }}^t [\psi _k(s)]_{W^{\alpha ,2}(B_{13/16})} \, ds \\&\quad \lesssim \int _{B_{27/32}} |\eta _k |^{p-1}(x,t) \, dx + \int _{-\left( \frac{27}{32}\right) ^{2\alpha }}^t \int _{B_{27/32}^*} y^b { |{\overline{\nabla }}} |\eta _k^* |^\frac{p-1}{2} |^2 \, dx \, dy \, ds\\&\qquad +\int _{Q_{27/32}^*} y^b |\eta _k^*|^{p-1} \, dx \, dy \, ds \\&\quad \lesssim \int _{Q_{7/8}}\big ( |\eta _k |^{p-1}+ |\eta _k |^p+ |u_k |^p \big ) \, dx \, ds\\&\qquad +\int _{Q_{7/8}^*} y^b |\eta _k^*|^{p-1} \, dx \, dy \, ds \\&\quad \lesssim \int _{Q_1}\big ( |\eta _k |^{p-1}+ |\eta _k |^p+ |u_k |^p \big ) \, dx \, ds \\&\qquad + \int _{-1}^0 \sup _{R \ge 1} \frac{1}{R^{(p-1)\sigma }} \bigg (\,\fint _{B_R} |\eta _k(x,s) - [\eta _k(s)] |\, dx \bigg )^{p-1}\, ds \\&\quad \lesssim 1. \end{aligned}$$

Taking the supremum over $t \in [-(13/16)^{2\alpha }, 0]$, we obtain as before the uniform-in-*k* bound

$$\begin{aligned} \sup _{t \in [-(13/16)^{2\alpha }, 0]} \int _{B_{13/16}} \psi _k^2(x,t) \, dx + \int _{-\left( \frac{13}{16}\right) ^{2\alpha }}^0 [\psi _k(s)]_{W^{\alpha ,2}(B_{13/16})}^2 \, ds \le C. \end{aligned}$$

From Sobolev embedding $\dot{W}^{\alpha , 2} \hookrightarrow L^\frac{2}{1-\alpha }\,,$ we obtain by interpolation that $\Vert \psi _k\Vert _{L^{2(1+\alpha )}(Q_{13/16})} \le C \,$ uniformly in $k\ge 1$ and hence in particular $\sup _{k \ge 1} \Vert \eta _k\Vert _{L^{(p-1)(1+\alpha )}(Q_{13/16})} \le C.$

*Step 2: We use an Aubin-Lions type compactness argument to deduce strong convergence of* $\eta _k$ *in* $L^q(Q_{3/4})$ *for every* $1 \le q< (p-1)(1+\alpha ).$ *Since* $(p-1)(1+\alpha ) >p$ *by hypothesis, we deduce in particular that* $\eta _ k \rightarrow \eta$ *strongly in* $L^p(Q_{3/4}).$

We may assume $q \in [2, (p-1)(1+\alpha ))$. Since the excess uniformly bounds the $L^{3/2}_{loc}$-norm of $\eta _k$, there exists by Banach-Alaoglu a limit $\eta \in L^{3/2}_{loc}({\mathbb {R}}^2 \times [-1,0])$ such that $\eta _k \rightharpoonup \eta$ weakly in $L^{3/2}_{loc}({\mathbb {R}}^2\times [-1,0])$, up to extracting a subsequence. By the uniform boundedness established in Step 1, we may assume, up to extracting a further subsequence, that $\eta _k \rightharpoonup \eta$ weakly in $L^q(Q_{13/16})$. We now claim that the latter convergence is in fact strong on the slightly smaller cube $Q_{3/4}$. Indeed, fix $\varepsilon >0$ and a family $\{\phi _\delta \}_{\delta >0}$ of mollifiers in the space variable. For $k, j\ge 1$ we estimate

$$\begin{aligned}&\Vert \eta _k-\eta _j\Vert _{L^q(Q_{3/4})} \le \Vert \eta _k-\eta _k *\phi _\delta \Vert _{L^q(Q_{3/4})}\\&\quad + \Vert \eta _j-\eta _j *\phi _\delta \Vert _{L^q(Q_{3/4})} + \Vert (\eta _k-\eta _j) *\phi _\delta \Vert _{L^q(Q_{3/4})}. \end{aligned}$$

We claim that the first two contributions converge to 0 as $\delta \rightarrow 0$, uniformly in *k* and *j*. Indeed, we compute for $\delta$ small enough by Hölder and the uniform boundedness of $\eta _k$ in $L^2 W^{\alpha ,2}(Q_{13/16})$

27 $$\begin{aligned}&\Vert \eta _k-\eta _k *\phi _\delta \Vert _{L^2(Q_{3/4})}^2 = \int _{-\left( \frac{3}{4}\right) ^{2\alpha }}^0 \int _{B_{3/4}} \bigg |\int (\eta _k(x)-\eta _k(y)) \phi _\delta (x-y) \, dy \bigg |^2 \, dx \, dt\nonumber \\&\quad \le \int _{-\left( \frac{3}{4}\right) ^{2\alpha }}^0 \int _{B_{3/4}} \bigg ( \int \frac{|\eta _k(x)-\eta _k(y) |^2}{|x-y |^{2+2\alpha }} \mathbb {1}_{|x-y |\le \delta } \, dy\bigg ) \nonumber \\&\qquad \bigg ( \int \phi _\delta ^2(x-y) |x-y |^{2+2\alpha } \, dy \bigg ) \, dx \,dt\nonumber \\&\quad \le \pi \delta ^{2\alpha } \Vert \phi \Vert _{L^\infty }^2 \int _{-\left( \frac{3}{4}\right) ^{2\alpha }}^0 \int _{B_{{3/4}+\delta }} \int _{B_{{3/4}+\delta }} \frac{|\eta _k(x)-\eta _k(y) |^2}{|x-y |^{2+2\alpha }} \,dy\, dx \, dt \nonumber \\&\quad \le \pi \delta ^{2\alpha } \Vert \phi \Vert _{L^\infty }^2 \int _{-\left( \frac{13}{16}\right) ^{2\alpha }}^0[\eta _k(t)]_{W^{\alpha ,2}(B_{13/16})}^2 \, dt \le C \delta ^{2\alpha }, \end{aligned}$$

where *C* does not depend on $k\ge 1$ by Step 1. Since $\eta _k$ is uniformly bounded in $L^{(1+\alpha )(p-1)}(Q_{13/16})$, we have by interpolation for some $\vartheta \in (0, 1]$ and $C \ge 1$ that

$$\begin{aligned} \Vert \eta _k-\eta _k *\phi _\delta \Vert _{L^q(Q_{3/4})} \le C \delta ^{\alpha \vartheta }. \end{aligned}$$

We now fix $\delta$ small enough, independently of *k*, such that this contribution does not exceed $\frac{\varepsilon }{3}.$ As for the third term, we consider for fixed $\delta >0$ small, the family of curves $\{t \mapsto \eta _k *\phi _\delta \}_{k\ge 1}$. From the equation, we have the identity

$$\begin{aligned} \partial _t( \eta _k *\phi _\delta ) = - (u_k \cdot \nabla \eta _k) *\phi _\delta - (-\Delta )^\alpha \eta _k *\phi _\delta . \end{aligned}$$

Observe that $u_k \cdot \nabla \eta _k = {{\,\mathrm{div}\,}}( u_k \eta _k)$ so that

$$\begin{aligned}&\Vert (u_k \cdot \nabla \eta _k) *\phi _\delta \Vert _{L^\frac{pq}{p+q}([-(3/4)^{2\alpha }, 0],W^{1, \infty }(B_{3/4}))}\\&\quad \le \Vert u_k\Vert _{L^p(Q_{3/4})} \Vert \eta _k\Vert _{L^q(Q_{3/4})} \Vert \phi _\delta \Vert _{W^{2, \frac{pq}{pq-p-q}}(B_{3/4})}. \end{aligned}$$

As for the last term, we have that for $x\in B_{3/4}$

$$\begin{aligned} |(-\Delta )^\alpha \phi _\delta (x) |&=c_\alpha \left|\int \frac{\phi _\delta (x)-\phi _\delta (y)}{|x-y |^{2+2\alpha }} \, dy \right|\\&\lesssim \Vert \phi _\delta \Vert _{C^2} \int _{B_1} \frac{\, dy}{|y |^{2\alpha }} + \Vert \phi _\delta \Vert _{L^\infty } \int _{B_1^c} \frac{\,dy}{|x-y |^{2+2\alpha }} \\&\le \frac{C(\delta )}{1+|x |^{2+2\alpha }}. \end{aligned}$$

Analogously, $|(-\Delta )^\alpha \nabla \phi _\delta (x) |\le \frac{C(\delta )}{1 + |x |^{2+2\alpha }}.$ We estimate the convolution on dyadic balls for fixed time. We set $\eta _{k, i}:= \eta _k (\mathbb {1}_{B_{2^{i+1}}} - \mathbb {1}_{B_{2^i}})$ for $i\ge 0$ and estimate

$$\begin{aligned} \Vert (-\Delta )^\alpha \phi _\delta *\eta _k\Vert _{W^{1, \infty }(B_{3/4})} \le C(\delta ) \bigg ( \int _{B_1} |\eta _k |+ \sum _{i \ge 0} \Vert (-\Delta )^\alpha \phi _\delta *\eta _{k, i}\Vert _{W^{1,\infty }(B_{3/4})} \bigg ). \end{aligned}$$

For $i \ge 0$ we observe that for $x \in B_{3/4}$ and $z \in B_{2^{i+1}} \setminus B_{2^i}$ we have $|x - z |\ge 2^{i-2}\,,$ so that

$$\begin{aligned} \sum _{i \ge 0} \Vert (-\Delta )^\alpha \phi _\delta *\eta _{k, i}\Vert _{W^{1,\infty }(B_{3/4})}&\le C(\delta )\sum _{i\ge 0} \frac{1}{2^{(i-2)(2+2\alpha )}} \int _{B_{2^{i+1}}\setminus B_{2^i}} |\eta _k |\, dy \\&\le C(\delta ) \sum _{i\ge 0} \frac{1}{2^{(i-2)(2\alpha -\sigma )}} \int _{B_{2^{i+1}}\setminus B_{2^i}} \frac{|\eta _k |}{2^{(2+\sigma )(i-2)}} \, dy\\&\le C(\delta ) \bigg ( \sup _{R \ge 1} \frac{1}{R^\sigma } \fint _{B_R} |\eta _k - [\eta _k]_1 |\, dy + \int _{B_1} |\eta _k|\, dy \bigg ). \end{aligned}$$

We conclude by integrating in time that

$$\begin{aligned} \Vert (-\Delta )^\alpha \phi _\delta *\eta _k\Vert _{L^p([-(3/4)^{2\alpha },0], W^{1, \infty }(B_{3/4}))} \le C(\delta ) \left( E^S(\eta _k;1) + E^{NL}(\eta _k;1) \right) . \end{aligned}$$

Summarizing, we have shown that

$$\begin{aligned} \Vert \partial _t (\eta _k *\phi _\delta )\Vert _{L^\frac{pq}{p+q}([-(3/4)^{2\alpha }, 0], W^{1,\infty }(B_{3/4}))} \le C(\delta ) \end{aligned}$$

uniformly in $k\ge 1$. Hence the family of curves $\{t \mapsto \eta _k *\phi _\delta \}_{k\ge 1}$ is an equicontinuous sequence with values in a bounded subset of $W^{1, \infty }(B_{3/4})$. By Arzela-Ascoli there exists a uniformly convergent subsequence (which we don’t relabel), and in particular, there exists $N=N(\delta )>0$ such that for any $k, j \ge N$ we have

$$\begin{aligned} \Vert (\eta _k-\eta _j) *\phi _\delta \Vert _{L^q(Q_{3/4})} \le \frac{\varepsilon }{3}, \end{aligned}$$

hence $\Vert \eta _k-\eta _j\Vert _{L^q(Q_{3/4})} \le \varepsilon$ for all $k, j \ge N$ which proves the claim.

*Step 3: Conclusion.*

By Step 2 we can pass to the limit in the equation in $Q_{3/4}$ and deduce that $\eta \in L^p(Q_{3/4})$ is a distributional solution of $\partial _t \eta + (-\Delta )^\alpha \eta =0$ in $Q_{3/4}.$ Moreover, by weak lower semicontinuity $E^S(\eta ;1) + E^{NL}(\eta ;1) \le 1.$ $\square$

## Decay of the Excess

In this section, we prove the self-improving property of the excess, namely that if the excess is small at any given $Q_r\,,$ there exists a small, fixed scale $\mu _0 \in (0, \frac{1}{2})$, independent of *r*, at which the excess decays between $Q_r$ and $Q_{\mu _0 r}$ - provided that the velocity field has zero average on $B_r$. This requirement is crucial to guarantee the decay of the excess related to the non-local part of the velocity (see $E^V(v_k^2;\mu )$ in the proof of Proposition 4.1). More generally, one could prove this excess decay at scale $\mu _0$ under the weaker assumption that the average of the rescaled velocity $u_r$ on $B_1$ is bounded uniformly in *r* for $r\in (0,1)$. However, since all $L^p$-norms are supercritical with respect to the scaling (4) of the equation, we will not be able to guarantee such an assumption. In this section, we will also for the first time make use of the structure of the velocity field (3). Similar arguments should apply for velocity fields determined from $\theta$ by other singular integral operators.

### Proposition 4.1

(Excess decay)

Let $\alpha \in (0, \frac{1}{2})$, $\sigma \in (0, 2\alpha )$ and $p > \max \left\{ \frac{1+\alpha }{\alpha }, \frac{2\alpha }{\sigma } \right\}$. For any $c>0$ and any $\gamma \in (0, \sigma -\frac{2\alpha }{p})$ there exist universal $\varepsilon _0= \varepsilon _0(\alpha , \sigma , p, c, \gamma ) \in (0, \frac{1}{2})$ and $\mu _0= \mu _0(\alpha , \sigma , p, c, \gamma )\in \left( 0, \frac{1}{2}\right)$ such that the following holds: Let $Q_r(x,t) \subseteq {\mathbb {R}}^2 \times (0, \infty )$ and let $(\theta , u)$ be a suitable weak solution to (1)–(2). We assume that the velocity field satisfies $\left[ u(s)\right] _{B_r(x)} = 0$ for all $s \in [t-r^{2\alpha }, t]$ and is obtained from $\theta$ by

28 $$\begin{aligned} u(y,s) = {\mathcal {R}}^\perp \theta (y,s) + f(s) \, \end{aligned}$$

for some $f \in L^1([t-r^{2\alpha }, t])$. Then, if $E(\theta , u; x, t,r) \le r^{1-2\alpha } \varepsilon _0\,,$ the excess decays at scale $\mu _0$, that is

$$\begin{aligned} E(\theta , u; x,t, \mu _0 r) \le c {\mu _0}^\gamma E(\theta , u; x,t,r). \end{aligned}$$

### Remark 4.2

If in (28) $f=0$ we recover simply the SQG equation. We will need the freedom to subtract a function of time *f* from the velocity field *u* in order to satisfy the zero-average assumption (see Lemma 5.1).

We will need the following auxiliary Lemma.

### Lemma 4.3

Assume $\theta \in L^2_{loc}({\mathbb {R}}^2)$ with ${{\,\mathrm{supp}\,}}\theta \subseteq \left( \overline{B}_{3/8}\right) ^c$ and that for some $\sigma \in (0,1)$ we have

$$\begin{aligned} \sup _{R\ge 1} \frac{1}{R^\sigma } \fint _{B_R} |\theta (x) |\, dx < + \infty . \end{aligned}$$

Then ${\mathcal {R}}^\perp \theta \in C^\infty (B_{3/8})$ and there exists a universal $C=C(\sigma )>0$ such that

$$\begin{aligned} \Vert D {\mathcal {R}}^\perp \theta \Vert _{L^\infty (B_{1/4})} \le C \sup _{R\ge 1} \frac{1}{R^\sigma } \fint _{B_R} |\theta (x) |\,dx. \end{aligned}$$

### Proof of Lemma 4.3

Observe that from $(-\Delta )^\frac{1}{2} {\mathcal {R}}^\perp \theta =0$ on $\overline{B}_{3/8}$, we infer that ${\mathcal {R}}^\perp \theta \in C^\infty (B_{3/8}).$ Moreover for $i,j=1,2$ and $x \in B_{1/4}$, we notice that the integral representation is no longer singular and we can compute by integration by parts

$$\begin{aligned} \partial _j {\mathcal {R}}^i\theta (x)= \int _{|z |\ge \frac{3}{8}} \frac{x_i-z_i}{|x-z |} \partial _j \theta (z) \, dz=- \int _{|z |\ge \frac{3}{8}} \partial _j \left( \frac{x_i-z_i}{|x-z |^3} \right) \theta (z) \, dz, \end{aligned}$$

where we used that the boundary terms at $\{ |z |= \frac{3}{8} \}$ and at infinity vanish. Observe that for $x \in B_{1/4}$ and $z \in B_{2^{i+1}} \setminus B_{2^i}$ we have $|x-z |\ge 2^i -\frac{1}{4} \ge 2^{i-1}$ for $i \ge -1.$ Thus

$$\begin{aligned} |\partial _j {\mathcal {R}}^i \theta (x) |&\le \int _{\frac{3}{8} \le |z |\le \frac{1}{2}} \Big | \partial _j \Big ( \frac{x_i-z_i}{|x-z |^3} \Big ) \theta (z) \Big | \, dz +\sum _{i \ge -1} \int _{B_{2^{i+1}} \setminus B_{2^i}} \Big | \partial _j \Big ( \frac{x_i-z_i}{|x-z |^3} \Big ) \theta (z)\Big | \, dz \\&\le C \bigg ( \int _{B_1} |\theta (z) |\, dz + \sum _{i \ge -1} \int _{B_{2^{i+1}} \setminus B_{2^i}} \frac{ |\theta (z)|}{2^{3(i-1)}} \, dz \bigg ) \\&\le C \Big ( \sum _{i \ge -1} \frac{1}{2^{(i-1)(1-\sigma )}} \Big ) \bigg ( \sup _{R\ge 1} \frac{1}{R^\sigma } \fint _{B_R} |\theta (z) |\, dz \bigg ). \end{aligned}$$

$$\square $$

### Proof of Proposition 4.1

By translation and scaling invariance, we may assume w.l.o.g. $(x,t)=(0, 0)$ and $r=1.$ We argue by contradiction. Then there exists a sequence $(\theta _k, u_k)$ of suitable weak solutions to (1)–(2) such that

- $$E(\theta _k, u_k; \mu ) > c \mu ^\gamma E(\theta _k, u_k;1) $$ for all $$\mu \in (0, \frac{1}{2})\,,$$
- $$\lim _{k \rightarrow \infty } E(\theta _k, u_k; 1) = 0\,,$$
- $[u_k(s)]_{B_1}= 0$ for all $s\in [-1,0]$ and for all $k \ge 1$,
- $$u_k(y,s)= {\mathcal {R}}^\perp \theta (y,s) + f_k(s)\,$$ for some $$f_k \in L^1([-1, 0]).$$

We set $E_k:=E(\theta _k, u_k; 1)$ and $M_k := (\theta _k)_{Q_1}$. We will consider the rescaled and shifted sequence

$$\begin{aligned} \eta _k := \frac{\theta _k - M_k}{E_k} \, \quad \text {and } \quad v_k := \frac{u_k }{E_k}. \end{aligned}$$

By construction, $(\eta _k)_{Q_1}=0$ and $E(\eta _k, v_k;1)=1.$ In particular, we have for all $\mu \in (0, \frac{1}{2})$ that

29 $$\begin{aligned} E(\eta _k, v_k; \mu ) > c \mu ^{\gamma }. \end{aligned}$$

We will now take the limit $k \rightarrow \infty$ and argue that (29) contradicts the excess decay dictated by the linear limit equation. Indeed, by Lemma 3.8, the sequence $\eta _k$ converges weakly to $\eta$ in $L^{3/2}_{loc}({\mathbb {R}}^2 \times [-1,0])$ and moreover, $\eta _k \rightarrow \eta$ strongly in $L^p(Q_{3/4}).$ Hence we have for $\mu \in (0, \frac{1}{2})$

$$\begin{aligned} E^S(\eta ; \mu ) = \lim _{k \rightarrow \infty } E^S(\eta _k; \mu ). \end{aligned}$$

We also know from Lemma 3.8 that $\eta \in L^p(Q_{3/4})$ solves the fractional heat equation $\partial _t \eta +(-\Delta )^\alpha \eta =0$ on $Q_{3/4}\,$ with $E^S(\eta ;1)+ E^{NL}(\eta ;1) \le 1.$ In particular, we deduce from Lemma A.1 that $\eta$ is smooth (in space) on $Q_{1/2}$ and that $\eta \in C^{ 1-1/p}(Q_{1/2})$ with the estimate

30 $$\begin{aligned} \Vert \eta \Vert _{L^\infty ([-(1/2)^{2\alpha }, 0], C^1(B_{1/2}))} + \Vert \eta \Vert _{C^{1-\frac{1}{p}}(Q_{1/2})} \le {\bar{C}}( E^S(\eta ; 1) + E^{NL}(\eta ;1))\le {\bar{C}}. \end{aligned}$$

In particular, we infer that for $\mu \in (0, \frac{1}{2})$

31 $$\begin{aligned} \lim _{k \rightarrow \infty } E^S(\eta _k; \mu ) =E^S(\eta ; \mu ) \le {\bar{C}} \mu ^{2\alpha (1-\frac{1}{p})}. \end{aligned}$$

Let us now consider the non-local part of the excess. We split

$$\begin{aligned} E^{NL}(\eta _k;\mu )&\le \bigg (\, \fint _{-\mu ^{2\alpha }}^0 \sup _{\frac{\mu }{4} \le R < \frac{1}{4}} \left( \frac{\mu }{R}\right) ^{\sigma p} \bigg (\,\fint _{B_R} |\eta _k(x,t) -[\eta _k(t)]_{B_\mu } |^\frac{3}{2} \, dx \bigg )^\frac{2p}{3}\, dt \bigg )^\frac{1}{p}\\&\quad +\bigg ( \,\fint _{-\mu ^{2\alpha }}^0 \sup _{R \ge \frac{1}{4}} \left( \frac{\mu }{R}\right) ^{\sigma p} \bigg (\,\fint _{B_R} |\eta _k(x,t) -[\eta _k(t)]_{B_\mu } |^\frac{3}{2} \, dx \bigg )^\frac{2p}{3} \, dt \bigg )^\frac{1}{p}. \end{aligned}$$

We estimate the second term by adding and subtracting $[\eta _k(t)]_{B_1}$ for fixed time *t*. In the sequel $C'=C'(\alpha , \sigma )$ will denote a universal constant which may change line by line. Using that $E^{NL}(\eta _k;1)+E^S(\eta _k;1) \le 1$ for all $k \ge 1$, we obtain that

$$\begin{aligned}&\bigg (\, \fint _{-\mu ^{2\alpha }}^0 \sup _{R \ge \frac{1}{4}} \left( \, \frac{\mu }{R}\right) ^{\sigma p} \bigg (\,\fint _{B_R} |\eta _k(x,t) -[\eta _k(t)]_{B_\mu } |^\frac{3}{2} \, dx \bigg )^\frac{2p}{3} \, dt \bigg )^\frac{1}{p} \\&\quad \le \mu ^{\sigma - \frac{2\alpha }{p}} E(\eta _k;1) + (4\mu )^\sigma \bigg ( \,\fint _{-\mu ^{2\alpha }}^0 |[\eta _k(t)]_{B_1}- [\eta _k(t)]_{B_\mu } |^p \, dt \bigg )^\frac{1}{p}\\&\quad \le \mu ^{\sigma - \frac{2\alpha }{p}} + (4\mu )^\sigma \Bigg (\bigg (\, \fint _{-\mu ^{2\alpha }}^0 |[\eta _k(t)]_{B_1}- [\eta _k(t) ]_{B_{1/2}} |^p \, dt \bigg )^\frac{1}{p}\\&\qquad + \bigg (\, \fint _{-\mu ^{2\alpha }}^0 |[\eta _k(t)]_{B_{1/2}}- [\eta _k(t)]_{B_\mu } |^p \, dt \bigg )^\frac{1}{p}\Bigg ) \\&\quad \le \mu ^{\sigma - \frac{2\alpha }{p}}+ C' \mu ^{\sigma - \frac{2\alpha }{p}} E^S(\eta _k;1) + (4\mu )^\sigma \bigg (\, \fint _{-\mu ^{2\alpha }}^0 |[\eta _k(t)]_{B_{1/2}}- [\eta _k(t)]_{B_\mu } |^p \, dt \bigg )^\frac{1}{p} \\&\quad \le C' \mu ^{\sigma -\frac{2\alpha }{p}} +(4\mu )^\sigma \bigg (\, \fint _{-\mu ^{2\alpha }}^0 |[\eta _k(t)]_{B_{1/2}}- [\eta _k(t)]_{B_\mu } |^p \, dt \bigg )^\frac{1}{p}. \end{aligned}$$

We infer, using the strong converge of $\eta _k \rightarrow \eta$ in $L^p(Q_{3/4})$, that

$$\begin{aligned}&\liminf _{k \rightarrow \infty } E^{NL}(\eta _k;\mu ) \le C' \mu ^{\sigma -\frac{2\alpha }{p}} +\, (4\mu )^\sigma \bigg (\, \fint _{-\mu ^{2\alpha }}^0 |[\eta (t)]_{B_{1/2}}- [\eta (t)]_{B_\mu } |^p \, dt \bigg )^\frac{1}{p}\\&\quad +\bigg (\, \fint _{-\mu ^{2\alpha }}^0 \sup _{\frac{\mu }{4} \le R < \frac{1}{4}} \left( \frac{\mu }{R}\right) ^{\sigma p} \bigg (\, \fint _{B_R} |\eta (x,t) -[\eta (t)]_{B_\mu } |^\frac{3}{2}\, dx \bigg )^\frac{2p}{3} \, dt\bigg )^\frac{1}{p}. \end{aligned}$$

Using (30) again, we obtain that $|[\eta (t)]_{B_\mu }-[ \eta (t)]_{B_{1/2}} |\le {\bar{C}}$ uniformly in time as well as

$$\begin{aligned}&\Bigg (\, \fint _{-\mu ^{2\alpha }}^0 \sup _{\frac{\mu }{4} \le R< \frac{1}{4}} \left( \frac{\mu }{R}\right) ^{\sigma p} \bigg (\,\fint _{B_R} |\eta (x,t) -[\eta (t)]_{B_\mu } |^\frac{3}{2} \bigg )^\frac{2p}{3} \Bigg )^\frac{1}{p} \\&\quad \le {\bar{C}} \bigg ( \,\fint _{-\mu ^{2\alpha }}^0 \sup _{\frac{\mu }{4} \le R < \frac{1}{4}} \left( \frac{\mu }{R}\right) ^{\sigma p} R^p \bigg )^\frac{1}{p} \le {\bar{C}} \mu ^\sigma . \end{aligned}$$

We conclude that

32 $$\begin{aligned} \liminf _{k \rightarrow \infty } E^{NL}(\eta _k;\mu ) \le C' \mu ^{\sigma -\frac{2\alpha }{p}}. \end{aligned}$$

Finally, let us consider the part of the excess which is related to the velocity $v_k.$ We observe that, using the structure of the velocity (28),

$$\begin{aligned} E^V(v_k;\mu ) = \bigg (\, \fint _{Q_\mu } \big | {\mathcal {R}}^\perp \big (E_k^{-1} \theta _k\big )(x,t)- \big [{\mathcal {R}}^\perp \big (E_k^{-1} \theta _k\big )(t)\big ]_{B_\mu } \big |^p \, dx \, dt \bigg )^\frac{1}{p}. \end{aligned}$$

We write ${\mathcal {R}}^\perp (E_k^{-1} \theta _k)= v_k^1+ v_k^2$ where we introduce

$$\begin{aligned} v_k^1:={\mathcal {R}}^\perp \left( \eta _k \chi \right) \end{aligned}$$

for some cut-off $\chi$ between $B_{3/8}$ and $B_{1/2}\,$. Correspondingly, we write

$$\begin{aligned} E^V(v_k; \mu ) \le \bigg (\,\fint _{Q_\mu } |v_k^1 - [v_k^1(t)]_{B_\mu } |^p \, dx \,dt \bigg )^\frac{1}{p} + \bigg (\,\fint _{Q_\mu } |v_k^2 - [v_k^2(t)]_{B_\mu } |^p \, dx \, dt\bigg )^\frac{1}{p}. \end{aligned}$$

By Calderon–Zygmund estimates, we infer that $v_k^1 \rightarrow {\mathcal {R}}^\perp (\eta \chi )=:v^1$ strongly in $L^p(Q_{3/4})$. Moreover by Schauder estimates [29, Proposition 2.8], we have for fixed time

$$\begin{aligned}{}[v^1(t)]_{C^{1-\frac{1}{p}}({\mathbb {R}}^2)} \le C' \Vert \eta (t)\chi \Vert _{C^{1-\frac{1}{p}}({\mathbb {R}}^2)} \le C' \Vert \eta (t)\Vert _{C^{1-\frac{1}{p}}(Q_\frac{1}{2})} \le C' {\bar{C}} \end{aligned}$$

uniformly in $t\in [-(1/2)^{2\alpha }, 0]$ by (30). We conclude that

$$\begin{aligned} \lim _{k \rightarrow \infty } \bigg (\,\fint _{Q_\mu } |v_k^1 - [v_k^1(t)]_{B_\mu } |^p \, dx \, dt \bigg )^\frac{1}{p} = \bigg (\, \fint _{Q_\mu } |v^1-[v^1(t)]_{B_\mu } |^p \, dx \, dt \bigg )^\frac{1}{p}\le C' \mu ^{1-\frac{1}{p}}. \end{aligned}$$

We now come to the excess related to $v_k^2.$ By construction,

$$\begin{aligned} v_k^2 = {\mathcal {R}}^\perp \bigg (\frac{\theta _k}{E_k} - \eta _k \chi \bigg ) = {\mathcal {R}}^\perp \bigg ( \eta _k (1-\chi ) + \frac{M_k}{E_k} \bigg ). \end{aligned}$$

Correspondingly, we define $w_{k,\rho }^1:={\mathcal {R}}^\perp ( \eta _k(1-\chi ) \chi _\rho )$ and $w_{k,\rho }^2 := {\mathcal {R}}^\perp \left( \frac{M_k}{E_k} \chi _\rho \right)$ for some radially symmetric cut-off $\chi _\rho$ between $B_\rho$ and $B_{\rho +1}.$ By Calderon–Zygmund, we have for fixed time *t* that $w_{k,\rho }^1(t) + w_{k,\rho }^2(t) \rightarrow v_k^2(t)$ as $\rho \rightarrow \infty$ strongly in $L^p({\mathbb {R}}^2).$ In particular,

$$\begin{aligned}&\bigg (\,\fint _{Q_\mu } | v_k^2 - [v_k^2(t)]_{B_\mu } |^p \, dx \, dt \bigg )^\frac{1}{p} \\&\quad = \lim _{\rho \rightarrow \infty }\bigg (\,\fint _{Q_\mu } |w_{k,\rho }^1 + w_{k, \rho }^2 - [(w_{k,\rho }^1 + w_{k, \rho }^2)(t)]_{B_\mu } |^p \, dx \, dt \bigg )^\frac{1}{p} \\&\quad \le \limsup _{\rho \rightarrow \infty }\bigg (\,\fint _{Q_\mu } |w_{k,\rho }^1 - [w_{k,\rho }^1(t) ]_{B_\mu } |^p \, dx \, dt \bigg )^\frac{1}{p} \\&\qquad + \limsup _{\rho \rightarrow \infty }\bigg (\,\fint _{B_\mu } |w_{k, \rho }^2 - [w_{k, \rho }^2(t)]_{B_\mu } |^p \, dx \, dt \bigg )^\frac{1}{p}. \end{aligned}$$

Let us consider first $w_{k,\rho }^1$. We apply Lemma 4.3 to $w_{k,\rho }^1$ to deduce that for fixed time *t*

$$\begin{aligned}{}[w_{k,\rho }^1(t)]_\mathrm{Lip(B_{1/4})}&\le C' \sup _{R \ge 1} \frac{1}{R^\sigma } \fint _{B_R} |\eta _k |(x,t) \, dx \\&\le C' \bigg ( \sup _{R \ge 1} \frac{1}{R^\sigma } \fint _{B_R} |\eta _k(x,t) - [\eta _k(t)]_{B_1} |\, dx + \int _{B_1} |\eta _k|(x,t) \, dx \bigg ). \end{aligned}$$

Integrating in time, we infer that uniformly in $\rho \ge 1$

$$\begin{aligned} \Vert w_{k,\rho }^1\Vert _{L^p([-1, 0], \mathrm Lip(B_{1/4}))} \le C' \left( E^S(\eta _k;1) +E^{NL}(\eta _k;1)\right) . \end{aligned}$$

We deduce that for $\mu \in (0, \frac{1}{4})$ we have

33 $$\begin{aligned} \lim _{\rho \rightarrow \infty }\bigg (\, \fint _{Q_\mu } |w_{k, \rho }^1(x,t) - [w_{k,\rho }^1(t)]_{B_\mu } |^p \, dx \, dt \bigg )^\frac{1}{p} \le C' \mu . \end{aligned}$$

We now come to the contribution of $w_{k, \rho }^2$. Observe that $(-\Delta )^{1/2} \, w_{k,\rho }^2 = 0$ in $B_1$ for $\rho \ge 1$ such that $w_{k,\rho }^2$ is smooth in the inside of $B_1$. Recall moreover, that we have the integral representation (in the principal value sense)

$$\begin{aligned} \big (w_{k, \rho }^2\big )^\perp (x) =-\, c\, \frac{M_k}{E_k}\int \frac{x-y}{|x-y |^3} \chi _\rho (y) \, dy \, \end{aligned}$$

so that, by spherical symmetry of $\chi _\rho$, we immediately infer $w_{k,\rho }^2(0)=0.$ Moreover, for $x \in B_{1/2}$ we have

$$\begin{aligned} |w_{k, \rho }^2 (x) |&=c \frac{|M_k |}{E_k} \bigg |\int _{\rho< \vert x-y |< \rho +1} \frac{y}{|y |^3} \chi _\rho (x-y) \, dy \bigg |\\&\le c \frac{|M_k |}{E_k} \pi ((\rho +1)^2 - \rho ^2) \left( \frac{\rho }{2}\right) ^{-2}=C' \frac{|M_k |}{E_k} \rho ^{-1}. \end{aligned}$$

Thus for fixed $k\ge 1,$ we have

$$\begin{aligned} \lim _{\rho \rightarrow 0} \Vert w_{k,\rho }^2\Vert _{L^\infty (B_{1/2})} = 0\,, \end{aligned}$$

so that excess associated to $v_{k,2}$ is controlled by (33). Collecting the terms (31) and (32) and taking the limes inferior $k \rightarrow \infty$ in (29) we have obtained, for a universal constant $C'=C'(\alpha , \sigma )>0\,,$ that

$$\begin{aligned} c{\mu }^\gamma \le C' \mu ^{\sigma - \frac{2\alpha }{p}}= {\mu }^\gamma C'\mu ^{\sigma - \frac{2\alpha }{p}-\gamma }. \end{aligned}$$

for all $\mu \in (0, \frac{1}{4}).$ We reach the desired contradiction for

$$\begin{aligned} \mu \le \left( \frac{c}{C' } \right) ^\frac{1}{\sigma - \frac{2\alpha }{p}-\gamma }. \end{aligned}$$

$$\square $$

## Iteration of the Excess Decay

In this section, we prove the decay of the excess on all scales. We iteratively define shifted rescalings of $$(\theta , u)$$ verifying the zero average assumption of Proposition 4.1 as well as (28) and therefore allowing the decay of the excess when passing at scale $$\mu _0$$. From the decay of the excess on all scales, we deduce Hölder continuity by means of Campanato’s Theorem. In contrast to Navier-Stokes, we need our estimates to be quantitative, since it is not known whether local smoothness for SQG follows from a mere $$L^\infty $$-bound; instead we need to prove spatial $$C^{\delta }$$-Hölder continuity of the velocity for a $$\delta >1-2\alpha $$ (see Lemma B.1). The main mechanism of the iteration is the invariance of the equation under the following change of variables realizing the zero average assumption on $$B_{1/4}. $$ The latter has been exploited previously in [3, 10].

### Lemma 5.1

(Change of variables at unit scale)

Let $\alpha \in (\frac{1}{4}, \frac{1}{2})$ and $\sigma \in (0, 2\alpha )$. Let $(\theta , u)$ be a suitable weak solution of (1) on ${\mathbb {R}}^2 \times [-4^{2\alpha },0]$. Fix $(x, t) \in Q_1.$ Define $\theta _0(y,s):= \theta (y+x+x_0(s), s+t)\,$ and $u_0(y,s):=u(y+x+x_0(s), s+t) - \dot{x}_0(s)\,,$ where

34 $$\begin{aligned} {\left\{ \begin{array}{ll}\dot{x}_0(s) &{}= \displaystyle \fint _{B_\frac{1}{4}} u(y + x_0(s)+ x,s+t) \,dy \\ x_0(0)&{}=0. \end{array}\right. } \end{aligned}$$

Then $(\theta _0, u_0)$ is a suitable weak solution of (1) on ${\mathbb {R}}^2 \times [-1, 0]$ with $[u_0(s)]_{B_{1/4}}=0$ for $s \in [-1,0].$ Moreover, there exists universal $\varepsilon _1=\varepsilon _1(p, \alpha ) \in (0,\frac{1}{2})$ and $C_1\ge 1$ such that if

35 $$\begin{aligned} \left( \fint _{Q_1(x,t)} |u |^p \,dy \, ds\right) ^\frac{1}{p} \le \varepsilon _1, \end{aligned}$$

then

$$\begin{aligned} E\left( \theta _0, u_0 ; \frac{1}{4}\right) \le C_1 E(\theta , u;x,t,1). \end{aligned}$$

### Remark 5.2

(Change of variables at scale *r*)

Under the hypothesis of Lemma 5.1, the rescaled pair ($\theta _r$, $u_r$) is still a suitable weak solution for $r \in (0,1)$ (see (4)) and we can apply the change of variables of Lemma 5.1 to it. More precisely, we define for $(x,t) \in Q_1$

$$\begin{aligned} \theta _{r,0}(y,s):=r^{2\alpha -1} \theta (r(y + x_0(s)+ x), r^{2\alpha }(s+t)) \end{aligned}$$

and

$$\begin{aligned} u_{r,0}(y,s)= r^{2\alpha -1} u(r(y + x_0(s)+x), r^{2\alpha }(s + t))-\dot{x}_0(s)\,, \end{aligned}$$

where $$\dot{x}_0(s)=r^{2\alpha -1} \fint _{B_{1/4}} u(r(y+x_0(s)+x), r^{2\alpha }(s+ t))$$ with $$ x_0(0)=0.$$ Observe that equivalently, by considering $$\tilde{x}_0(s):=r^{1-2\alpha } x_0(s)$$, we can write

$$\begin{aligned} \theta _{r,0}(y,s):=r^{2\alpha -1} \theta (r y + r^{2\alpha } {\tilde{x}}_0(s)+r x, r^{2\alpha }(s+t)) \end{aligned}$$

and

$$\begin{aligned} u_{r,0}(y,s)= r^{2\alpha -1} \left( u(r y + r^{2\alpha } {\tilde{x}}_0(s)+ rx, r^{2\alpha }(s + t))-\dot{ \tilde{x}}_0(s)\right) . \end{aligned}$$

### Proof

By Peano, the ODE (34) admits a solution and we claim it is unique since the vectorfield generating the flow is log-Lipschitz and hence satisifies the Osgood uniqueness criterion [34, Chapter II.7 and III.12.7-8]. Indeed, we know from Theorem 3.2 that $\theta \in L^\infty ({\mathbb {R}}^2 \times [-2, 0])$ and $u \in L^\infty ( [-2,0], {BMO}({\mathbb {R}}^2)).$ We estimate for fixed time $\tau$ as long as $|\xi - \zeta |\le \frac{1}{4}$, using also the bound $|B_\frac{1}{4}(\xi ) \Delta B_\frac{1}{4}(\zeta ) |\lesssim |\xi - \zeta |$ on the volume of the symmetric difference,

$$\begin{aligned}&\bigg | \, \fint _{B_\frac{1}{4}(\xi )} u(y+x, \tau ) \, dy - \fint _{B_\frac{1}{4}( \zeta )} u(y+x,\tau ) \, dy \, \bigg | \\&\quad \lesssim |\xi -\zeta |\bigg | \, \fint _{B_\frac{1}{4}(\xi )\setminus B_\frac{1}{4}( \zeta ) } u(y+x,\tau ) \, dy- \fint _{B_\frac{1}{4}(\zeta ) \setminus B_\frac{1}{4}(\xi )} u(y+x,\tau ) \, dy \, \bigg | \\&\quad \lesssim |\xi -\zeta |\bigg ( \, \fint _{B_\frac{1}{4}(\xi ) \setminus B_\frac{1}{4}(\zeta )} \big | u(y+x,\tau ) - [u(\cdot +x, \tau )]_{B_{3/8}(\frac{1}{2}(\xi + \zeta ))} \big | \, dy \\&\, \qquad +\fint _{B_\frac{1}{4}(\zeta ) \setminus B_\frac{1}{4}(\xi )} \big | u(y+x, \tau ) - [u(\cdot +x,\tau )]_{B_{3/8}(\frac{1}{2}(\xi + \zeta ))} \big | \, dy \bigg ) \, . \end{aligned}$$

Recall from the John-Nirenberg inequality [19] that BMO functions are exponentially integrable, that is for every $f \in {BMO}({\mathbb {R}}^2)$ there exist constants $c_1, c_2 >0$ such that for any ball *B* in ${\mathbb {R}}^2$

$$\begin{aligned} |\{ x \in B: |f - [f]_B|> \lambda \} |\le c_1 \exp (- c_2 \lambda \Vert f\Vert _{{BMO}}^{-1}) |B |. \end{aligned}$$

As an immediate consequence, we observe that for $A \ge C \Vert f\Vert _{{BMO}}\,$ and any ball *B* in ${\mathbb {R}}^2$

$$\begin{aligned} \sup _{B } \fint _{B} e^{\frac{|f- [f]_B |}{A}} \, dx < + \infty . \end{aligned}$$

We now estimate the last two contributions, setting $z:= x + \frac{1}{2} (\xi + \zeta )$ and using Jensen

$$\begin{aligned}&\fint _{B_\frac{1}{4}(\xi ) \setminus B_\frac{1}{4}(\zeta )} \big | u(y+x, \tau ) - [u(\cdot +x,\tau )]_{B_{3/8}(\frac{1}{2}(\xi + \zeta ))} \big | \, dy \\&\quad \lesssim \Vert u(\tau )\Vert _{BMO} \, \log \bigg (|B_\frac{1}{4}(\xi ) \setminus B_\frac{1}{4}(\zeta ) |^{-1} \,\fint _{B_\frac{3}{8}(z)} e^{ \frac{1}{A} |u(y, \tau ) - [u(\tau )]_{B_{3/8}(z)} |}\, dy \bigg ) \\&\quad \lesssim \Vert u(\tau )\Vert _{ {BMO}} \log ( C |\xi - \zeta |^{-1} ). \end{aligned}$$

We infer that the function $\xi \mapsto \fint _{B_{1/4}(\xi )} u(y, s+t)$ is log-Lipschitz in space for $s \in [-1, 0]$, uniformly in time, and there is a unique solution to (34). Observe also that the dependence with respect to the point $(x,t) \in Q_1$ is log-Lipschitz. The functions $\theta _0$ and $u_0$ as in the statement are now well-defined. We remark first that, in the sense of distributions, ${{\,\mathrm{div}\,}}u_0= 0$ and

$$\begin{aligned} \partial _s \theta _0 + u_0 \cdot \nabla _y \theta _0&= (\partial _t \theta + {\dot{x}}_0(s) \cdot \nabla \theta ) \big \vert _{(y+x+ x_0(s),s+t)}\\&\quad + (u \cdot \nabla \theta - {\dot{x}}_0(s) \cdot \nabla \theta )\big \vert _{(y +x+ x_0(s), s+t)} \\&=-(-\Delta )^\alpha \theta (y+x+x_0(s), s+t)\\&=-(-\Delta )^\alpha \theta _0, \end{aligned}$$

so that $(\theta _0, u_0)$ is a distributional solution of (1). It is straightforward to check that $(\theta _0, u_0)$ is in fact a suitable weak solution. Moreover, $u_0(y,s) ={\mathcal {R}}^\perp \theta _0 (y,s)- \dot{x_0}(s)$ and

$$\begin{aligned}{}[u_0(s)]_{B_\frac{1}{4}}= \fint _{B_\frac{1}{4}} u(y+ x+x_0(s), s+t) \, dy - \dot{x}_0(s)= 0 \end{aligned}$$

by construction. Assume now that (35) holds for an $\varepsilon _1 \in (0, \frac{1}{2})$ yet to be chosen small enough. As long as $x_0(s) \in B_{3/4}$ we estimate

$$\begin{aligned} |\dot{x}_0(s) |\le \bigg (\, \fint _{B_\frac{1}{4}+ x_0(s)} |u |^p(x+y, s+t) \, dy \bigg )^\frac{1}{p} \le 4^{\frac{2}{p}}\bigg (\, \fint _{B_1} |u |^p(x+y, s+t) \, dy \bigg )^\frac{1}{p}\,, \end{aligned}$$

so that for $s\in (-1,0]$

$$\begin{aligned} |x_0(s) |\le \Vert \dot{x_0}\Vert _{L^p((-1,0))} |s |^{1-\frac{1}{p}} \le 4^\frac{2}{p} \bigg (\,\fint _{Q_1(x,t)} |u |^p\, dy \, d \tau \bigg )^\frac{1}{p}|s |^{1-\frac{1}{p}}. \end{aligned}$$

Choosing $\varepsilon _1\le \frac{3}{16} 4^{-\frac{2}{p}}\,$ the assumption (35) guarantees that $x_0(s) \in B_{3/16}\subset B_{3/4}$ for $s\in [-1,0].$ We then estimate, using again that $B_{1/4}+x_0(s) \subseteq B_1$ for $s\in [-1,0]$,

$$\begin{aligned} E^S\bigg (\theta _0; \frac{1}{4}\bigg )&\le \bigg (\, \fint _{Q_\frac{1}{4}} |\theta (y+x+x_0(s), s+t)- (\theta )_{Q_1(x,t)} |^p \, dy \, ds \bigg )^\frac{1}{p} + |(\theta _0)_{Q_\frac{1}{4}} - (\theta )_{Q_1(x,t)} |\\&\le 2 \bigg (\,\fint _{-(1/4)^{2\alpha }}^0 \fint _{B_\frac{1}{4}+ x_0(s)} |\theta (x+y, s+t)- (\theta )_{Q_1(x,t)} |^p \, dy \, ds \bigg )^\frac{1}{p} \\&\le 2 \,4^{(2+2\alpha )\frac{1}{p}} \bigg (\, \fint _{Q_1(x,t)} |\theta - (\theta )_{Q_1(x,t)} |^p \, dy \, ds\bigg )^\frac{1}{p} \\&\le 8 \, E^S(\theta ;x,t,1). \end{aligned}$$

Proceeding analogously, we have that $E^V(u_0; \frac{1}{4}) \le 16\, E^V(u; x,t,1).$ As for the non-local part of the excess, we observe that for $R \ge \frac{1}{16}$ we have $B_R(x_0(s)) \subseteq B_{R+3/16} \subseteq B_{4R}$ and hence

$$\begin{aligned}&E^{NL}\left( \theta _0; \frac{1}{4}\right) \le \bigg (\, \fint _{-(1/4)^{2\alpha }}^0 \sup _{R \ge \frac{1}{16}} \bigg \{ \Big (\frac{1}{4R} \Big )^{\sigma p} \bigg (\, \fint _{B_R} |\theta _0 -[\theta ]_{B_1(x)} |^\frac{3}{2} \, dy \,\bigg )^\frac{2p}{3}\\&\qquad + |[\theta _0]_{B_\frac{1}{4}} - [\theta ]_{B_1(x)} |^p \bigg \} \, ds \bigg )^\frac{1}{p} \\&\quad \le 8 \bigg (\, \fint _{-(1/4)^{2\alpha }}^0 \sup _{R \ge \frac{1}{16}} \Big (\frac{1}{4R} \Big )^{\sigma p} \bigg (\, \fint _{B_{4R}} |\theta (y+x, s+t) -[\theta ]_{B_1(x)} |^\frac{3}{2} \, dy\bigg )^\frac{2p}{3} \, ds \bigg )^\frac{1}{p}\\&\quad +4^{\sigma +1} E^S(\theta ;x,t, 1) \\&\quad \le 16 \left( E^{NL}(\theta ;x,t,1) + E^S(\theta ; x,t, 1) \right) . \end{aligned}$$

$$\square $$

### Theorem 5.3

Let $\alpha \in (\frac{1}{4}, \frac{1}{2})$, $\sigma \in (0, 2\alpha )$, $p > \max \left\{ \frac{1+\alpha }{\alpha }, \frac{2\alpha }{\sigma }\right\}$ and $\gamma \in [1-2\alpha , \sigma - \frac{2\alpha }{p})$. There exists $\varepsilon _2 \in \left( 0, \frac{1}{2}\right)$ (depending only on $\alpha , \sigma , p$ and $\gamma$) such that the following holds: Let $(\theta , u)$ be a suitable weak solution to (1)–(3) on ${\mathbb {R}}^2 \times (-4^{2\alpha },0]$. Assume that for any $(x,t) \in Q_1$ it holds that

36 $$\begin{aligned} E\left( \theta _0, u_0; \frac{1}{4}\right) \le \varepsilon _2, \end{aligned}$$

where $(\theta _0, u_0)$ is obtained from $\theta$ through the change of variables of Lemma 5.1. Then

37 $$\begin{aligned} \theta \in C^{\delta , \frac{p-1}{p} \delta } (Q_1) \qquad \text{ where } \qquad \delta := \gamma - \frac{1}{p-1}\left[ 1-2\alpha + \frac{2\alpha }{p} \right] . \end{aligned}$$

### Remark 5.4

(Role of the parameters) The parameter *p* is crucial since it determines the dimension of the singular set (see proof of Theorem 1.1): the lower the power *p*,  the better the dimension estimate. All the other parameters are of technical nature; yet, the range of admissible parameters is sufficiently large to allow us to conclude the desired estimate on the size of the singular set for all fractional orders for which the latter is meaningful (i.e. for $\alpha > \alpha _0$, see Remark 6.7). We deliberately choose to leave all the parameters free to increase the readability of the paper; but one could also read the paper fixing the parameters as in the proof of Theorem 1.3. Let us now comment on the role of the single parameters in more detail:

- *p* cannot go below the threshold $\frac{1+\alpha }{\alpha }\,:$ This corresponds to spacetime integrability that guarantees the compactness of the $(p-1)$-energy inequality (see Lemma 3.8) which in turn is *the* crucial ingredient of the excess decay. The requirement $p> \frac{2\alpha }{\sigma }$ on the other hand is purely technical and harmless for $\sigma$ close to $2\alpha .$
- $\sigma$ captures the decay at infinity of the non-local part of both the fractional Laplacian and the velocity and should be thought arbitrarily close to $2\alpha .$
- $\gamma$ describes the decay of the excess when passing to the smaller scale $\mu _0.$ In order to apply the excess decay of Proposition 4.1 iteratively, we have to verify its smallness requirement along a sequence of by $\mu :=\frac{\mu _0}{4}$ rescaled solutions which is possible only if the decay rate beats the supercritical scaling of the excess (see Remark 3.7), i.e. $\gamma \ge 1-2\alpha .$
- The exponent of the local Hölder continuity in space, $\delta \,,$ is obtained from $\gamma \,,$ but considerably worsened. This stems form the fact that in order to use the decay of the excess on all scales to deduce Hölder continuity via Campanato’s Theorem, we have to control the effect of the flow of Lemma 5.1. This loss in the Hölder continuity exponent is peculiar to SQG and is not observed in the similar results for Navier-Stokes.

### Proof

Let $\varepsilon _1>0$, $C_1\ge 1$ be the universal constants from Lemma 5.1.

The proof relies on an iterative construction. We fix $(x,t) \in Q_1$. We obtain the suitable weak solution $(\theta _0, u_0)$ by applying Lemma 5.1 to $(\theta , u)$ at the point (*x*, *t*). This first change of variables does two things: It translates (*x*, *t*) to the origin (0, 0) and it produces a new suitable weak solution whose velocity $u_0$ has zero average on $B_{1/4}.$ Hereafter, the excess will always be centered in (0, 0).

Let $\mu :=\frac{\mu _0}{4} \in (0, \frac{1}{8})$ where $\mu _0, \,\varepsilon _0 \in (0, \frac{1}{2})$ are given by Proposition 4.1 with $c=(16C_1)^{-1}$. For $k\ge 1$ we iteratively define a new suitable weak solution $(\theta _k, u_k)$ which we obtain from $(\theta _{k-1}, u_{k-1})$ by first rescaling it at scale $\mu$ according to (4), i.e. we set

$$\begin{aligned} \theta _{k-1, \mu }(y,s):= \mu ^{2\alpha -1} \theta _{k-1}(\mu y, \mu ^{2\alpha }s) \quad u_{k-1, \mu }(y,s):= \mu ^{2\alpha -1} u_{k-1}(\mu y, \mu ^{2\alpha }s)\,, \end{aligned}$$

and second, by applying the change of variables of Lemma 5.1 to $(\theta _{k-1, \mu }, u_{k-1, \mu })$ at the point (0, 0). This change of variables produces a new suitable weak solution, which we call $(\theta _k, u_k)\,,$ that evolves along the flow ${{x}}_k$ and whose velocity $u_k$ has zero average on $B_{1/4}.$ Indeed, setting $\tilde{x}_k(s):= \mu ^{1-2\alpha } {{x}}_k(s)\,$ (compare also with Remark 5.2), we define iteratively for $k \ge 1$

38 $$\begin{aligned} \theta _k(y,s):= \theta _{k-1, \mu }(y + {{x}}_k(s),s) =\mu ^{2\alpha -1} \theta _{k-1}(\mu y + \mu ^{2\alpha } \tilde{x}_k(s), \mu ^{2\alpha }s)\, \end{aligned}$$

and

39 $$\begin{aligned} u_{k}(y,s):= & {} u_{k-1, \mu }(y + {{x}}_k(s),s)- \dot{{{x}}}_k(s)\nonumber \\= & {} \mu ^{2\alpha -1}( u_{k-1}(\mu y +\mu ^{2\alpha } \tilde{x}_k(s), \mu ^{2\alpha }s ) - \dot{\tilde{x}}_k(s)), \end{aligned}$$

where

$$\begin{aligned} {\left\{ \begin{array}{ll}\dot{\tilde{x}}_k(s) &{}= \displaystyle \fint _{\mu ^{2\alpha -1}\tilde{x}_k(s) +B_\frac{1}{4}} u_{k-1}(\mu y,\mu ^{2\alpha }s) \,dy \\ \tilde{x}_k(0)&{}=0. \end{array}\right. } \end{aligned}$$

Observe that by Lemma 5.1 and scaling invariance, $(\theta _k, u_k)$ are suitable weak solutions of (1) for all $k\ge 0$ and $[u_k(s)]_{B_{1/4}}=0$ for all $s \in [-1,0].$

Next, we want to deduce the Hölder continuity of $\theta$ assuming (36) is enforced. To this end, we break the parabolic scaling and we consider in Step 3 a new excess of $\theta _0$ made on modified cylinders. This in turn is helpful to get sharper estimates at the level of the change of variable, performed in Step 2, since the translation has less time to act. Finally, we rewrite this decay in Step 4 in terms of $\theta$ rather than $\theta _0$, and we apply Campanato’s Theorem to deduce the Hölder continuity of $\theta$ in Step 5.

*Step 1: excess decay on the sequence of solutions after the change of variable. Let* $\alpha$, $\sigma$, *p* *and* $\gamma$ *as in the statement. There exists a universal constant* ${\bar{\varepsilon }}_2 \in (0, \frac{1}{2})$ (*depending only on* $\alpha$, $\sigma$, $\gamma$ *and* *p*) *such that if* $\varepsilon _2 \in (0, {\bar{\varepsilon }}_2]$ *and if* $(\theta ,u)$ *is a suitable weak solution to* (1) *on* ${\mathbb {R}}^2 \times (-2^{2\alpha },0]$ *with* (36), *then for every* $k\ge 0$ *the excess of* $(\theta _k, u_k)$ (*see* (38)– (39)), *decays at scale* $\mu$:

40 $$\begin{aligned} E(\theta _k, u_k; \mu )&\le C_1^{-1} { \mu ^{\gamma (k+1)} \mu ^{(2\alpha -1)k} \varepsilon _2 } \end{aligned}$$

41 $$\begin{aligned} E(\theta _k, u_k ; \frac{1}{4} )&\le \mu ^{(\gamma -(1-2\alpha ))k} \varepsilon _2 , \end{aligned}$$

*where* $C_1$ *is the universal constant from Lemma* 5.1.

We proceed by induction on $k \ge 0$.

*The case* $k=0$. Let $\varepsilon _2 \in (0,{\bar{\varepsilon }}_2]$ for some ${\bar{\varepsilon }}_2 \in (0, \frac{1}{2})$ to be chosen later and assume that (36) holds. We only need to show (40). If

$$\begin{aligned} {\bar{\varepsilon }}_2 \le 4^{2\alpha -1} \varepsilon _0\,, \end{aligned}$$

then by Proposition 4.1 and (36)

$$\begin{aligned} E(\theta _0, u_0; \mu )=E(\theta _0, u_0; \frac{\mu _0}{4}) \le C_1^{-1} \left( \frac{\mu _0}{4}\right) ^\gamma E\left( \theta _0, u_0; \frac{1}{4}\right) \le C_1^{-1} \mu ^\gamma \varepsilon _2. \end{aligned}$$

*The inductive step.* By the inductive hypothesis, we can assume that

- $$E(\theta _{k-1}, u_{k-1}; \mu ) \le C_1^{-1} \mu ^{k\gamma } \mu ^{(k-1)(2\alpha -1)} \varepsilon _2, $$
- $$E(\theta _{k-1}, u_{k-1}; \frac{1}{4}) \le \mu ^{(k-1)(\gamma -(1-2\alpha ))} \varepsilon _2.$$

We recall that $(\theta _k, u_k)$ is obtained by applying the change of variables of Lemma 5.1 to $(\theta _{k-1, \mu }, u_{k-1, \mu })$ at the point (0, 0). Using the inductive assumption and that $[u_{k-1}(s)]_{B_{1/4}}=0$ for $s \in [-1,0]$, we can verify the smallness assumption of Lemma 5.1. Indeed,

$$\begin{aligned} \bigg (\, \fint _{Q_1}|u_{{k-1}, \mu } |^p \, dy \, ds\bigg )^\frac{1}{p}&= \mu ^{2\alpha -1} \bigg (\, \fint _{Q_\mu } |u_{k-1} |^p \, dy \, ds \bigg )^\frac{1}{p} \\&\le \mu ^{2\alpha -1} \left( 4\mu \right) ^{-(2+2\alpha )\frac{1}{p}} \bigg (\, \fint _{Q_\frac{1}{4}} |u_{k-1} |^p \, dy \, ds \bigg )^\frac{1}{p} \\&=\mu ^{2\alpha -1} \left( 4\mu \right) ^{-(2+2\alpha )\frac{1}{p}} \bigg (\, \fint _{Q_\frac{1}{4}} |u_{k-1}(y,s) -[u_{k-1}(s)]_{B_\frac{1}{4}}|^p \, dy \, ds \bigg )^\frac{1}{p} \\&= \mu ^{2\alpha -1} \left( 4\mu \right) ^{-(2+2\alpha )\frac{1}{p}} E^V\left( u_{k-1}; \frac{1}{4}\right) \\&\le \mu ^{2\alpha -1} \left( 4\mu \right) ^{-(2+2\alpha )\frac{1}{p}} \varepsilon _2. \end{aligned}$$

Choosing ${\bar{\varepsilon }}_2$ even smaller, namely,

$$\begin{aligned} {\bar{\varepsilon }}_2 :=\min \big \{ ( 4^{2\alpha -1} \varepsilon _0, \mu ^{1-2\alpha } \left( 4 \mu \right) ^{(2+2\alpha )\frac{1}{p}}\varepsilon _1 \big \} \end{aligned}$$

we have

$$\begin{aligned} \bigg (\,\fint _{Q_1}|u_{{k-1}, \mu } |^p \, dy \, ds \bigg )^\frac{1}{p} \le \varepsilon _1. \end{aligned}$$

By Lemma 5.1, Remark 3.7 and the inductive hypothesis, we deduce that

$$\begin{aligned} E\left( \theta _k, u_k;\frac{1}{4}\right) \le C_1 E(\theta _{k-1, \mu }, u_{k-1, \mu }; 1) = C_1 \mu ^{2\alpha -1} E(\theta _{k-1}, u_{k-1}; \mu ) \le \mu ^{k(\gamma -(1-2\alpha ))} \varepsilon _2, \end{aligned}$$

showing the second inequality and, recalling the choice of $\varepsilon _2 \in (0, {\bar{\varepsilon }}_2)$, showing in particular that

$$\begin{aligned} E\left( \theta _k, u_k;\frac{1}{4}\right) \le 4^{2\alpha -1} \varepsilon _0. \end{aligned}$$

Since by construction $[u_k(s)]_{B_{1/4}}=0$ for $s \in [-1,0]$, we infer from Proposition 4.1 and the inductive assumption that

$$\begin{aligned} E(\theta _k, u_k; \mu ) \le C_1^{-1} \mu ^\gamma E\left( \theta _k, u_k; \frac{1}{4}\right) \le C_1^{-1} \mu ^\gamma \mu ^{k(\gamma -(1-2\alpha ))} \varepsilon _2 = C_1^{-1} \mu ^{(k+1)\gamma } \mu ^{k(2\alpha -1)} \varepsilon _2. \end{aligned}$$

*Step 2: bound on the translation in the change of variables. We observe that* $\theta _k$ *is just a shifted and rescaled (by* $\mu ^k$, *according to the natural scaling* (4)) *version of* $\theta _0$. *Indeed, notice that by construction, one can verify inductively for* $k\ge 1$

42 $$\begin{aligned} \theta _k(y,s)= \theta _{0, \mu ^k}(y+ \mu ^{-k} r_k(s), s), \end{aligned}$$

*where* $$\theta _{0, \mu ^k}(y,s):= \mu ^{k(2\alpha -1)} \theta _0(\mu ^k y, \mu ^{2\alpha k} s)$$ *and *

$$\begin{aligned} r_k(s) := \mu ^{2\alpha -1}\sum _{j=1}^k \mu ^j \tilde{x}_j(\mu ^{2\alpha (k-j)} s). \end{aligned}$$

We claim that the center of the cylinders don’t move too much, namely for $s\in [-1,0]$

$$\begin{aligned} |r_k(s) |\le C \varepsilon _2|s|^{1-\frac{1}{p}}\mu ^{2\alpha k(1-\frac{1}{p})-(1-2\alpha + \frac{2}{p})}. \end{aligned}$$

Indeed, for $j\ge 1$ we estimate as long as $x_j(s) \in B_{1/4}$

$$\begin{aligned} |\dot{\tilde{x}}_j(s) |&\le \bigg (\,\fint _{\mu ^{2\alpha } \tilde{x}_j(s) + B_\frac{\mu }{4}} |u_{j-1}(y, \mu ^{2\alpha }s )|^p \, dy \bigg )^\frac{1}{p}\\&\le \mu ^{-\frac{2}{p}} \bigg (\, \fint _{B_\frac{1}{4}} |u_{j-1}(y, \mu ^{2\alpha }s)- [u_{j-1}(\mu ^{2\alpha }s)]_{B_\frac{1}{4}} |^p \, dy \bigg )^\frac{1}{p}, \end{aligned}$$

where we used that $[u_{j-1}(\mu ^{2\alpha }s)]_{B_{1/4}} =0$ uniformly in time. In particular,

$$\begin{aligned} \Vert \dot{\tilde{x}}_j\Vert _{L^p((-1,0))} \le \mu ^{-\frac{2}{p}} 4^{-\frac{2\alpha }{p}} E^V\left( u_{j-1};\frac{1}{4}\right) \le \mu ^{-\frac{2}{p}} E\left( \theta _{j-1},u_{j-1};\frac{1}{4}\right) \end{aligned}$$

and hence for $s\in [-1,0]$ we have, using (41),

$$\begin{aligned} |\tilde{x}_j(s) |\le \mu ^{-\frac{2}{p}} E\left( \theta _{j-1},u_{j-1};\frac{1}{4}\right) |s |^{1-\frac{1}{p}} \le \varepsilon _2 |s |^{1-\frac{1}{p}} \mu ^{-\frac{2}{p}}. \end{aligned}$$

Collecting terms, we have

$$\begin{aligned} |r_k(s) |&\le \varepsilon _2|s|^{1-\frac{1}{p}} \mu ^{2\alpha k(1-\frac{1}{p})}\mu ^{2\alpha -1} \mu ^{-\frac{2}{p}} \sum _{j=1}^k \mu ^{(1-2\alpha (1-\frac{1}{p} ))j}\\&\le C \varepsilon _2|s|^{1-\frac{1}{p}}\mu ^{2\alpha k(1-\frac{1}{p})-(1-2\alpha + \frac{2}{p})}. \end{aligned}$$

*Step 3: Decay of a modified excess of* $\theta _0$. *We claim that for every* $r\in (0, \mu ^2)$

43 $$\begin{aligned} \bigg (\, \fint _{- r^{\frac{p}{p-1}}}^0 \fint _{B_{\frac{r}{4}}} |\theta _0 - (\theta _0)_{B_{\frac{r}{4}}\times (- r^\frac{p}{p-1},0]} |^p \, dy \, ds\bigg )^\frac{1}{p} \le 8C_1^{-1} \, \mu ^{-2} r^{\gamma -\left[ \frac{1-2\alpha }{p-1} +\frac{2\alpha }{p(p-1)}\right] } \varepsilon _2.\nonumber \\ \end{aligned}$$

Observe that by the scaling of the excess $\mu ^{2\alpha -1} E^S(\theta _k;\mu )= E^S(\theta _{k, \mu };1)$ and by (42)

$$\begin{aligned} \theta _{k, \mu }(y,s)= \theta _{0, \mu ^{k+1}}(y+ \mu ^{-(k+1)} r_k(\mu ^{2\alpha }s), s). \end{aligned}$$

We introduce the set

$$\begin{aligned} I_{k+1}:=\Big (-\mu ^{(k+1)((1-2\alpha )\frac{p}{p-1} + \frac{2\alpha }{p-1})}, 0\Big ]. \end{aligned}$$

If $s \in I_{k+1}$ we can ensure, by an appropriate choice of $\varepsilon _2$, that $r_k(\mu ^{2\alpha } s) \in B_{3\mu ^{k+1}/4}.$ Indeed

$$\begin{aligned} |r_k(\mu ^{2\alpha } s) |\le C \varepsilon _2 \mu ^{2\alpha (k+1)(1-\frac{1}{p})-(1-2\alpha + \frac{2}{p})} \mu ^{(k+1)(1-2\alpha )} \mu ^{(k+1) \frac{2\alpha }{p}}= C \varepsilon _2 \mu ^{k+1} \mu ^{-(1-2\alpha + \frac{2}{p})}, \end{aligned}$$

for $s \in I_{k+1}$ by Step 2. It is thus enough to choose $\varepsilon _2$ (if necessary) even smaller, or more precisely, we set

$$\begin{aligned} \varepsilon _2 := \min \left\{ {\bar{\varepsilon }}_2, \frac{3}{4}C^{-1}\mu ^{(1-2\alpha + \frac{2}{p})} \right\} . \end{aligned}$$

We now estimate, by adding and substracting $(\theta _{k,\mu })_{Q_1}$ and Hölder

$$\begin{aligned}&\bigg (\, \fint _{I_{k+1}} \fint _{B_{\frac{1}{4}} } |\theta _{0,\mu ^{k+1}}(y,s) - (\theta _{0,\mu ^{k+1}})_{B_\frac{1}{4}\times I_{k+1} } |^p \bigg )^\frac{1}{p} \\&\quad \le 2 \bigg (\, \fint _{I_{k+1}} \fint _{B_{\frac{1}{4}}} |\theta _{0,\mu ^{k+1}}(y,s) - (\theta _{k,\mu })_{Q_1} |^p \, dy \, ds \bigg )^\frac{1}{p}. \end{aligned}$$

Since $\mu ^{-(k+1)}r_k(\mu ^{2\alpha }s) \in B_{3/4}$ we have $B_{1/4} \subseteq \mu ^{-(k+1)} r_k(\mu ^{2\alpha }s)+B_1$ as long as $s\in I_{k+1}$, so that

$$\begin{aligned}&\bigg (\,\fint _{I_{k+1}} \fint _{B_\frac{1}{4}} |\theta _{0,\mu ^{k+1}}(y,s) - (\theta _{k, \mu } )_{Q_1} |^p \bigg )^\frac{1}{p}\\&\quad \le 4^\frac{2}{p} \bigg (\,\fint _{I_{k+1}} \fint _{\mu ^{-(k+1)} r_k(\mu ^{2\alpha }s)+B_1} |\theta _{0,\mu ^{k+1}}(y,s) - (\theta _{k, \mu })_{Q_1} |^p \, dy \, ds \bigg )^\frac{1}{p} \\&\quad = 4^{\frac{2}{p}} \bigg (\,\fint _{I_{k+1}} \fint _{B_1} |\theta _{0,\mu ^{k+1}}(y + \mu ^{-(k+1)} r_k(\mu ^{2\alpha }s),s) - (\theta _{k, \mu })_{Q_1} |^p \, dy \, ds \bigg )^\frac{1}{p} \ \\&\quad \le 4^{\frac{2}{p}} |I_{k+1} |^{-\frac{1}{p}} \bigg (\, \fint _{Q_1} |\theta _{k, \mu } (y, s) - (\theta _{k, \mu })_{Q_1} |^p \, dy \, ds \bigg )^\frac{1}{p} \\&\quad = 4^{\frac{2}{p}} |I_{k+1} |^{-\frac{1}{p}} E^S(\theta _{k, \mu };1) = 4^{\frac{2}{p}} |I_{k+1} |^{-\frac{1}{p}} \mu ^{2\alpha -1} E^S(\theta _k;\mu ). \end{aligned}$$

Combining the previous inequality with Step 1 and observing that $\mu ^{(k+1)2\alpha } I_{k+1}= \big (- \mu ^{(k+1)\frac{p}{p-1}},0\big ]$, we deduce that for $k \ge 1$

$$\begin{aligned}&\bigg (\, \fint _{\mu ^{(k+1)2\alpha } I_{k+1}} \fint _{B_{\frac{1}{4} \mu ^{k+1}}} |\theta _0(y,s) - (\theta _0)_{B_{\frac{1}{4}\mu ^{k+1}} \times \mu ^{(k+1)2\alpha } I_{k+1} } |^p \ dy \, ds\bigg )^\frac{1}{p}\\&\quad = \mu ^{(k+1)(1-2\alpha )}\bigg (\, \fint _{I_{k+1}} \fint _{B_{\frac{1}{4}} } |\theta _{0,\mu ^{k+1}}(y,s) - (\theta _{0,\mu ^{k+1}})_{B_\frac{1}{4}\times I_{k+1}} |^p \bigg )^\frac{1}{p} \\&\quad \le 4 C_1^{-1} |I_{k+1} |^{-\frac{1}{p}} \mu ^{(k+1)\gamma } \varepsilon _2 = 4 C_1^{-1} \mu ^{(k+1)\left( \gamma -\left[ \frac{1-2\alpha }{p-1} +\frac{2\alpha }{p(p-1)}\right] \right) } \varepsilon _2. \end{aligned}$$

This gives (43) for $r= \mu ^{k+1}$ for some $k\ge 1.$ For $r\in (\mu ^{k+2}, \mu ^{k+1})$ instead, we observe that

$$\begin{aligned}&\bigg (\, \fint _{- r^{\frac{p}{p-1}}}^0 \fint _{B_{\frac{r}{4}}} |\theta _0 - (\theta _0)_{B_{\frac{r}{4}}\times (- r^\frac{p}{p-1},0]} |^p \, dy \, ds\bigg )^\frac{1}{p} \\&\quad \le 2 \left( \frac{\mu ^{k+1}}{r}\right) ^{{\frac{1}{p}}(2+ \frac{p}{p-1})}\bigg (\, \fint _{- \mu ^{(k+1)\frac{p}{p-1}}}^0 \fint _{B_{\frac{1}{4} \mu ^{k+1}}} |\theta _0 - (\theta _0)_{B_{\frac{1}{4} \mu ^{k+1}}\times (- \mu ^{(k+1)\frac{p}{p-1}},0]} |^p \, dy \, ds\bigg )^\frac{1}{p} \\&\quad \le 8C_1^{-1} \, \left( \frac{\mu ^{k+1}}{r}\right) ^{\frac{1}{p}(2+ \frac{p}{p-1})} \mu ^{(k+1)\left( \gamma -\left[ \frac{1-2\alpha }{p-1} +\frac{2\alpha }{p(p-1)}\right] \right) } \varepsilon _2\\&\quad \le 8C_1^{-1} \, \mu ^{-2} r^{\left( \gamma -\left[ \frac{1-2\alpha }{p-1} +\frac{2\alpha }{p(p-1)}\right] \right) }\varepsilon _2. \end{aligned}$$

*Step 4: Decay of a modified excess of* $\theta$. *There exists a* $r_0=r_0(\Vert u\Vert _{L^{p+1}(Q_{3/2})})>0$ *such that for every* $r\in (0, r_0)$ *and for every* $(x,t) \in Q_1$

$$\begin{aligned} \bigg (\, \fint _{t- r^{\frac{p}{p-1}}}^t \fint _{B_{\frac{r}{8}}(x)} |\theta - (\theta )_{B_{\frac{r}{8}}(x)\times (t- r^\frac{p}{p-1},t]} |^p \, dy \, ds\bigg )^\frac{1}{p} \le 32 \, \mu ^{-2} r^{\gamma -\left[ \frac{1-2\alpha }{p-1} +\frac{2\alpha }{p(p-1)}\right] } \varepsilon _2. \end{aligned}$$

Since by Theorem 3.2$u\in L^\infty ([-(3/2)^{2\alpha }, 0], {BMO}({\mathbb {R}}^2))\,,$ we have $u \in L^q_{loc}({\mathbb {R}}^2 \times [-(3/2)^{2\alpha }, 0])$ for any $q \in [1, \infty ).$ Fix $(x,t) \in Q_1$. As long as $x_0(s) \in B_{1/4}$ and $|s |< \frac{1}{5}\,,$ we have the estimate

44 $$\begin{aligned} |x_0(s) |\le |s |^{1-\frac{1}{p+1}} \Vert u\Vert _{L^{p+1}(Q_{3/2})}. \end{aligned}$$

In particular for $0 \le |s |^\frac{p}{p+1} \le \min \left\{ \frac{1}{4} \Vert u\Vert _{L^{p+1}(Q_{3/2})}^{-1}, 5^{-\frac{p}{p+1}}\right\}$ the estimate (44) holds. Let now

$$\begin{aligned} r_0:= \min \bigg \{\mu ^2, \bigg ( \frac{1}{4} \Vert u\Vert _{L^{p+1}(Q_{3/2})}^{-1} \bigg )^{1- \frac{1}{p^2}}, \bigg ( \frac{1}{8} \Vert u\Vert _{L^{p+1}(Q_{3/2})}^{-1} \bigg )^{p^2-1} \bigg \}. \end{aligned}$$

Recalling that $\mu ^2 \le \frac{1}{64}\,,$ we observe that for all $r \in (0, r_0)$, $(x,t) \in Q_1$ and $s\in (-r^\frac{p}{p-1}, 0]$ (44) holds and we have

45 $$\begin{aligned} |x_0(s) |\le \Vert u\Vert _{L^{p+1}(Q_{3/2})} r^\frac{p^2}{p^2-1} \le r \left( \Vert u\Vert _{L^{p+1}(Q_{3/2})} r_0^\frac{1}{p^2-1}\right) \le \frac{r}{8}. \end{aligned}$$

Hence we can estimate by the triangular inequality and Hölder, by (45) and by Step 3

$$\begin{aligned}&\bigg (\,\fint _{t-r^\frac{p}{p-1}}^t \fint _{B_\frac{r}{8}(x)} |\theta - (\theta )_{B_\frac{r}{8}(x) \times (t-r^\frac{p}{p-1}, t]} |^p \,dy \, ds \bigg )^\frac{1}{p} \\&\quad \le 2 \bigg (\,\fint _{-r^\frac{p}{p-1}}^0 \fint _{B_\frac{r}{8}(x)} |\theta (y,s+t)- (\theta _0)_{B_\frac{r}{4} \times (r^\frac{p}{p-1}, 0]} |^p \,dy \, ds \bigg )^\frac{1}{p} \\&\quad \le 4 \bigg (\,\fint _{-r^\frac{p}{p-1}}^0 \fint _{B_\frac{r}{4}(x + x_0(s))} |\theta (y,s+t)- (\theta _0)_{B_\frac{r}{4} \times (r^\frac{p}{p-1}, 0]} |^p \,dy \, ds \bigg )^\frac{1}{p} \\&\quad \le 32\, \mu ^{-2} r^{\gamma - \left[ \frac{1-2\alpha }{p-1} + \frac{2\alpha }{p(p-1)}\right] } \varepsilon _2. \end{aligned}$$

*Step 5: By Campanato’s Theorem, we deduce that* $\theta$ *is Hölder continuous in* $Q_1$. By a variant of Campanato’s Theorem [18, Theorem 2.9.], we deduce from Step 4 that (37) holds. Indeed, observe that the sets $B_r(x) \times (t-r^{p/(p-1)}, t]$ are nothing else but balls with respect to the metric $d((x,t), (y,s)):= \max \{ |x-y |, |t-s |^{(p-1)/p} \}$ on spacetime where in time, as usual for parabolic equations, we only look at backward-in-time intervals. The proof of this version of Campanato’s Theorem follows, for instance, line by line [28, Theorem 1] when replacing the parabolic metric by *d*.

$$\square $$

## $\varepsilon$-Regularity Results and Proof of Theorem 1.3

In this section we prove some $\varepsilon$-regularity results, including Theorem 1.3 and its more precise version in Corollary 6.6, by meeting the smallness requirement of Theorem 5.3. As a first result, we deduce in Corollary 6.1 an $\varepsilon$-regularity criterion in terms of a spacetime integral of $\theta$ and *u* that constitutes an analogue of Scheffer’s Theorem [27] for the Navier-Stokes system. As in the case of Navier-Stokes, it implies that the singular set of suitable weak solutions is compact in spacetime (see Step 1 in the proof of Theorem 1.1). In the context of the SQG equation though, in contrast to Navier-Stokes, Corollary 6.1 cannot be used to obtain their almost everywhere smoothness (or any estimate on the dimension of the singular set): The fact that the $L^\infty$-norm is a controlled quantity necessitates to rely on spacetime integrals of derivatives of $\theta$ to show local smoothness. In order to pass from Theorem 5.3 to an $\varepsilon$-regularity criterion involving only fractional space derivatives of $\theta \,,$which are globally controlled through the energy, we need to overcome the following difficulties:

- The excess $E^S\,$ related to $\theta$ involves the spacetime average of $\theta .$ In particular, in order to use a standard Poincaré inequality (10) to pass to a differential quantity, we would need some fractional differentiability in time too. Using the parabolic structure of the equation, we will be able to circumvent this and to establish in Lemma 6.2 a Poincaré inequality which is nonlinear but involves only fractional space derivatives.
- The $\varepsilon$-regularity criterion of Theorem 5.3 features the composition of $\theta$ with the flow $x_0\,,$ so that we need some control on the tilting effect of the flow. We will see that at scale *r*, the flow shifts the center of the excess in space by at most $r^{2\alpha - 2/q} \Vert u\Vert _{L^\infty L^q}\,$ (see (55)). As a consequence, at scale *r*, all quantities related to the excess of $\theta$ will no longer live on parabolic cylinders but rather on modified cylinders ${\mathcal {Q}}(x,t;r)$ in spacetime, approximately of radius $r^{2\alpha }$ in time and $r^{2\alpha -2/q}$ in space. Morally $q= \infty$; however, since the Riesz-transform is bounded from $L^\infty \rightarrow {BMO}$ and not from $L^\infty \rightarrow L^\infty$, we introduce the parameter *q* which should be thought to be arbitrarily large.
- We set the excess in $L^p$ for $p > \frac{1+\alpha }{\alpha }$ in order to gain the compactness of the $(p-1)$-energy inequality. In order to exploit the $L^2 W^{\alpha ,2}$-control given by the energy via the nonlinear Poincaré inequality described in the first point, we are lacking some higher integrability in time. We bypass this issue by factoring out $p-2$ powers of $\theta$ in $L^\infty$.

### An Analogue of Scheffer’s Theorem

We provide a first $\varepsilon$-regularity result featuring spacetime integrals of $\theta$ and *u*. Observe that in agreement with the previous discussion, smooth solutions of (1)–(3) do, in general, not verify the $\varepsilon$-regularity criterion (46) at any small scale.

#### Corollary 6.1

Given $\alpha \in (\frac{1}{4}, \frac{1}{2})$ there exists $\varepsilon =\varepsilon (\alpha )>0$ such that if $p=p(\alpha ):= \frac{6}{4\alpha -1}$ and $\theta$ is a suitable weak solution of (1)–(3) on ${\mathbb {R}}^2 \times (t-(4r)^{2\alpha }, t+ r^{2\alpha }/4]$ satisfying

46 $$\begin{aligned} \frac{1}{r^{(1-2\alpha )p + 2+2\alpha }} \int _{Q_{4r} (x, t + r^{2\alpha }/4)} \left( {{\mathcal {M}}}\theta ^2 \right) ^{\frac{p}{2}}(z,s) + |u |^p(z,s) \, dz \, ds<\varepsilon \,, \end{aligned}$$

then $\theta \in C^{\frac{1}{2}}\left( Q_r (x, t + r^{2\alpha }/4)\right) .$ In particular, $\theta$ is smooth in the interior of $Q_{r/2}(x, t + r^{2\alpha }/4)\supseteq B_{r/2}(x) \times (t-r^{2\alpha }/4, t + r^{2\alpha }/4)$ and hence (*x*, *t*) is a regular point.

#### Proof of Corollary 6.1

Let $\alpha$ and *p* as in the statement. By translation and scaling invariance, we can assume w.l.o.g. that $(x, t + r^{2\alpha }/4)=(0,0)$ and $r=1\,,$ so that we assume that

$$\begin{aligned} \int _{Q_4} ({\mathcal {M}} \theta ^2)^\frac{p}{2}(x,t) + |u |^p(x,t)\, dx \, dt \le \varepsilon . \end{aligned}$$

for an $\varepsilon$ yet to be chosen small enough. We observe that $p > \max \{\frac{1+\alpha }{\alpha }, \frac{2\alpha }{\sigma } \}\,$ for any $\sigma > \frac{1}{6}.$ We define accordingly $\sigma := \frac{4\alpha }{3} + \frac{1}{6}$ and $\gamma :=\frac{2\alpha }{3}+\frac{1}{3}.$ Since $\sigma \in (\frac{1}{6}, 2\alpha )$ and $\gamma \in [1-2\alpha , 2\alpha - \frac{2\alpha }{p}),$ this is an admissible choice of the parameters of Theorem 5.3 and we infer from the latter that $\theta \in C^{\delta , \frac{p-1}{p} \delta }(Q_1)\,,$ with $\delta$ given by (37), provided the smallness requirement (36) holds for any $(x,t) \in Q_1$. Since for $\alpha \in (\frac{1}{4}, \frac{1}{2})$

$$\begin{aligned} \delta =1- 2\alpha +(4\alpha -1) \left( \frac{2}{3}-\frac{4\alpha ^2-7\alpha +3}{3(7\alpha -1)} \right) >1-2\alpha , \end{aligned}$$

we deduce from Lemma B.1 that $\theta$ is smooth in the interior of $Q_{1/2}.$ We observe that for any $(x,t) \in Q_1$ we have $Q_1(x,t) \subseteq Q_4$ and hence

$$\begin{aligned} \bigg ( \, \fint _{Q_1(x,t)} |u |^p \,dz \, ds \bigg )^\frac{1}{p} \le |Q_1 |^{-\frac{1}{p}} \bigg (\int _{Q_4} |u |^p \, dz \,ds \bigg )^\frac{1}{p} \le \varepsilon ^\frac{1}{p}. \end{aligned}$$

Requiring $\varepsilon \le \varepsilon _1^p\,,$ we thus deduce from Lemma  5.1 that $E(\theta _0, u_0; \frac{1}{4}) \le C_1 E(\theta ,u; x,t, 1)$ and hence (36) is enforced for any $(x,t) \in Q_1$ if

47 $$\begin{aligned} \sup _{(x,t) \in Q_1} \, E(\theta ,u; x,t, 1) \le C_1^{-1} \varepsilon _2\,, \end{aligned}$$

where $\varepsilon _2>0$ is given by Theorem 5.3. Using $\theta ^2 \le {\mathcal {M}} \theta ^2$ pointwise almost everywhere, we have

$$\begin{aligned} E^S(\theta ; x, t, 1) + E^V(u; x, t, 1) \le 2 \left( \int _{Q_4} |\theta |^p \, dz \,ds \right) ^\frac{1}{p}+ 2 \left( \int _{Q_4} |u |^p \, dz \, ds \right) ^\frac{1}{p} \le 4\varepsilon ^\frac{1}{p}. \end{aligned}$$

As for the non-local part of the excess, we estimate, using again $\theta ^2 \le {\mathcal {M}} \theta ^2$ almost everywhere,

$$\begin{aligned} E^{NL}(\theta ; x, t, 1)&\le \Bigg ( \,\fint _{t-1}^{t} \sup _{R \ge \frac{1}{4}} \frac{1}{R^{\sigma p}} \bigg (\, \fint _{B_R(x)} \theta ^2 \, dz\bigg )^\frac{p}{2} \, ds \Bigg )^\frac{1}{p} + C|(\theta )_{Q_1(x, t)} |\\&\le C \Bigg (\, \fint _{t-1}^{t} \sup _{R \ge \frac{1}{4}} \frac{1}{R^{\sigma p}} \bigg ( \int _{B_\frac{1}{4}(x)}\fint _{B_{2R}(z')} \theta ^2(z,s) \, dz \, dz' \bigg )^\frac{p}{2} \, ds \Bigg )^\frac{1}{p} \\&\quad + C \bigg (\int _{Q_4} |\theta |^p \, dz \, ds \bigg )^\frac{1}{p} \\&\le C \Bigg ( \fint _{t-1}^{t} \sup _{R \ge \frac{1}{4}} \frac{1}{R^{\sigma p}} \bigg ( \int _{B_\frac{1}{4}(x)}{\mathcal {M}}\theta ^2(z', s) \, dz' \bigg )^\frac{p}{2} \, ds \Bigg )^\frac{1}{p} + C \varepsilon ^\frac{1}{p} \le C^{NL} \varepsilon ^\frac{1}{p}. \end{aligned}$$

Hence we reach (47) by choosing $\varepsilon \le \min \left\{ ( \varepsilon _1)^p, (4+ C^{NL})^{-1} C_1^{-1}\varepsilon _2)^p \right\} .$ $\square$

### Nonlinear Poincaré Inequality of Parabolic Type

We introduce the following scaling-invariant quantity which should be understood as a localized version of the dissipative part of the energy:

$$\begin{aligned} {\mathcal {E}}(\theta ; x,t,r)&:= \frac{1}{r^{2(1-2\alpha ) + 2}} \int _{Q_r^*(x,t)} y^b |{\overline{\nabla }} \theta ^* |^2(z,y,s)\, dz \, dy \, ds. \end{aligned}$$

The following Lemma and its proof is inspired by [32], where a parabolic Poincaré inequality is obtained for the classical, linear heat equation, and by [26], where a nonlinear Poincaré inequality of similar nature is also crucially used in a $\varepsilon$-regularity result.

#### Lemma 6.2

(Nonlinear Poincaré inequality of parabolic type)

Let $\alpha \in (0,1).$ There exists a constant $C=C(\alpha )\ge 1$ such that the following holds: Let $Q_r(x,t) \subseteq {\mathbb {R}}^2 \times (0, \infty )$ and let $(\theta , u)$ be a Leray–Hopf weak solution of (1)–(2). We assume that the velocity field is obtained by

$$\begin{aligned} u(z,s) = {\mathcal {R}}^\perp \theta (z,s) + f(s) \, \end{aligned}$$

for some $f \in L^1_{loc}({\mathbb {R}})$ and that it satisfies $\left[ u(s)\right] _{B_r(x)} = 0$ for all $s \in [t-(2r)^{2\alpha }, t]$. Then we have for any $q \in \big [2, \frac{2}{1-\alpha } \big ]$ that

$$\begin{aligned}&\frac{1}{r^{(1-2\alpha ) + \frac{2}{q} + \alpha }} \Bigg (\int _{t- r^{2\alpha }}^{t} \bigg (\int _{B_r(x)} |\theta (z,s) - (\theta )_{Q_r(x,t)} |^q \, dz \bigg )^\frac{2}{q} \, ds \Bigg )^\frac{1}{2} \le C \big ( {\mathcal {E}}(\theta ; x,t, 3r)^\frac{1}{2} \\&\quad +\bigg ( \frac{1}{r^{2(1-2\alpha )+2+2\alpha }} \int _{Q_{2r}(x,t)} |u (z,s)- [u(s)]_{B_{2r}(x)} |^2 \, dz \, ds \bigg )^\frac{1}{2} {\mathcal {E}}(\theta ; x,t, 3r)^\frac{1}{2} \big ). \end{aligned}$$

#### Proof

By translation and scaling invariance (with respect to (4)), we may assume $(x,t)=(0,0)\,$ and $r=1.$

*Step 1: By means of the weighted Poincaré inequality* (11), *we reduce the Lemma to an estimate on weighted space averages computed at two different times. To this aim, let* $\omega \in C^\infty _{c}({\mathbb {R}}^{3}_+ )$ *be a weight such that* $\omega |_{y=0}$ *is a radial non-increasing function,* $0 \le \omega \le 1$ *and* $\omega \equiv 1$ *on* ${\overline{B}}_{1} \times [0, 1]$ *and* $\omega \equiv 0$ *outside* $B_{2} \times [0, 2).$

We estimate for fixed time

$$\begin{aligned} \left( \int _{B_1} |\theta (x,t) - (\theta )_{Q_1} |^q \, dx \right) ^\frac{1}{q}&\le \left( \int _{B_{2}} |\theta (x,t) -[\theta (t)]_{\omega |_{y=0}, B_2} |^q \omega (x,0) \,dx \right) ^\frac{1}{q} \\&\quad + \pi ^\frac{1}{q} \left( \big | [\theta (t)]_{\omega |_{y=0}, B_2}-(\theta )_{\omega |_{y=0}, Q_2} \big |+ \big | (\theta )_{\omega |_{y=0}, Q_2} -(\theta )_{Q_1} \big | \right) , \end{aligned}$$

where we used $\omega (\cdot , 0) \equiv 1$ on ${\overline{B}}_1.$ Reusing this fact and Hölder, we bound the last term by

$$\begin{aligned}&\big | (\theta )_{\omega |_{y=0}, Q_2} - (\theta )_{Q_1}\big | \le \left( \int _{-1}^0 \left( \fint _{B_1} |\theta - (\theta )_{\omega |_{y=0}, Q_2} |^q \,dx \right) ^\frac{2}{q} \, dt\right) ^\frac{1}{2} \\&\quad \le \pi ^{-\frac{1}{q}} \left( \int _{-1}^0 \left( \int _{B_{2}} |\theta - [\theta (t)]_{\omega |_{y=0}, B_2} |^q \omega (x,0) \, dx \right) ^\frac{2}{q} \, dt \right) ^\frac{1}{2} \\&\quad + \left( \int _{-1}^0 |[\theta (t)]_{\omega |_{y=0}, B_2}- (\theta )_{\omega |_{y=0}, Q_2} |^2 \, dt\right) ^\frac{1}{2}, \end{aligned}$$

so that we deduce by the weighted Poincaré inequality (11)

$$\begin{aligned}&\left( \int _{-1}^0 \left( \int _{B_1} |\theta (x,t)- (\theta )_{Q_1} |^q \, dx \right) ^\frac{2}{q} \, dt \right) ^\frac{1}{2} \le C \Bigg [ \left( \int _{-1}^0 [\theta (t)]_{W^{\alpha , 2}(B_{2})}^2 \, dt \right) ^\frac{1}{2} \\&\quad + \left( \int _{-1}^0 |[\theta (t)]_{\omega |_{y=0}, B_2}- (\theta )_{\omega |_{y=0},Q_2} |^2 \, dt \right) ^\frac{1}{2}\Bigg ]. \end{aligned}$$

The first term on the right-hand side can be expressed in terms of the extension by (14). Since the weight $\omega$ is independent of time, the second term can be estimated by

$$\begin{aligned}&\left( \int _{-1}^0 |[\theta (t)]_{\omega |_{y=0}, B_2}- (\theta )_{\omega |_{y=0}, Q_2} |^2 \, dt \right) ^\frac{1}{2}\\&\quad \le \left( \int _{-2^{2\alpha }}^0 \int _{-2^{2\alpha }}^0 |[\theta (t)]_{\omega |_{y=0}, B_2} - [\theta (s)]_{\omega |_{y=0}, B_2} |^2\, ds\, dt \right) ^\frac{1}{2}. \end{aligned}$$

*Step 2: We use the equation to estimate the difference of two weighted space averages computed at different times.*

We use the weak formulation (22) of the equation with time-independent test function $\varphi (x,y):= {{\,\mathrm{sgn}\,}}([\theta (t)]_{\omega |_{y=0}, B_2} -[\theta (s)]_{\omega |_{y=0}, B_2}) \omega (x, y).$ We estimate the right-hand side of (22) from below and the left-hand side from above for $s,t \in [-2^{2\alpha }, 0]$. As for the lower bound, we have

$$\begin{aligned} \int (\theta (x,t) - \theta (x,s)) \varphi (x, 0) \, dx&=\big | [\theta (t)]_{\omega |_{y=0}, B_2} -[\theta (s)]_{\omega |_{y=0}, B_2}\big | \int _{B_{2}} \omega (x,0) \, dx \nonumber \\&\ge \pi \big | [\theta (t)]_{\omega |_{y=0}, B_2} -[\theta (s)]_{\omega |_{y=0}, B_2}\big | \end{aligned}$$

since $\omega (\cdot , 0) \equiv 1$ on ${\overline{B}}_1.$ As for the right-hand side, we estimate by Hölder

$$\begin{aligned} \bigg |\int _{s}^t \int _{{\mathbb {R}}^3_+} y^b {\overline{\nabla }} \theta ^* \cdot {\overline{\nabla }} \varphi \, dx \, dy \, d\tau \bigg |&\le 2^{1-\alpha } \Vert \omega \Vert _{C^1} |Q_{2} |^\frac{1}{2} \left( \int _{Q_2^*} y^b |{\overline{\nabla }} \theta ^*|^2 \, dx \, dy \, d\tau \right) ^\frac{1}{2}. \end{aligned}$$

Since *u* is divergence-free and $[u(\tau )]_{B_1}=0$ for $\tau \in [ -2^{2\alpha }, 0]$ by assumption, we can estimate the nonlinear term by Hölder and the Poincaré inequality (10) combined with (14)

$$\begin{aligned} \left|\int _{s}^t \int u\theta \cdot \nabla \varphi |_{y=0} \, dx \, d \tau \right|&=\left|\int _{s}^t \int (u- [u(\tau )]_{B_1})(\theta - [\theta (\tau )]_{B_2}) \cdot \nabla \varphi |_{y=0} \, dx \, d \tau \right|\\&\le \Vert \omega \Vert _{C^1} \left( \int _{Q_{2}} |u- [u(\tau )]_{B_1} |^2 \, dx \, d\tau \right) ^\frac{1}{2} \\&\quad \left( \int _{Q_{2} } |\theta - [\theta (\tau )]_{B_2} |^2 \, dx \, d\tau \right) ^\frac{1}{2} \\&\le C \left( \int _{Q_{2}} |u- [u(\tau )]_{B_2} |^2 \, dx \, d\tau \right) ^\frac{1}{2} \\&\quad \left( \int _{Q_3^*} y^b |\overline{\nabla } \theta ^* |^2 \, dx \, dy \, d\tau \right) ^\frac{1}{2}. \end{aligned}$$

Collecting terms, we have for almost every $s,t \in [-2^{2\alpha }, 0]$ that

$$\begin{aligned}&\big | [\theta (t)]_{\omega |_{y=0}, B_2} - [\theta (s)]_{\omega |_{y=0}, B_2} \big | \lesssim \Bigg (\int _{Q_{3}^*} y^b |{\overline{\nabla }} \theta ^* |^2 \, dx \, dy \, d \tau \Bigg )^\frac{1}{2}\\ {}&\quad \Bigg (1 + \bigg (\int _{Q_{2}} |u - [u(\tau )]_{B_2} |^2 \,dx \, d\tau \bigg )^\frac{1}{2} \Bigg ) \, . \end{aligned}$$

Combining this estimate with Step 1, we conclude. $\square$

### The Non-local Part of Excess

We recall from the proof of Proposition 4.1 that the excess related to the velocity can be estimated in terms of $\theta .$ More precisely, we have the following:

#### Lemma 6.3

Let $\alpha \in (\frac{1}{4}, \frac{1}{2}),$ $Q_r(x,t) \subseteq {\mathbb {R}}^2 \times (0, \infty )$ and $\theta \in L^p({\mathbb {R}}^2 \times [t-(3r/2)^{2\alpha }, t]).$ Consider a velocity field of the form $u(z,s)= {\mathcal {R}}^\perp \theta (z,s) + f(s)$ for some $f \in L^1_{loc}({\mathbb {R}}).$ There exists $C=C(p)\ge 1$ such that

$$\begin{aligned} E^V(u; x, t, r) \le C \Bigg ( \bigg ( \, \fint _{Q_{\frac{3r}{2}}(x,t)} |\theta (z,s){-} [\theta (s)]_{B_{\frac{3r}{2}}(x)} |^p \, dz \,ds \bigg )^\frac{1}{p}{+} E^{NL}(\theta ; x, t, \frac{3}{2} r) \Bigg ). \end{aligned}$$

After reducing the Lemma to $r=1$ and $(x,t)=(0,0)$, the proof follows line-by-line the estimate of $E^V$ in the proof of Proposition 4.1. We now bound the quantity $E^{NL}$ in terms of a variant of the sharp maximal function introduced in Sect. 2.6.

#### Lemma 6.4

Let $\alpha \in (\frac{1}{4}, \frac{1}{2})\,$, $Q_r(x,t) \subseteq {\mathbb {R}}^2 \times (0, \infty )\,$ and $\theta \in L^\infty ({\mathbb {R}}^2 \times [t-r^{2\alpha }, t]) \cap L^2([t-r^{2\alpha }, t], W^{\alpha ,2}({\mathbb {R}}^2)).$ If $(p, q, \sigma ) \in (\frac{1+\alpha }{\alpha }, \infty ) \times [2, \infty ) \times (0, 2\alpha )$ satisfy the admissibility criterion

48 $$\begin{aligned} \sigma p-(2+ 2\alpha ) - \frac{2}{q^2-1} \ge 0, \end{aligned}$$

then there exists a constant $C= C(p)\ge 1$ such that

$$\begin{aligned}&\frac{1}{r^{p(1-2\alpha )}}E^{NL}(\theta ; x, t, r)^p \nonumber \\&\quad \le C \frac{\Vert \theta \Vert _{L^\infty ([t-r^{2\alpha }, t] \times {\mathbb {R}}^2)}^{p-2}}{r^{p(1-2\alpha )+2}} \int _{t-r^{2\alpha }}^{t} \int _{B_\frac{r}{4}(x)} |\theta ^\#_{\alpha , q}(z,s) |^{1+\frac{1}{q^2-1}}\, dz \,ds. \end{aligned}$$

#### Proof

By translation and scaling invariance, we may assume $(x,t)=(0,0)$ and $r=1$. By factoring out $p-2$ powers in $L^\infty \,,$ by adding and subtracting $[\theta (s)]_{B_{1/4}}$ for fixed time *s* and radius *R*, and by reabsorbing $|[\theta (s)]_{B_1}- [\theta (s)]_{B_{1/4}} |$ in the supremum, we have

$$\begin{aligned} E^{NL}(\theta ; 1)^p&\le (2 \Vert \theta \Vert _{L^\infty ({\mathbb {R}}^2 \times [-1, 0])})^{p-2} \, \\&\quad \int _{-1}^0 \sup _{R \ge \frac{1}{4}} \left( \frac{1}{R} \right) ^{\sigma p} \left( \, \fint _{B_R} |\theta (x,s)- [\theta (s)]_{B_1}|^\frac{3}{2} \, dx \right) ^{\frac{4}{3} } \, ds \\&\lesssim \Vert \theta \Vert _{L^\infty ({\mathbb {R}}^2 \times [-1, 0])}^{p-2} \, \int _{-1}^0 \sup _{R \ge \frac{1}{4}} \left( \frac{1}{R} \right) ^{\sigma p} \left( \,\fint _{B_R} |\theta (x,s)- [\theta (s)]_{B_\frac{1}{4}}|^\frac{3}{2} \, dx \right) ^\frac{4}{3} \, ds. \end{aligned}$$

We estimate the argument of the supremum for fixed time *s* and radius $R \ge \frac{1}{4} \,$ by the triangular inequality and Hölder

49 $$\begin{aligned} \bigg (\, \fint _{B_R} |\theta - [\theta (s)]_{B_\frac{1}{4}} |^\frac{3}{2} \, dx \bigg )^\frac{4}{3}&\le 2 \Bigg ( \bigg ( \,\fint _{B_R} |\theta - [\theta (s)]_{B_R} |^{2(1- 1/q^2)} \, dx \bigg )^{1+ \frac{1}{q^2-1}} \nonumber \\&\quad + |[\theta (s)]_{B_\frac{1}{4}}- [\theta (s)]_{B_R} |^2 \Bigg ) \nonumber \\&\le 4 \left( (4R)^2 \fint _{B_R} |\theta - [\theta (s)]_{B_R} |^{2(1- 1/q^2)} \, dx \right) ^{1+ \frac{1}{q^2-1}}. \end{aligned}$$

For $z \in B_{1/4}$ it holds $B_R \subseteq B_{2R}(z)$, so that by the triangular inequality and by averaging over $z \in B_{1/4}\,,$ we have

50 $$\begin{aligned} \fint _{B_R} |\theta (x,s)- [\theta (s)]_{B_R}|^{2(1-1/q^2)}\, dx&\le 4 \, \, \fint _{B_\frac{1}{4}} \fint _{B_{2R}(z)} |\theta (x,s)- [\theta (s)]_{B_{2R}(z)}|^{2(1-1/q^2)}\, dx \, dz \nonumber \\&\le 32 \, R^{2\alpha (1-1/q^2)}\, \fint _{B_\frac{1}{4}} \theta ^\#_{\alpha , q}(z,s) \, dz. \end{aligned}$$

We combine (49)–(50) and use Hölder to bring the power $1+\frac{1}{q^2-1}$ inside the integral. We obtain

$$\begin{aligned} E^{NL}(\theta ;1) \le C \Vert \theta \Vert _{L^\infty ({\mathbb {R}}^2 \times [-1, 0])}^{p-2} \, \int _{-1}^0 \int _{B_\frac{1}{4}} |\theta ^\#_{\alpha , q}(z,s) |^{1+\frac{1}{q^2-1}} \, dz \, ds \, , \end{aligned}$$

provided

$$\begin{aligned} \sup _{R \ge \frac{1}{4}} \left( \frac{1}{R}\right) ^{\sigma p - (2+2\alpha ) -\frac{2}{q^2-1}} < + \infty . \end{aligned}$$

Observe that this is ensured through (48) and that the supremum can be estimated from above by $4^{p}$ such that the constant of the Lemma depends only on *p*. $\square$

### Proof of the Theorem 1.3

The following Corollary of Theorem 5.3 gives a different version of the $\varepsilon$-regularity criterion in terms of $\theta$ rather than its composition with the flow $\theta _0.$ Theorem 1.3 will be an immediate consequence. To this aim, we introduce the following modified balls and cylinders (backwards and centered in time respectively) which are enlarged in space in accordance with the “intrinsic effect” of the flow (see (55)):

$$\begin{aligned}&{\mathcal {B}}(x;r):= B_{K_q r^{2\alpha - 2/q}}(x) \, \text { and } {\mathcal {B}}^*(x;r):= {\mathcal {B}}(x;r) \times [0, r), \\&{\mathcal {Q}}(x,t;r) := {\mathcal {B}}(x;r)\times (t-r^{2\alpha }, t]\,\text {and}\; {\mathcal {Q}}^*(x,t;r) := {\mathcal {B}}^*(x;r)\times (t-r^{2\alpha }, t]\,,\\&{\mathcal {C}}(x,t;r) := {\mathcal {B}}(x;r)\times (t-r^{2\alpha }, t+r^{2\alpha }) \, \text { and } {\mathcal {C}}^*(x,t;r) := {\mathcal {B}}^*(x;r) \times (t-r^{2\alpha }, t+ r^{2\alpha }), \end{aligned}$$

where

51 $$\begin{aligned} K_q=K_q(u; x,t,r) : = 2 \max \left\{ \Vert u\Vert _{L^\infty ([t-r^{2\alpha }, t+r^{2\alpha }], L^q({\mathbb {R}}^2))}, r^{1-2\alpha + 2/q} \right\} . \end{aligned}$$

To shorten notation, we will often omit the dependence of $K_q$ on *u* and $r\,,$ and whenever $(x,t)=(0,0)\,,$ we will omit to specify the center of the balls and cylinders. The following remark justifies that one should really think of ${\mathcal {B}}(x;r)$ as a enlarged balls of radius $r^{2\alpha - 2/q}.$

#### Remark 6.5

(Upper bound on $K_q$) For $0 < r \le (t/2)^{\frac{1}{2\alpha }}$ by Calderon–Zygmund, Theorem 3.2 and the energy inequality (20) of Leray–Hopf weak solutions

$$\begin{aligned} \Vert u\Vert _{L^\infty ([t-r^{2\alpha }, t+r^{2\alpha }], L^q({\mathbb {R}}^2))} \le C \Vert \theta \Vert _{L^\infty ([t-r^{2\alpha }, t+r^{2\alpha }], L^q({\mathbb {R}}^2))} \le C_2 \Vert \theta _0\Vert _{L^2} t^{-\frac{1}{2\alpha }(1- 2/q)} \end{aligned}$$

such that for $0<r \le r_0:= \min \left\{ (t/2)^{\frac{1}{2\alpha }}, \left( C_2 \Vert \theta _0\Vert _{L^2} t^{-\frac{1}{2\alpha }(1- 2/q)}\right) ^{1/(1-2\alpha + 2/q)} \right\}$ and any $x \in {\mathbb {R}}^2$

$$\begin{aligned} K_q(u; x,t,r) \le 2C_2 \Vert \theta _0\Vert _{L^2} t^{-\frac{1}{2\alpha }(1- 2/q)}. \end{aligned}$$

#### Corollary 6.6

Let $\alpha _0:= \frac{1+\sqrt{33}}{16}$, $\alpha \in [ \alpha _0, \frac{1}{2})\,,$ $q \ge 8 \,$ and $p=p(q):= \frac{1+\alpha }{\alpha } + \frac{1}{q}.$ There exists a universal $\varepsilon =\varepsilon (\alpha )\in (0,1)$ such that the following holds: If $(\theta , u)$ is a suitable weak solution to (1)–(3) on ${\mathbb {R}}^2 \times [t- (4r)^{2\alpha }, t]$ satisfying

52 $$\begin{aligned} \frac{\Vert \theta \Vert _{L^\infty ({\mathbb {R}}^2 \times [t-(4r)^{2\alpha }, t])}^{p-2}}{(4r)^{p(1-2\alpha )+ 2}} \left( \int _{{\mathcal {Q}}^*(x,t;4r)} y^b |{\overline{\nabla }} \theta ^* |^2 \, dz \, dy \,ds + \int _{{\mathcal {Q}}(x,t;4r)} |\theta ^\#_{\alpha ,q} |^{1 + \frac{1}{q^2-1}} \, dz \, ds \right) \le \varepsilon \,,\nonumber \\ \end{aligned}$$

then $\theta$ is smooth in the interior of $Q_{r/2}(x,t).$

#### Remark 6.7

(Justification of $\alpha _0$) $\alpha _0$ is the threshold until which both the smallness hypothesis of Corollary 6.6 is verified, at sufficiently small scale, for smooth solutions at any point (*x*, *t*) in spacetime and the dimension estimate of Theorem  1.1 is non-trivial. Indeed, for $\alpha > \alpha _0$ it holds

$$\begin{aligned} \frac{1}{2\alpha } \Big (\frac{1+\alpha }{\alpha }(1-2\alpha )+ 2 \Big ) < 3. \end{aligned}$$

Before proceeding with the proof, let us show that Theorem 1.3 is an immediate consequence of Corollary 6.6.

#### Proof of Theorem 1.3

Let $\alpha \,, p$ and *q* as in the statement and assume that (5) holds. Observe that ${\mathcal {C}}(x,t; r) \supseteq {\mathcal {Q}}(x, t + r^{2\alpha }/16; r).$ By (18) and Hölder we deduce the pointwise estimate

$$\begin{aligned} \theta ^\#_{\alpha , q}(z,s) \le {\mathcal {M}}\big ( \left( D_{\alpha , 2} \,\theta \right) ^{2(1-1/q^2)}\big )(z,s) \le \big [{\mathcal {M}}\big ( \left( D_{\alpha , 2} \,\theta \right) ^2 \big )\big ]^{1-1/q^2}(z,s). \end{aligned}$$

We infer that $\theta$ satisfies (52) at the radius *r*/4 and the point $(x, t+ r^{2\alpha }/16).$ We deduce from Corollary 6.6 that $\theta$ is smooth in the interior of $Q_{r/8}(x, t+r^{2\alpha }/16)$ which contains the open ball $B_{r/8}(x) \times (t-r^{2\alpha }/16, t+r^{2\alpha }/16).$ $\square$

#### Proof of Corollary 6.6

By translation and scaling invariance, we assume $(x,t)=(0,0)$ and $r=1.$

*Step 1:* *We tune the free parameters* $\sigma$ *and* $\gamma$.

We define $\sigma := 2\alpha - \frac{1}{q^2}.$ Observe that with this choice the triple $(\sigma , p, q)$ satisfies the hypothesis of Lemma 6.4. Indeed, recalling that by assumption $\alpha \ge \alpha _0 > \frac{2}{5}$, we have for $q \ge 8$ that

$$\begin{aligned} \sigma p - (2+2\alpha ) - \frac{2}{q^2-1} = \frac{1}{q}\left( 2\alpha - \frac{1}{q}\left( \frac{1+\alpha }{\alpha } + \frac{1}{q} +\frac{2q^2}{q^2-1}\right) \right)> \frac{1}{q}\left( 2\alpha - \frac{5}{4}\right) >0. \end{aligned}$$

We introduce also $\gamma :=2\alpha - \frac{4\alpha ^2}{1+\alpha } \in [1-2\alpha , \sigma - 2\alpha /p)$, so that the triple $(\sigma , p, \gamma )$ satisfies the hypothesis of Theorem 5.3. We conclude from the latter that $\theta \in C^{\delta , (1-1/p) \delta }(Q_r) \subseteq L^\infty ( (-1,0); C^{\delta }(B_1))\,,$ with $\delta$ given by (37), provided

53 $$\begin{aligned} \sup _{(x,t) \in Q_1} E\left( \theta _{0}, u_{0}; \frac{1}{4}\right) \le \varepsilon _2\,, \end{aligned}$$

where for $(x,t) \in Q_1$ fixed, we define $\theta _{0}(z,s)= \theta (z+x_0(s)+x, s+t)\,,$ $u_{0}(z,s)=u(z+x_0(s)+x, s+t) - \dot{x}_0(s)$ and $x_0$ is the flow given by Lemma 5.1. We have

$$\begin{aligned} \delta = \gamma - \frac{1}{p-1} \left[ 1-2\alpha + \frac{2\alpha }{p} \right] > 2\alpha -\frac{4\alpha ^2}{1+\alpha } - \alpha \left[ 1-2\alpha + \frac{2\alpha ^2}{1+\alpha } \right] \,, \end{aligned}$$

where the right-hand side exceeds $1-2\alpha$ for $\alpha \ge \sqrt{2}-1$, so in particular for $\alpha \ge \alpha _0.$ We deduce from Lemma B.1 that $\theta$ is smooth in the interior of $Q_{1/2}.$ We are thus left to verify that (53) can be enforced by requiring (52).

*Step 2: We bound the full excess* $\sup _{(x,t) \in Q_1}E(\theta _0, u_0; \frac{1}{4}).$

For $(x,t) \in Q_1\,$ fixed, we estimate by factoring out $p-2$ powers in $L^\infty$, by Lemma 6.2, by Hölder and Young

$$\begin{aligned} E^S\left( \theta _{0}; \frac{1}{4}\right) ^p&\lesssim \Vert \theta _{0}\Vert _{L^\infty \left( Q_\frac{1}{4}\right) }^{p-2} {\mathcal {E}}(\theta _{0}; \frac{3}{4}) + \Vert \theta _{0}\Vert _{L^\infty \left( Q_\frac{1}{4}\right) }^{p-2} {\mathcal {E}}(\theta _{0}; \frac{3}{4}) E^V(u_{0}; \frac{1}{2})^2 \\&\lesssim \Vert \theta _{0}\Vert _{L^\infty ({\mathbb {R}}^2 \times [-1, 0])}^{p-2} {\mathcal {E}}(\theta _{0}; 1) +\left( \Vert \theta _{0}\Vert _{L^\infty ({\mathbb {R}}^2 \times [-1, 0])}^{p-2} {\mathcal {E}}(\theta _{0}; 1) \right) ^{1+\frac{2}{p-2}} \\&\quad + E^V(u_0; \frac{1}{2})^p. \end{aligned}$$

Moreover, by Lemma 6.3 and (10) combined with (14), recalling that $u_{0}={\mathcal {R}}^\perp \theta _{0}- \dot{x}_0(s)$, we have

$$\begin{aligned} E^V(u_{0}; \frac{1}{2})^p&\lesssim \int _{Q_\frac{3}{4}} |\theta _{0} - [\theta _0(s)]_{B_\frac{3}{4}} |^p\, dz \, ds + E^{NL}(\theta _{0}; \frac{3}{4})^p \\&\quad \lesssim \Vert \theta _{0}\Vert _{L^\infty (Q_\frac{3}{4})}^{p-2} {\mathcal {E}}(\theta _{0}; 1) + E^{NL}(\theta _{0}; \frac{3}{4})^p \, . \end{aligned}$$

Collecting terms and applying Lemma 6.4, we deduce that for every $(x,t) \in Q_1$ with a constant $C=C(\alpha )\ge 1$ ( observe that *p* cannot exceed $\frac{1+\alpha }{\alpha }+1$)

54 $$\begin{aligned} E(\theta _{0}, u_{0}; \frac{1}{4})^p \le C \bigg ( \Vert \theta _{0}\Vert _{L^\infty ({\mathbb {R}}^2 \times [-1, 0])}^{p-2}&{\mathcal {E}}(\theta _{0}; 1) +\left( \Vert \theta _{0}\Vert _{L^\infty ({\mathbb {R}}^2 \times [-1, 0])}^{p-2} {\mathcal {E}}(\theta _{0}; 1) \right) ^{1+\frac{2}{p-2}} \nonumber \\&+ \Vert \theta _0\Vert _{L^\infty ({\mathbb {R}}^2 \times [-1, 0])}^{p-2} \int _{-1}^0 \int _{B_\frac{1}{4}} |(\theta _0)^\#_{\alpha , q}|^{1 + \frac{1}{q^2-1}} \, dz \, ds \bigg ). \end{aligned}$$

*Step 3: We estimate the tilting effect of the flow. To this aim, introduce a parameter* $q \ge 8$ (*to ensure that* $2\alpha -2/q\ge 1/4>0$).

For $(x,t) \in Q_1$ and $s \in [-1, 0]$ we have by the definition of the flow (34)

$$\begin{aligned} |\dot{x}_0(s) |\le |B_\frac{1}{4} |^{-\frac{1}{q}} \Vert u\Vert _{L^\infty ( [t-1, t ], L^q({\mathbb {R}}^2))} \le 2 \Vert u\Vert _{L^\infty ([ -4^{2\alpha },0], L^q({\mathbb {R}}^2))}\le K_q(u;4). \end{aligned}$$

Hence the center of the excess in space can be shifted by at most

55 $$\begin{aligned} \sup _{(x,t) \in Q_1} \sup _{s \in [-1, 0]} |x_0(s) |\le K_q(u;4). \end{aligned}$$

Since $\theta _{0}$ is just a spatial translation of $\theta$, we estimate $\Vert \theta _{0}\Vert _{L^\infty ({\mathbb {R}}^2 \times [-1, 0])} \le \Vert \theta \Vert _{L^\infty ({\mathbb {R}}^2 \times [- 4^{2\alpha },0])} \,$ for $(x,t) \in Q_1.$ Recall that the extension is obtained by convolution with a Poisson kernel. Since translation and convolution commute, we have $(\theta _0)^*(z,y,s)= \theta ^*(z+ x_0(s)+x, y,s+t)$ and hence

56 $$\begin{aligned} {\mathcal {E}}(\theta _{0}; 1)&=\int _{Q_1^*} y^b |{\overline{\nabla }} \theta ^* |^2 (z+ x_0(s)+x, y,s+t) \, dz \, dy \, ds \nonumber \\&\le \int _{{\mathcal {Q}}^*(4)} y^b |\overline{\nabla } \theta ^* |^2 (z,y, s) \, dz \, dy \, ds. \end{aligned}$$

We used in the last inequality that for $(x,t) \in Q_1$ it holds $t-1\ge - 4^{2\alpha }$ and

57 $$\begin{aligned} B_1(x) + x_0(s) \subseteq B_{2} + B_{K_q(u;4)} \subseteq \frac{1}{4} {\mathcal {B}}(4) + \frac{1}{4^{2\alpha - 2/q}} {\mathcal {B}}(4) \subseteq {\mathcal {B}}(4) \end{aligned}$$

for any $s \in [-1, 0]$ by (55). As for the remaining term in (54), we observe that $(\theta _0)^\#_{\alpha ,q} (z,s) = \theta ^\#_{\alpha , q}(z+x_0(s)+x, s+t)$ and we reuse (57) to estimate

58 $$\begin{aligned} \int _{-1}^0 \int _{B_\frac{1}{4}} |(\theta _0)^\#_{\alpha , q}|^{1 + \frac{1}{q^2-1}} \, dz \, ds&\le \int _{{\mathcal {Q}}(4)}|\theta ^\#_{\alpha , q}|^{1 + \frac{1}{q^2-1}} \, dz \, ds. \end{aligned}$$

Combining (54), (56) and (58), we reach (53) by requiring (52). $\square$

## The Singular Set and Proof of Theorem 1.1

We recall the box-counting dimension of a (compact) set ${\mathcal {S}} \subseteq {\mathbb {R}}^3\,:$ For every $\delta \in (0,1)$ we denote by $N(\delta )$ the minimal number of sets of diameter $\delta$ needed to cover ${\mathcal {S}}.$ We then define

$$\begin{aligned} \dim _b({\mathcal {S}}) := \limsup _{\delta \rightarrow 0} - \log _\delta ( N(\delta )). \end{aligned}$$

It is well-known that the box-counting dimension controls the Hausdorff dimension $\dim _{\mathcal {H}}\,,$ i.e. $\dim _{\mathcal {H}} {\mathcal {S}} \le \dim _b {\mathcal {S}}.$

### Proof of Theorem 1.1

*Step 1: Fix* $t>0$ *and define* ${\mathcal {S}}:= \mathrm{Sing} \, \theta \cap \left[ {\mathbb {R}}^2 \times [t, \infty ) \right] .$ *We claim tha*t ${\mathcal {S}}$ *is a compact set in spacetime. *

From the definition it is clear that ${\mathcal {S}}$ is closed and we claim that it is also bounded. Indeed, let *p* be as in Corollary 6.1 and let $p' \ge p$. From the maximal function estimate and Calderon–Zygmund

$$\begin{aligned} \int _t^\infty \int ({\mathcal {M}} \theta ^2)^\frac{p'}{2} + |u |^{p'} \, dx \, ds&\lesssim \int _t^\infty \int |\theta |^{p'} \, dx \, ds \lesssim \Vert \theta \Vert _{L^\infty ({\mathbb {R}}^2 \times [t, \infty ))}^{p'-2(1+\alpha )} \Vert \theta \Vert _{L^{2(1+\alpha )}({\mathbb {R}}^2 \times [t, \infty ))}^{2(1+\alpha )} \\&\le C(\Vert \theta _0\Vert _{L^2}) t^{- \frac{p'-2(1+\alpha )}{2\alpha }} \,, \end{aligned}$$

by Theorem 3.2 and the global energy inequality (20) of Leray–Hopf weak solutions. By the absolute continuity of the integral we deduce that for every $\varepsilon >0$ there exists $M=M(\theta , \varepsilon )>0$ and $T^*=T^*(\Vert \theta _0\Vert _{L^2}, \varepsilon )>0$ such that

$$\begin{aligned} \int _t^\infty \int _{{\mathbb {R}}^2 \setminus B_M}({\mathcal {M}} \theta ^2)^\frac{p}{2} + |u |^p \, dx \, ds +\int _{T^*}^\infty \int _{{\mathbb {R}}^2}({\mathcal {M}} \theta ^2)^\frac{p}{2} + |u |^p \, dx \, ds < \varepsilon , \end{aligned}$$

which, by choosing $\varepsilon$ as in Corollary 6.1, means that ${\mathcal {S}} \subset B_{M+4}(0) \times [0, T^*+4^{2\alpha }].$

*Step 2: Let* $\alpha \in (\alpha _0, \frac{1}{2})$ *(otherwise the dimension estimate is trivial by Remark* 6.7). *We show that for every* $q\ge 8$ *we have*

$$\begin{aligned} \dim _{b} {\mathcal {S}} \le \frac{1}{2\alpha - \frac{2}{q}} \left( \left( \frac{1+\alpha }{\alpha } + \frac{1}{q}\right) (1-2\alpha ) +2 \right) =: \beta (q). \end{aligned}$$

Indeed, fix $q \ge 8$ and define $p_q:= \frac{1+\alpha }{\alpha } + \frac{1}{q}.$ From Corollary 6.6, we know that if $(x,s) \in {\mathcal {S}}\,,$ then for every $r \in (0, (t/2)^\frac{1}{2\alpha })$ it holds

$$\begin{aligned}&\frac{1}{r^{p_q(1-2\alpha )+2}} \int _{{\mathcal {C}}^*(x,s; r)} y^b |\overline{\nabla } \theta ^* |^2 \, dz \, dy \, d\tau \\&\quad + \int _{{\mathcal {C}}(x,s;r)} |\theta ^\#_{\alpha , q} |^{1+\frac{1}{q^2-1}} \, dz \, d \tau > \varepsilon \Vert \theta \Vert _{L^\infty ( {\mathbb {R}}^2 \times [t/2, \infty ))}^{-(p-2)} =: 2\varepsilon _3 \,, \end{aligned}$$

where $\varepsilon =\varepsilon (\alpha )>0$ is universal and in particular independent of *r*. By Theorem 3.2, the threshold $\varepsilon _3$ depends on $t>0$ and $\Vert \theta _0\Vert _{L^2}$ only. Following Remark 6.5, we observe that with the notation of Remark 6.5

$$\begin{aligned} K_q(u; x,s,r) \le \max \left\{ 2C_2 \Vert \theta _0\Vert _{L^2} t^{-\frac{1}{2\alpha }(1-2/q)}, 1 \right\} =: L_q \end{aligned}$$

for $$r \in (0, \delta _0],$$ where

$$\begin{aligned} \delta _0:= \min \left\{ (t/2)^\frac{1}{2\alpha }, (L_q/2)^{1/(1-2\alpha +2/q)},1 \right\} . \end{aligned}$$

Observe that $L_q$ depends only on $\Vert \theta _0\Vert _{L^2}$ and $t>0.$ For $\delta \in (0, \delta _0)\,,$ we define the collection $\Gamma _\delta$ containing balls $B_{\sqrt{2} L_q \delta ^{2\alpha -2/q}}(x,s)$ centered at some point $(x,s) \in {\mathbb {R}}^2 \times [t, \infty )$ satisfying

$$\begin{aligned} \int _{ {\mathcal {C}}^*(x,s;\delta )} y^b |\overline{\nabla } \theta ^* |^2 \, dz \, dy \, d\tau + \int _{{\mathcal {C}}(x,s;\delta )} |\theta ^\#_{\alpha , q} |^{1 + \frac{1}{q^2-1}} \, dz \, d\tau \ge \varepsilon _3\, \delta ^{p_q(1-2\alpha )+2}. \end{aligned}$$

Observe that $\{\Gamma _\delta \}_\delta$ is a family of coverings of ${\mathcal {S}} \,$ consisting of Euclidean balls in spacetime. By the Vitali covering Lemma, there exists for every $\delta$ a countable, disjoint family $\{ B^i \}_{i \in I}$ such that

$$\begin{aligned} {\mathcal {S}} \subseteq \bigcup _{ i \in I } 5B^i \, \end{aligned}$$

and $B^i \in \Gamma _\delta \,,$ in particular $B^i=B_{\sqrt{2} L_q \delta ^{2\alpha - 2/q}}(x_i,s_i)$ for some $(x_i, s_i) \in {\mathbb {R}}^2 \times [t, \infty ).$ Observe that by Lemma 2.4, Theorem 2.1 and the global energy inequality (20) for Leray–Hopf weak solutions, we have the global control

$$\begin{aligned}&\int _0^\infty \int _{{\mathbb {R}}^3_+} y^b |\overline{\nabla } \theta ^*|^2 \, dz \, dy \, ds + \int _0^\infty \int _{{\mathbb {R}}^2} |\theta ^\#_{\alpha , q} |^{1+ \frac{1}{q^2-1}} \, dz \, ds \\&\quad \le C \int _0^\infty [\theta (s)]_{W^{\alpha ,2}({\mathbb {R}}^2)}^2 \, ds \le C \Vert \theta _0\Vert _{L^2}^2. \end{aligned}$$

Therefore, setting $\eta := 2\sqrt{2} L_q \delta ^{2\alpha - 2/q}$ and using the disjointness of $\{B^i\}_{i \in I}\,,$ we can estimate the minimal number $N(\eta )$ of sets of diameter $\eta$ needed to cover ${\mathcal {S}}$ by

$$\begin{aligned} N(\eta ) \le {\mathcal {H}}^0(I) \le \frac{C \Vert \theta _0\Vert _{L^2}^2}{\varepsilon _3 \delta ^{p_q(1-2\alpha ) +2}} = \frac{C \Vert \theta _0\Vert _{L^2}^2(\sqrt{2} L_q)^{\frac{p_q(1-2\alpha ) +2}{2\alpha - 2/q}}}{\varepsilon _3 \eta ^{\frac{p_q(1-2\alpha ) +2}{2\alpha - 2/q}}}. \end{aligned}$$

We conclude that

$$\begin{aligned} \dim _{b} {\mathcal {S}} =\limsup _{\eta \rightarrow 0} -\log _{\eta } N(\eta ) \le \frac{p_q(1-2\alpha ) +2}{2\alpha - 2/q}. \end{aligned}$$

*Step 3: Conclusion.*

By taking the limit $q \rightarrow \infty \,$ in Step 2, we conclude that

$$\begin{aligned} \dim _{b}(\mathrm{Sing} \, \theta \cap [t, \infty )) \le \frac{1}{2\alpha } \left( \frac{1+\alpha }{\alpha } (1-2\alpha ) + 2\right) \, \end{aligned}$$

for every $t>0$. Writing $\mathrm{Sing} \, \theta = \bigcup _{n \ge 1} \mathrm{Sing} \, \theta \cap \big [{\mathbb {R}}^2 \times \big [\frac{1}{n}, \infty \big )\big ]\,,$ we deduce that

$$\begin{aligned}&\dim _{\mathcal {H}}(\mathrm{Sing}\, \theta ) \le \sup _{n \ge 1} \, \dim _{\mathcal {H}}\left( \mathrm{Sing} \, \theta \cap \big [{\mathbb {R}}^2 \times \big [\tfrac{1}{n}, \infty \big )\big ] \right) \nonumber \\&\qquad \qquad \qquad \qquad \le \frac{1}{2\alpha } \left( \frac{1+\alpha }{\alpha } (1-2\alpha ) + 2\right) .\qquad \qquad \qquad \qquad \qquad \qquad \qquad \qquad \end{aligned}$$

$$\square $$

## Stability of the Singular Set

This section is devoted to the proof of Corollary 1.4. We observe that the $\varepsilon$-regularity criterion is “continuous” under strong $L^p$-convergence. This convergence result together with the observation that smooth solutions satisfy the $\varepsilon$-regularity criterion of Theorem 5.3 will allow to deduce the required stability.

### Lemma 8.1

Let $\alpha _n \in (\frac{1}{4}, \frac{1}{2})$ be such that $\alpha _n \rightarrow \alpha \in (\frac{1}{4}, \frac{1}{2}]$ and consider a sequence of suitable weak solutions $\theta _n$ to (1)–(3) with $\alpha = \alpha _n$ on ${\mathbb {R}}^2 \times [-2, 0]$ such that $\theta _n \rightarrow \theta$ strongly in $L^p( {\mathbb {R}}^2 \times [-2, 0])\,$ and assume that $\theta$ is a classical solution to (1)–(3) on ${\mathbb {R}}^2 \times [-2, 0]$. Then, there exists a universal constant $C>0$ such that uniformly for any $(x,t) \in Q_1$

$$\begin{aligned} \lim _{n \rightarrow \infty } E(\theta _{n,0}, u_{n,0}; \frac{1}{4} ) \le C E\left( \theta _0, u_0; \frac{1}{4}\right) \end{aligned}$$

where we denote by $\theta _{n,0}$ and $u_{n,0}$ (and $\theta _0$ and $u_0$ respectively) the change of variables of Lemma 5.1 as applied to $\theta _n$ (and $\theta$ respectively).

### Proof

We fix $(x,t) \in Q_1$ and apply the change of variables of Lemma 5.1 to $\theta _n$ and $\theta$ respectively. We denote the corresponding flow by $x_{n,0}$ and $x_0$. Moreover, we estimate for $s\in [-1,0]$

$$\begin{aligned}&| x_{n,0}(s) - x_0(s) | \le |s |^{1-\frac{1}{p}} \left( \int _{s}^0 \fint _{B_\frac{1}{4}(x+x_{n,0}(\sigma ))} |u_n(y, \sigma +t)- u(y, \sigma +t) |^p \, dy \, d\sigma \right) ^\frac{1}{p}\\&\qquad + \int _{s}^0 \fint _{B_\frac{1}{4}(x)} |u(y+x_{n,0}(\sigma ), \sigma +t) - u(y+ x_0(\sigma ), \sigma +t) |\, dy \,d\sigma \\&\quad \le 4^{\frac{2}{p}} |s |^{1-\frac{1}{p}} \left( \int _{t-1}^t \int |u_n-u |^p \, dy \, d\sigma \right) ^\frac{1}{p}+ \sup _{\sigma \in [t-1,t]}[u(\sigma )]_\mathrm{Lip({\mathbb {R}}^2)}\int _{s}^0 |x_{n,0}(\sigma )- x_{0}(\sigma ) |\, d\sigma . \end{aligned}$$

By Calderon–Zygmund, the strong convergence of $\theta _n$ implies that $u_n \rightarrow u$ strongly $L^p$. Calling $C= \sup _{\sigma \in [t-1,t]} [u(\sigma )]_\mathrm{Lip({\mathbb {R}}^2)}$ we deduce by Grönwall’s inequality that uniformly in $s \in [-1,0]$

59 $$\begin{aligned} \lim _{n\rightarrow \infty }|x_{n,0}(s)-x_0(s) |\le \lim _{n\rightarrow \infty } 4^{\frac{2}{p}} (1{+}Ce^C)\left( \int _{t-1}^t \int |u_n-u |^p \, dy \, d\sigma \right) ^\frac{1}{p} =0.\nonumber \\ \end{aligned}$$

We now claim that $\theta _{n,0} \rightarrow \theta _0$ strongly in $L^p({\mathbb {R}}^2 \times [-(1/4)^{2\alpha }, 0]).$ Fix $\varepsilon >0 \, .$ We split as before

$$\begin{aligned}&\int _{-\left( \frac{1}{4}\right) ^{2\alpha }}^0 \int |\theta _{n,0}- \theta _0 |^p (y,s) \, dy \, ds \\&\quad \le \int _{-\left( \frac{1}{4}\right) ^{2\alpha }}^0 \int |\theta _{n}(x_{n,0}(s)+y, s+t) - \theta (x_{n,0}(s)+y, s+t ) |^p \, dy \, ds \\&\qquad + \int _{-\left( \frac{1}{4}\right) ^{2\alpha }}^0 \int |\theta (x_{n,0}(s)+y, s+t) - \theta (x_0(s)+y, s+t) |^p \, dy \, ds. \end{aligned}$$

Using the strong convergence of $\theta _n$ in $L^p({\mathbb {R}}^2 \times [-2, 0])$, the first term on the right-hand side doesn’t exceed $\frac{\varepsilon }{3}$ for *n* big enough. Using (59) and absolute continuity, there exists $R\ge 1$ such that for all *n* big enough

$$\begin{aligned} \int _{-\left( \frac{1}{4}\right) ^{2\alpha }}^0 \int _{|y |\ge R} |\theta (x_{n,0}(s)+y, s+t) - \theta (x_0(s)+y, s+t) |^p \, dy \, ds \le \frac{\varepsilon }{3}. \end{aligned}$$

By the regularity of $\theta$ and (59), we estimate the remaining contribution of the integral over the set $\{|y |\le R\}$ by $[\theta ]_{\mathrm{Lip}({\mathbb {R}}^2 \times [t- (1/4)^{2\alpha }), t])}^p\Vert u_n-u\Vert _{L^p({\mathbb {R}}^2 \times [t-1,t])}^p R^p\,,$ which by the strong $L^p$-convergence does not exceed $\frac{\varepsilon }{3}$ for *n* big enough. The strong convergence of $\theta _{n,0} \rightarrow \theta _0$ in $L^p({\mathbb {R}}^2 \times [-(1/4)^{2\alpha }, 0])$ implies also that $u_{n,0} \rightarrow u_0$ strongly in $L^p({\mathbb {R}}^2 \times [-(1/4)^{2\alpha }, 0]).$ As an immediate consequence we obtain

$$\begin{aligned} \lim _{n \rightarrow \infty } E^S\left( \theta _{n,0}; \frac{1}{4}\right) + \lim _{n \rightarrow \infty } E^V\left( u_{n,0}; \frac{1}{4}\right) = E^S\left( \theta _0; \frac{1}{4}) + E^V(u_0; \frac{1}{4}\right) . \end{aligned}$$

and

$$\begin{aligned} \lim _{n \rightarrow \infty } E^{NL}\left( \theta _{n,0}; \frac{1}{4}\right)&\lesssim \lim _{n \rightarrow \infty } \Bigg \{ E^{NL}\left( \theta _0; \frac{1}{4}\right) + \left( \fint _{-(1/4)^{2\alpha }}^0 \sup _{R \ge \frac{1}{4}} \frac{1}{(4R)^{\sigma p}} \fint _{B_R} |\theta _{n,0}- \theta _0 |^p \right) ^\frac{1}{p} \\&\quad + \left( \fint _{-(1/4)^{2\alpha }}^0 |[\theta _0]_{B_\frac{1}{4}}- [\theta _{n,0}]_{B_\frac{1}{4}} |^p \right) ^\frac{1}{p} \Bigg \} \\&= E^{NL}\left( \theta _0; \frac{1}{4}\right) . \end{aligned}$$

$$\square $$

### Proof of Corollary 1.4

We argue by contradiction and we let $p:=\frac{6}{4\alpha -1}$ and $\sigma := \frac{4\alpha }{3}+\frac{1}{6}$ as in the proof of Corollary 6.1. We may assume that there is a sequence of orders $\alpha _n \in (\frac{1}{4}, \frac{1}{2})$ and initial data ${\bar{\theta }}_{n,0} \in H^2$ such that

- $$\lim _{n \rightarrow \infty } \alpha _n = \frac{1}{2} \,,$$
- $$\Vert {\bar{\theta }}_{n, 0}\Vert _{H^2({\mathbb {R}}^2)} \le R$$ for all $$n\ge 1\,,$$
- the local smooth solutions $\theta _n$ to (1)–(3) with $\alpha = \alpha _n$ and initial data ${\bar{\theta }}_{n,0}$, which exist on an interval $[0, T_1]$, with $T_1 = C \Vert {\bar{\theta }}_{n,0}\Vert _{H^2}^{-2} \ge C R^{-2}\,$ bounded from below uniformly in *n* (see [35]), blow-up in finite time.

Since $\theta _n \in L^\infty ([0, T_1], H^2)$ implies, for instance, that $\nabla \theta _n \in L^2([0, T_1], L^{4/\alpha })\,,$ $\theta _n$ satisifes the weak-strong uniqueness criterion of [15]^2^ and hence $\theta _n$ coincides on $[0,T_1]$ with the unique suitable weak solution to (1)–(3) with $\alpha = \alpha _n$ and initial data ${\bar{\theta }}_{n,0}$. We have the uniform bound

60 $$\begin{aligned} \sup _{n \ge 1} \Vert \theta _n\Vert _{L^\infty ({\mathbb {R}}^2 \times [T_1/2, \infty ))} + \sup _{n \ge 1}\Vert \theta _n\Vert _{L^\infty ([T_1/2, \infty ), L^2({\mathbb {R}}^2))} \le C \end{aligned}$$

given by (20) and Theorem 3.2. Up to subsequence, we have that ${\bar{\theta }}_{n,0} \rightarrow {\bar{\theta }}_0 \,$ strongly in $L^2({\mathbb {R}}^2).$ Fix $T>0.$ By (60), $\theta _n$ is uniformly bounded in $L^2({\mathbb {R}}^2 \times [T_1/2, T])$ and hence $\theta _n \rightharpoonup \theta$ weakly in $L^2.$ The strong convergence in $L^p({\mathbb {R}}^2 \times [T_1/2,T])$ is established as in the proof of Lemma 3.8, Step 2, using an Aubin-Lions type argument. The strong convergence for any $T>0$ is enough to pass to the limit the equation as well as the global energy inequalities (20)–(21) and hence $\theta$ is a Leray–Hopf solution to (1)–(3) with $\alpha = \frac{1}{2}$ and initial datum ${\bar{\theta }}_0$. This Leray–Hopf solution is smooth by [3]. The blow-up of the strong solutions means that there exists $(x_n, t_n) \in \mathrm{Sing} \, \theta _n\,$ for $n\ge 1.$ By Theorem 1.1 (and more precisely, noticing that Step 1 in its proof can be made uniform in *n*), there exists $M>0$ such that for all $n\ge 1$

$$\begin{aligned} (x_n, t_n ) \in {\overline{B}}_M \times [T_1, M]. \end{aligned}$$

Up to subsequence, we may assume that $(x_n, t_n) \rightarrow ({\bar{x}}, {\bar{t}}) \in {\overline{B}}_M \times [T_1, M].$ Rescaling the sequence of solutions by one single factor (related to $T_1$) and translating them, we may assume that $T_1 =- 2$ and that $({\bar{x}}, {\bar{t}})=(0,0) \in Q_1.$ By a further *n*-dependent temporal translation (by $2t_n\rightarrow 0$), we may assume that $t_n<0$. By the continuity of translations in $L^p$, we still have in this way that $\theta _n \rightarrow \theta$ strongly in $L^p({\mathbb {R}}^2 \times [-2, 0])$. We claim that there exists $r \in (0, 1]$ such that for any $(x,t) \in Q_1$

61 $$\begin{aligned} E\left( \theta _{r,0}, u_{r,0}; \frac{1}{4}\right) < \frac{\varepsilon _2}{2} \,, \end{aligned}$$

where we denote by $\theta _{r,0}$ and $u_{r,0}$ the change of variables of Lemma 5.1 applied to $(\theta _r, u_r)\,$ (see Remark 5.2) and by $\varepsilon _2$ the constant from Theorem 5.3. This now gives rise to a contradiction: Indeed, by Lemma 8.1, uniformly for every $(x,t) \in Q_1$, we have the lower semicontinuity $\lim _{n \rightarrow \infty } E((\theta _{n})_{r,0},( u_n)_{r,0}; \frac{1}{4}) \le C E(\theta _{r,0}, u_{r,0}; \frac{1}{4})$ such that for every *n* big enough, the smallness requirement of Theorem 5.3 holds and we deduce that $\theta _{n,r}$ is smooth in $Q_{1/2}$, namely $\theta _n$ is smooth in $Q_{r/2}.$ This contradicts the fact that the singular points $(x_n, t_n) \in Q_{r/2}$ for *n* big enough. We are thus left to prove (61). Indeed,

$$\begin{aligned} E^S\left( \theta _{r,0}; \frac{1}{4}\right)&= r^{2\alpha -1}\left( \fint _{r^{2\alpha }t-\left( \frac{r}{4}\right) ^{2\alpha }}^{r^{2\alpha }t} \fint _{B_\frac{r}{4}(rx+rx_0(r^{2\alpha }(s-t))} |\theta (y, s) - r^{1-2\alpha } (\theta _{r,0})_{Q_\frac{1}{4}}|^p \, dy \,ds \right) ^\frac{1}{p}\\&\le r^{4\alpha -1} [\theta ]_{\mathrm{Lip({\mathbb {R}}^2 \times [-(2r)^{2\alpha },0])}}\,, \end{aligned}$$

where we used again, as in the proof of Lemma 8.1, the fact that the flow $x_{0}$ is Lipschitz for a regular solution $\theta$. Similarly, $E^V(u_{r,0}; \frac{1}{4}) \le \, r^{2\alpha } [u]_{L^\infty ( [-(2r)^{2\alpha },0]; \mathrm Lip({\mathbb {R}}^2))}.$ Finally for the non-local part of the excess, we rewrite

$$\begin{aligned} \, E^{NL}\left( \theta _{r,0}; \frac{1}{4}\right) =&r^{2\alpha -1} \left( \fint _{- \left( \frac{r}{4}\right) ^{2\alpha }}^{0} \sup _{R \ge \frac{r}{16}} \left( \frac{r}{4R}\right) ^{\sigma p} \left( \fint _{B_R(x)} |\theta (y+x_0(r^{2\alpha }s), s+r^{2\alpha }t) \right. \right. \nonumber \\ {}&\left. \left. - r^{1-2\alpha } [\theta _{r,0}]_{B_\frac{1}{4}} |^\frac{3}{2} \, dy \right) ^\frac{2p}{3} \, ds \right) ^\frac{1}{p}. \end{aligned}$$

For fixed time $s\in [-(r/4)^{2\alpha }, 0]\,,$ we estimate the supremum splitting it on the two sets $\{\frac{1}{4} \ge R \ge \frac{r}{16} \}$ and $\{ R \ge \frac{1}{4} \}$. We get that

$$\begin{aligned} E^{NL}\left( \theta _{0, r}; \frac{1}{4}\right) \le C r^{\sigma p}([\theta ]_{\mathrm{Lip}({\mathbb {R}}^2 \times [-(2r)^{2\alpha }, 0])}+\Vert \theta \Vert _{L^\infty ({\mathbb {R}}^2 \times [-(2r)^{2\alpha }, 0])}). \end{aligned}$$

$$\square $$

## Appendix A. Local spacetime regularity of the fractional heat equation

### Lemma A.1

Let $\alpha \in (0, 1)$ and $p\ge 2$. Consider $\eta \in L^{3/2}_{loc}({\mathbb {R}}^2 \times [-1,0])$ with $(\eta )_{Q_1}=0$ and $E^S(\eta ;1)+E^{NL}(\eta ; 1)< +\infty$ which solves $\partial _t \eta + (-\Delta )^\alpha \eta = 0$ in $Q_{3/4}$. Then $\eta$ is smooth with respect to the space variable and $\eta \in C^{1-1/p}(Q_{1/2})$ in spacetime. Moreover, there exists ${\bar{C}}>1$ such that

$$\begin{aligned} \Vert \eta \Vert _{L^\infty ([-(1/2)^{2\alpha }, 0], C^1(B_\frac{1}{2}))} +\Vert \eta \Vert _{C^{1-\frac{1}{p}}(Q_\frac{1}{2})} \le {\bar{C}} ( E^S(\eta ;1) + E^{NL}(\eta ;1)). \end{aligned}$$

The constant ${\bar{C}}$ can be chosen uniform in $\alpha$ as long as $\alpha$ is bounded away from 0.

### Proof

Using the linearity of the equation, we can assume by a standard regularization argument that $\eta \in C^\infty ({\mathbb {R}}^2 \times (-1, 0))$. We multiply the equation by $\eta \varphi |_{y=0}$ where $\varphi$ is a smooth cut-off between $Q_{11/16}^*$ and $Q_{3/4}^*\,$ with $\partial _y \varphi (\cdot , 0, \cdot )=0$ and obtain, arguing as in Lemma 3.6,

$$\begin{aligned}&\int \eta ^2(x,t) \varphi (x, 0,t) \, dx + 2 c_\alpha \int _0^t \int y^b |\nabla \eta ^* |^2 \varphi \, dx \, dy \,ds \\&\quad = \int _0^t \int \eta ^2(x,s) \partial _t \varphi (x,0,s) \, dx \, ds \\&\qquad + c_\alpha \int _0^t \int y^b (\eta ^*)^2\overline{\Delta }_b \varphi \, dx \, dy \, ds. \end{aligned}$$

Taking the supremum over $t\in [-(11/16)^{2\alpha }, 0]$ and recalling the support of $\varphi$, we obtain by Lemma 3.9 that

62 $$\begin{aligned} \sup _{t\in [-(11/16)^{2\alpha }, 0]} \int _{B_\frac{11}{16}} \eta ^2(x,t) \, dx&\le C \left( \int _{Q_\frac{3}{4}} \eta ^2 \, dx \, ds + \int _{Q_\frac{3}{4}^*} y^b |\eta ^* |^2 \, dx \, dy \, ds \right) \nonumber \\&\le C \left( E^S(\eta ;1)^2+ E^{NL}(\eta ;1)^2 \right) . \end{aligned}$$

Let now *p*(*x*, *t*) be the fractional heat kernel on ${\mathbb {R}}^2\times (0, \infty )$ with explicit form $e^{-|\xi |^{2\alpha }t}$ in Fourier space. By scaling invariance, $p(x,t)= t^{-1/\alpha } p(t^{-1/(2\alpha )}x,1)$ and *p* is $C^\infty$ and bounded for $t>0.$ Let now $P^*(x,y):=[p(\cdot , 1)]^*(x,y)$ be the Caffarelli–Silvestre extension to ${\mathbb {R}}^{3}_+$ of $x \mapsto p(x,1)$ and observe that from scaling, $p^*(x,y,t) = t^{-1/\alpha } P^*(t^{-1/(2\alpha )}x,t^{-1/(2\alpha )}y).$ Fix a cut-off $\varphi$ between $B_{1/16}^*$ and $B_{1/8}^*$ which is radially symmetric in *x* and *y*. We define $\tilde{p}(x,y,t):= p^*(x,y,t) \varphi (x,y)\, .$ We proceed as in [31, Proposition 4.1] to obtain for $(x,t)\in B_{5/8}$

$$\begin{aligned} \eta (x,t)&= \int _{B_\frac{3}{4}} \eta ( z, -(3/4)^{2\alpha }) p(x-z, t+(3/4)^{2\alpha }) \varphi (x-z,0) \, dz \\&+ \int _{-(3/4)^{2\alpha }}^t \int _{B_\frac{3}{4}^*} y^b \eta ^*(x,y,\tau ) \overline{\Delta }_b \tilde{p}(x-z, y,t-\tau ) \,dz \, dy \, d\tau . \end{aligned}$$

We argue as in [31, Proposition 4.1] that $y^b\overline{\Delta }_b \tilde{p}(x,y,t) = \overline{{{\,\mathrm{div}\,}}}(y^b \overline{\nabla } \tilde{p}(x,y,t))$ is a smooth function in *x* and *y* supported in $B_{1/8}^* \setminus B_{1/16}*$ which remains bounded as $t \rightarrow 0.$ We conclude that for any multi-index $\beta$ with $|\beta |\ge 0$ we have, using (62) and Lemma 3.9, that

63 $$\begin{aligned} \Vert \partial _x^\beta \eta \Vert _{L^\infty (Q_\frac{5}{8})}&\lesssim \Vert \eta \Vert _{L^\infty L^2(Q_\frac{3}{4})} + \left( \int _{Q_\frac{3}{4}^*} y^b (\eta ^*)^2 \, dx \, dy \, dt \right) ^\frac{1}{2} \lesssim E^S(\eta ;1) + E^{NL}(\eta ; 1). \end{aligned}$$

To get the spacetime regularity, we observe that for $x\in B_{1/2}$ we can write (for fixed time)

$$\begin{aligned}&|(-\Delta )^\alpha \eta (x,t) |= \int _{|y |\le \frac{5}{8}} \frac{|\eta (x,t)- \eta (y,t) |}{|x-y |^{n+2\alpha }} \,dy + \int _{|y |> \frac{5}{8}} \frac{|\eta (x,t)- \eta (y,t)|}{|x-y |^{n+2\alpha }} \,dy \\&\quad \lesssim [\eta (t)]_\mathrm{Lip(B_\frac{5}{8})} + \sum _{i\ge 0} \frac{1}{2^{{ (i-1)(2\alpha -\sigma )}}} \int _{B_{2^{i+1}}\setminus B_{2^i}} \frac{|\eta (x,t) - \eta (y,t) |}{2^{(i-1)(n+\sigma )}} \, dy \\&\qquad + \int _{B_1 \setminus B_\frac{5}{8}}|\eta (x,t)- \eta (y,t) |\, dy \lesssim [\eta (t)]_\mathrm{Lip(B_\frac{5}{8})} \\&\quad + \sup _{R \ge 1} \frac{1}{R^\sigma } \fint _{B_R} |\eta (x, t) - [\eta (t)]_1 |\, dx + \int _{B_1} |\eta (x,t) |\,dx. \end{aligned}$$

Using (63) and the equation, we conclude

$$\begin{aligned} \Vert \partial _t \eta \Vert _{L^p L^\infty (Q_\frac{1}{2})} \lesssim E^S(\eta ;1) + E^{NL}(\eta ;1) \,, \end{aligned}$$

which proves that $\eta \in C^{1-\frac{1}{p}}([-(1/2)^{2\alpha }, 0], L^\infty (B_\frac{1}{2}))$ recalling that $W^{1,p}({\mathbb {R}}) \hookrightarrow C^{1- \frac{1}{p}}({\mathbb {R}}).$ $\square$

## Appendix B. $C^{\delta }$-Hölder continuous solutions are classical for $\delta >1-2\alpha$

In [9] it is proved that solutions of (1)–(3) with $u \in L^\infty ([0,T], C^\delta ({\mathbb {R}}^2))$, $\delta >1-2\alpha$, are smooth. The following Lemma provides a localized version of this result.

### Lemma B.1

Let $\theta : {\mathbb {R}}^2 \times (-1, 0] \rightarrow {\mathbb {R}}$ be a bounded solution of (1)–(3). If $\theta \in L^\infty ((-1, 0], C^{\delta }(B_2))$ for some $\delta > 1-2\alpha$, then $\theta \in C^{1, \delta -(1-2\alpha )}(B_{1/2 } \times (-1/2, 0])$ in spacetime and in particular, is a classical solution.

### Proof

*Step 1: We show that* $\theta \in L^\infty ([-1/2, 0], C^{1, \delta -(1-2\alpha )}(B_{1/2})).$

This follows from showing that $u \in L^\infty ((-1, 0], C^{ \delta }(B_1))$ and the general result on fractional advection-diffusion equations [31, Theorem 1.1]. Let us write $u= u_1+u_2$, where $u_1= {\mathcal {R}}^\perp (\theta \chi )$ and $u_2 = {\mathcal {R}}^\perp (\theta (1-\chi ))$ for $\chi$ a smooth cut-off in space between $B_{3/2}$ and $B_2.$ We estimate by Schauder estimates [29, Proposition 2.8]

$$\begin{aligned} \Vert u_1\Vert _{L^\infty ((-1, 0], C^{\delta })} \le C \Vert \theta \chi \Vert _{L^\infty ((-1, 0], C^{\delta })} \le C \Vert \theta \Vert _{L^\infty ((-1, 0], C^{\delta }(B_2))}. \end{aligned}$$

Regarding $u_2$, we observe that $[u_2(t)]_{C^k}$ is bounded, uniformly in $t\in (-1,0]$, for all $k\ge 1$. Indeed, consider for instance $k=1$: For $x \in B_1$ and fixed time, by integration by parts (the boundary term at infinity vanishes using the uniform boundedness of $\theta$)

$$\begin{aligned} \partial _j {\mathcal {R}}_i u_2(x)&= c \int \frac{x_i-z_i}{|x-z |^3} \partial _j ((1-\chi (z)) \theta (z) \, dz\\&= - c \int _{|z |\ge \frac{3}{2} } \partial _{z_j} \left( \frac{x_i-z_i}{|x-z |^3} \right) (1- \chi (z)) \theta (z) \, dz \,, \end{aligned}$$

so that, using that for $x \in B_1$ and $z \in B_{3/2}^c$ we have $|x - z |\ge \frac{1}{2}$, we have

$$\begin{aligned} |\partial _j {\mathcal {R}}_i u_2(x) |\le C \Vert \theta \Vert _{L^\infty }\int _{|x-z |\ge \frac{1}{2}} \frac{1}{|x-z |^3} \, dz < + \infty . \end{aligned}$$

We have obtained that

$$\begin{aligned} \Vert u\Vert _{L^\infty ((-1, 0], C^{ \delta }(B_1))} \le C (\Vert \theta \Vert _{L^\infty ((-1, 0], C^{ \delta }(B_2))}+ \Vert \theta \Vert _{L^\infty ({\mathbb {R}}^2 \times (-1, 0])}). \end{aligned}$$

*Step 2: We show that* $\theta \in C^{1, \delta -(1-2\alpha )}((-1/2, 0], L^\infty (B_{1/2})).$

Observe that $\theta$ solves a heat equation with right-hand side $u \cdot \nabla \theta \in L^\infty ((-1/2,0], C^{\delta -(1-2\alpha )}(B_{1/2}))$, so that

$$\begin{aligned} \partial _t \theta \in L^\infty ((-1/2,0], C^{\delta -(1-2\alpha )}(B_{1/2})). \end{aligned}$$

In particular, $\theta \in \mathrm{Lip}((-1/2, 0], C^{\delta -(1-2\alpha )}(B_{1/2})).$ Repeating the argument, we obtain that $\theta \in C^{1, \delta -(1-2\alpha )}(B_{1/2} \times (-1/2, 0]).$ Higher regularity then follows from energy estimates. $\square$

## Existence of Suitable Weak Solutions

### Proof of Theorem 3.6

Fix $\theta _0 \in L^2({\mathbb {R}}^2)$. For $\epsilon >0$, we consider the system with added vanishing viscosity term

64 $$\begin{aligned} {\left\{ \begin{array}{ll} \partial _t \theta + u\cdot \nabla \theta + (-\Delta )^\alpha \theta = \epsilon \Delta \theta \\ u = \nabla ^\perp (-\Delta )^{-\frac{1}{2}} \theta \,, \end{array}\right. } \end{aligned}$$

complemented with the initial datum $\theta (\cdot , 0) = \theta _0\, .$ For any $\epsilon >0$, the system (64) admits a global smooth solution $\theta _\epsilon :{\mathbb {R}}^2\times (0, \infty ) \rightarrow {\mathbb {R}}$. Moreover, for any $t>0$, $\theta _\epsilon (\cdot , t) \in L^2({\mathbb {R}}^2)$ and for any $0 \le s< t$, we have the energy equality

65 $$\begin{aligned} \int \theta _\epsilon ^2(x,t) \, dx + 2 \int _s^t \int \left[ |(-\Delta )^\frac{\alpha }{2}\theta _\epsilon |^2(x, \tau ) + \epsilon |\nabla \theta _\epsilon |^2 (x,\tau ) \right] \, dx \, d\tau = \int \theta _\epsilon ^2(x,s) \, dx.\nonumber \\ \end{aligned}$$

Theorem 3.2 also applies to (64), so that $\theta _\epsilon$ is in $L^\infty$ for $t>0$ with the uniform-in-$\epsilon$ bound

66 $$\begin{aligned} \Vert \theta _\epsilon (t)\Vert _{L^\infty } \le Ct^{-\frac{1}{2\alpha }} \Vert \theta _0\Vert _{L^2}. \end{aligned}$$

Finally, with the obvious modifications of the computation in Section 3.3, we have for any nonnegative test function $\varphi \in C^\infty _c({\mathbb {R}}^3_+)$, locally constant in *y* in a neighbourhood of $y=0$, any nonnegative and convex $f\in C^2({\mathbb {R}})$ and any $t>0$ that

$$\begin{aligned}&\int _{{\mathbb {R}}^2} \varphi (x,0,t) f(\theta _\epsilon ) (x,t) \, dx +c_{\alpha }\int _0^t \int _{{\mathbb {R}}^3_+} y^b|\overline{ \nabla } \theta _\epsilon ^* |^2 f''(\theta _\epsilon ^*)\varphi \, dx \, dy \, ds\\&\quad \le \int _0^t \int _{{\mathbb {R}}^2} \left[ f( \theta _\epsilon ) (\partial _t \varphi |_{y=0}+ \epsilon \Delta \varphi |_{y=0}) + u_\epsilon f( \theta _\epsilon ) \cdot \nabla \varphi |_{y=0} \right] \, dx \, ds \\&\qquad + c_{\alpha } \int _0^t\int _{{\mathbb {R}}^3_+} y^b f( \theta _\epsilon ^*) {\overline{\Delta }}_b \varphi \, dx \, dy \, ds \, \\&\quad =: C(\epsilon ) + D(\epsilon ). \end{aligned}$$

We want to pass to the limit $\epsilon \rightarrow 0$. From (65) with $s=0$ and the Sobolev embedding of ${\dot{W}}^{\alpha , 2}({\mathbb {R}}^2) \hookrightarrow L^\frac{2}{1-\alpha }({\mathbb {R}}^2)$, we infer by interpolation that the family $\{\theta _\epsilon \}_{\epsilon >0}$ is uniformly bounded in

$$\begin{aligned} L^\infty ([0, \infty ), L^2) \cap L^2([0, \infty ), {\dot{W}}^{\alpha ,2}) \hookrightarrow L^{2(1+\alpha )}({\mathbb {R}}^2 \times [0, \infty )). \end{aligned}$$

By Banach-Anaoglu, for any fixed time $T>0$, there exists $\theta \in L^2({\mathbb {R}}^2 \times [0, T])$ and a subsequence $\epsilon _k \rightarrow 0$ such that $\theta _{\epsilon _k} \rightharpoonup \theta$ weakly in $L^2({\mathbb {R}}^2 \times [0, T])$. We now claim that this convergence is in fact strong via an Aubin-Lions type argument in the same spirit as Step 2 of the proof of Lemma 3.8. Fix $\eta >0$ and a family of mollifiers $\{\phi _\delta \}_{\delta \ge 0} \subseteq C^\infty _c({\mathbb {R}}^2)$ in space. For $k,j \ge 1$ we estimate

$$\begin{aligned} \Vert \theta _{\epsilon _j}- \theta _{\epsilon _k}\Vert _{L^2({\mathbb {R}}^2 \times [0, T])} \le \Vert \theta _{\epsilon _j} - \theta _{\epsilon _j} *\phi _\delta \Vert _{L^2} + \Vert \theta _{\epsilon _k} - \theta _{\epsilon _k} *\phi _\delta \Vert _{L^2} + \Vert (\theta _{\epsilon _j}-\theta _{\epsilon _k}) *\phi _\delta \Vert _{L^2}. \end{aligned}$$

The first two contributions converge to 0 independently of *k* and *j* due to a bound of the form $\Vert \theta _{\epsilon _k}-\theta _{\epsilon _k} *\phi _\delta \Vert _{L^2({\mathbb {R}}^2 \times [0, T])}^2\le C \delta ^{2\alpha }$ obtained as in (27) with $\eta _k$ replaced by $\theta _{\varepsilon _k}$. We now choose $\delta$ small enough such that this contribution does not exceed $\frac{\eta }{3}.$ Having $\delta$ fixed, we claim that the family of curves $\{ t \mapsto \theta _{\epsilon _k}(\cdot , t) \}_{k \ge 1}\,$ is equicontinuous and equibounded with values in $W^{1, \infty }$. Indeed, by the energy equality (65) with $s=0$ and the Calderon–Zygmund estimate $\Vert u_{\epsilon _k}\Vert _{L^2({\mathbb {R}}^2 \times [0, T])} \le C\Vert \theta _{\epsilon _k}\Vert _{L^2({\mathbb {R}}^2 \times [0, T])}$, we estimate

$$\begin{aligned} \Vert \partial _t \theta _{\epsilon _k} *\phi _{\delta }\Vert _{L^2([0, T],W^{1, \infty })}&\le \Vert {{\,\mathrm{div}\,}}(u_{\epsilon _k} \theta _{\epsilon _k}) *\phi _\delta \Vert _{L^2 W^{1, \infty }} + \Vert \theta _{\epsilon _k} *(-\Delta )^\alpha \phi _\delta \Vert _{L^2 W^{1, \infty }} \\&\quad + \epsilon _k \Vert \theta _{\epsilon _k} *\Delta \phi _\delta \Vert _{L^2 W^{1, \infty }} \\&\le \left( \Vert u_{\epsilon _k}\Vert _{L^2({\mathbb {R}}^2 \times [0, T])} \Vert \theta _0\Vert _{L^2} +2\Vert \theta _{\epsilon _k}\Vert _{L^2({\mathbb {R}}^2 \times [0, T])} \right) \Vert \phi _\delta \Vert _{W^{3, \infty }} \\&\le C(\delta ). \end{aligned}$$

By Ascoli-Arzela the sequence $\{\theta _k *\phi _\delta \}_{k \ge 1}$ converges uniformly on ${\mathbb {R}}^2 \times [0, T]\,$ and by uniqueness of limits, we infer that this limit must coincide with $\theta *\phi _\delta .$ We can therefore choose $N\ge 1$ big enough such that for any $k, j \ge N$ we have $\Vert (\theta _{\epsilon _j}- \theta _{\epsilon _k}) *\phi _\delta \Vert _{L^2({\mathbb {R}}^2 \times [0,T])} \le \frac{\eta }{3}.$ We conclude that for $k, j \ge N$ there holds $\Vert \theta _{\epsilon _k}- \theta _{\epsilon _j}\Vert _{L^2({\mathbb {R}}^2 \times [0, T])} \le \eta .$ Since $\eta$ was arbitrary, we conclude by uniqueness of limits that $\theta _{\epsilon _k} \rightarrow \theta$ strongly in $L^2({\mathbb {R}}^2 \times [0, T]).$ By the uniform boundedness in $L^{2(1+\alpha )}({\mathbb {R}}^2 \times [0, \infty ))$ and by (66) we also deduce that $\theta _k \rightarrow \theta$ strongly in $L^r({\mathbb {R}}^2 \times [0, T])$ for any $2 \le r < 2(1+\alpha )$ and strongly in $L^r({\mathbb {R}}^2 \times [\tau , T])$ for any $\tau >0$ and any $2 \le r < \infty$. By Calderon–Zygmund, we infer that $u_\epsilon \rightarrow u:= {\mathcal {R}}^\perp \theta$ strongly in $L^2({\mathbb {R}}^2\times [0, T])$ (and $L^r$ respectively). Passing to the limit $k \rightarrow \infty$ in the equation (64), we infer that $\theta$ is a distributional solution to (1)–(3). We are left to pass to the limit in the global and local energy (in-)equality. Consider first (65). By Banach Anaoglu and uniqueness of limit $(-\Delta )^\frac{\alpha }{2} \theta _{\epsilon _k} \rightharpoonup (-\Delta )^\frac{\alpha }{2} \theta$ weakly in $L^2({\mathbb {R}}^2 \times [0, T])$ and by weak lower semicontinuity

$$\begin{aligned} \int _s^t \int |(-\Delta )^\frac{\alpha }{2} \theta |^2(x, \tau ) \, dx \, d \tau \le \liminf _{k\rightarrow \infty }\int _s^t \int |(-\Delta )^\frac{\alpha }{2} \theta _{\epsilon _k} |^2(x, \tau ) \, dx \, d \tau \end{aligned}$$

for any $0 \le s < t.$ For almost every $t \in [0,T]$ we can extract a further subsequence such that $\theta _{\epsilon _k}(\cdot , t) \rightarrow \theta (\cdot , t)$ strongly in $L^2({\mathbb {R}}^2)$. By passing to the limit in (65), we thereby obtain (20) and (21) for almost every $0<s<t.$ We obtain it for every $t>0$ (and almost every $0<s<t$) by observing that up to changing $\theta$ on a set of measure 0, we may assume that $\theta$ is continuous with respect to the weak topology on $L^2({\mathbb {R}}^2).$ We are left to pass to the limit in the localized energy inequality for $f(x)= \frac{(x-M)^2}{L^2}$ and $f(x)= |\frac{x-M}{L} |^q$ for $q >2$ respectively, with some $L>0$ and $M \in {\mathbb {R}}$. Let us denote $\eta _k := (\theta _{\epsilon _k}-M)/L$, $\eta := (\theta -M)/L\,$ and let us fix $\tau , R >0$ such that ${{\,\mathrm{supp}\,}}\varphi \subseteq B_R(0) \times [0, R] \times [\tau , T].$ From the strong convergence established before, we infer that $\eta _k \rightarrow \eta$ strongly in $L^r_{loc}({\mathbb {R}}^2 \times [ \tau , T])\,$ for $2 \le r < \infty .$ Up to extracting a further subsequence and a diagonal argument, we obtain that $\eta _k(t) \rightarrow \eta (t)$ strongly in $L^r_{loc}({\mathbb {R}}^2)$ for almost every $t>0$ and any $r \in {\mathbb {N}}_{\ge 2}$. By interpolation, the former statement holds in fact for every $2 \le r < \infty .$ We deduce that for almost every $t>0\,,$ for $q=2$ and any $q\ge 4$

$$\begin{aligned}&\lim _{k \rightarrow \infty } \int _{{\mathbb {R}}^2} \varphi (x,0,t) f( \theta _{\epsilon _k})^2(x,t) \, dx =\lim _{k \rightarrow \infty } \int _{{\mathbb {R}}^2} \varphi (x,0,t) |\eta _k |^q(x,t) \, dx \\&\quad = \int _{{\mathbb {R}}^2} \varphi (x,0,t) |\eta |^q(x,t) \, dx \,, \\&\lim _{k \rightarrow \infty } C(\epsilon _k) = \int _0^t \int _{{\mathbb {R}}^2} \left[ |\eta |^q \partial _t \varphi |_{y=0} + u |\eta |^q \cdot \nabla \varphi |_{y=0} \right] \, dx \, ds. \end{aligned}$$

We recall that from the Poisson formula $\theta _{\epsilon _k}^*(x,y,t) =( P(\cdot ,y) *\theta _{\epsilon _k}(\cdot , t))(x)$ and Young’s convolution inequality

$$\begin{aligned} \Vert \theta _{\epsilon _k}^*\Vert _{L^q({\mathbb {R}}^2 \times [0, R] \times [\tau , T],\, y^b)} \le C(R) \Vert P(1, \cdot )\Vert _{L^1} \Vert \theta _{\epsilon _k}\Vert _{L^q({\mathbb {R}}^2 \times [\tau , T])}= C(R, \alpha )\Vert \theta _{\epsilon _k}\Vert _{L^q({\mathbb {R}}^2 \times [\tau , T])} \,, \end{aligned}$$

where we used that $\Vert P(\cdot , y)\Vert _{L^1}= \Vert P(\cdot , 1)\Vert _{L^1}$ for $y>0.$ We deduce that $\theta _{\epsilon _k}^* \rightarrow \theta ^*$ strongly in $L^q({\mathbb {R}}^2\times [0, R] \times [\tau , T], y^b).$ By linearity, $\eta _k^* = \frac{\theta _{\epsilon _k}^*-M}{L}$ so that $\eta _k^* \rightarrow \eta ^*$ strongly in $L^q(B_R \times [0, R] \times [\tau , T], y^b).$ Hence

$$\begin{aligned} \lim _{k \rightarrow \infty } D(\epsilon _k) = \int _0^t \int _{{\mathbb {R}}^3_+} y^b |\eta ^*|^q \overline{\Delta }_b \varphi \, dx \, dy \, ds. \end{aligned}$$

Moreover, we also deduce $\overline{\nabla } \eta _k^* \rightharpoonup \overline{\nabla } \eta ^*$ and$\overline{\nabla } |\eta _k^* |^\frac{q}{2} \rightharpoonup \nabla |\eta ^* |^\frac{q}{2}$ weakly in $L^2(B_R(0) \times [0, R] \times [\tau , T], y^b)\,$ and we infer by weak lower semicontinuity and the positivity of $\varphi$ that

$$\begin{aligned} \int _0^t \int _{{\mathbb {R}}^3_+} y^b |\overline{\nabla } \eta ^* |^2 \varphi \, dx \, dy \, ds&\le \liminf _{k \rightarrow \infty } \int _0^t \int _{{\mathbb {R}}^3_+}y^b |\overline{\nabla } \eta _k^* |^2 \varphi \, dx \, dy \,ds \\ \int _0^t \int _{{\mathbb {R}}^3_+} y^b |\overline{\nabla } |\eta ^* |^\frac{q}{2} |^2 \varphi \, dx \, dy \, ds&\le \liminf _{k \rightarrow \infty } \int _0^t \int _{{\mathbb {R}}^3_+}y^b |\overline{\nabla } |\eta _k^* |^\frac{q}{2} |^2 \varphi \, dx \, dy \,ds \end{aligned}$$

for any $t >0.$ Passing to the limit $k \rightarrow \infty$, we obtain (25) and (26) for almost every $t>0.$ $\square$

## Footnotes

1 That is, the function $\varphi$ vanishes when $|x| + y + |t|$ is large enough and if *t* is sufficiently close to 0, but it can be nonzero on some regions of $\{(x,y,t): y=0\}$.

2 The authors of [15] state their result in the form of an asymptotic stability result with respect to perturbations of the initial datum and the right-hand side; however, in absence of any perturbation their energy yield the corresponding weak-strong uniqueness statement, as previously observed for instance in [25].
